# CLIPS-1D: analysis of multiple sequence alignments to deduce for residue-positions a role in catalysis, ligand-binding, or protein structure

**DOI:** 10.1186/1471-2105-13-55

**Published:** 2012-04-05

**Authors:** Jan-Oliver Janda, Markus Busch, Fabian Kück, Mikhail Porfenenko, Rainer Merkl

**Affiliations:** 1 Institute of Biophysics and Physical Biochemistry, University of Regensburg, 93040 Regensburg, Germany; 2 Faculty of Mathematics and Computer Science, University of Hagen, 58084 Hagen, Germany

## Abstract

**Background:**

One aim of the *in silico *characterization of proteins is to identify all residue-positions, which are crucial for function or structure. Several sequence-based algorithms exist, which predict functionally important sites. However, with respect to sequence information, many functionally and structurally important sites are hard to distinguish and consequently a large number of incorrectly predicted functional sites have to be expected. This is why we were interested to design a new classifier that differentiates between functionally and structurally important sites and to assess its performance on representative datasets.

**Results:**

We have implemented CLIPS-1D, which predicts a role in catalysis, ligand-binding, or protein structure for residue-positions in a mutually exclusive manner. By analyzing a multiple sequence alignment, the algorithm scores conservation as well as abundance of residues at individual sites and their local neighborhood and categorizes by means of a multiclass support vector machine. A cross-validation confirmed that residue-positions involved in catalysis were identified with state-of-the-art quality; the mean MCC-value was 0.34. For structurally important sites, prediction quality was considerably higher (mean MCC = 0.67). For ligand-binding sites, prediction quality was lower (mean MCC = 0.12), because binding sites and structurally important residue-positions share conservation and abundance values, which makes their separation difficult. We show that classification success varies for residues in a class-specific manner. This is why our algorithm computes residue-specific *p*-values, which allow for the statistical assessment of each individual prediction. CLIPS-1D is available as a Web service at http://www-bioinf.uni-regensburg.de/.

**Conclusions:**

CLIPS-1D is a classifier, whose prediction quality has been determined separately for catalytic sites, ligand-binding sites, and structurally important sites. It generates hypotheses about residue-positions important for a set of homologous proteins and focuses on conservation and abundance signals. Thus, the algorithm can be applied in cases where function cannot be transferred from well-characterized proteins by means of sequence comparison.

## Background

It is of general interest to identify important sites of a protein, for example when elucidating the reaction mechanism of an enzyme. To support this task, classifiers have been developed, which utilize different kinds of information about the protein under study. Some algorithms are based on sequences [1-11], other ones make use of 3D-data [12,13], and a third class combines both approaches [14-18].

A strong argument in favor of sequence-based methods is their broad applicability and their potential to characterize proteins with a novel fold. Additionally, some signals seem to be more pronounced in sequence- than in 3D-space [19]. Commonly, these methods depend on a multiple sequence alignment (MSA) composed of a sufficiently large number of homologs. Based on the assumption that critical residues are not altered during evolution, the canonical feature to identify important residue-positions in an MSA is the conservation of individual columns. The degree of conservation can help to predict a role: In many cases, strictly conserved residues are essential for protein function [7,20,21]. In contrast, a prevalent but not exclusively found amino acid is often important for protein stability [22,23], which similarly holds for ligand-binding sites. Thus, for a precise discrimination, several properties have to be interpreted. Features that improve prediction of functionally important sites are the conservation of proximate residues [7,24] and the abundance of amino acid residues observed at catalytic sites [8,24]. In addition, implicit features deduced from protein sequences have been utilized, like the predicted secondary structure and the predicted solvent accessible surface of residues [5,8].

Most of the existing algorithms focus on the identification of sites relevant for protein function. In order to broaden the classification spectrum, we implemented the sequence-based algorithm CLIPS-1D, which predicts functionally important sites in addition to residue-positions crucial for protein structure in a mutually exclusive manner. It is based on a multiclass support vector machine, which assesses not more than seven properties deduced from residue-positions and their local neighborhood in sequence space. Our approach compares favorably with state-of-the-art classifiers and predicts catalytic residue-positions with a mean MCC-value of 0.34. The mean MCC-value is for structurally important sites 0.67 and for ligand-binding sites it is 0.12. Our findings show that separating ligand-binding sites and structurally important sites is difficult due to their similar properties and that classification quality depends on the residue type.

## Results and discussion

### Analysis of local conservation and abundance signals allows for a state-of-the-art classification

High-quality datasets consisting of catalytic sites, ligand-binding sites, and sites important for protein structure are required to train and assess support vector machines (SVMs), which predict the respective roles of residue-positions. Based on the content of EBI-databases, we prepared the redundancy-free and non-overlapping sets *CAT_sites *and *LIG_sites*, which consist of 840 catalytic sites and 4466 ligand-binding sites deduced from a set of 264 enzymes named *ENZ *(see Methods). Whereas the full set of functionally important sites is known for many enzymes, residues that crucially determine structure have not been identified for a representative set of proteins. Thus, to compile such sites, we had to follow an indirect approach [25] by assuming that residues in the core of proteins lacking enzymatic function are conserved due to their relevance for structure. This notion is supported by the fact that conserved hydrophobic core-residues can contribute substantially to protein stability [26]. By re-annotating a comprehensive set of non-enzymes from reference [27], we culled the dataset *NON_ENZ*, which consists of 136 proteins. *NON_ENZ *contains 3703 buried residue-positions, which are more conserved than the mean (see Methods); we designated these sites *STRUC_sites*. For all proteins under study, MSAs were taken from the HSSP database [28] and filtered prior to analysis.

Next, we identified features, which allow for a state-of-the-art classification of *CAT_sites*, *LIG_sites*, and *STRUC_sites*. Thus, we trained three two-class (2C-) SVMs to predict for each residue-position *k*, whether it is important for catalysis (*SVM_CAT_*), ligand-binding (*SVM_LIG_*), or protein structure (*SVM_STRUC_*) and compared performance values. In the end, the features used to characterize each *k *were in the case of *SVM_CAT _*a normalized Jensen-Shannon divergence *cons_JSD _*(*k*) (formula (4)) and an abundance-value *abund*(*k, CAT_sites*) scoring the occurrence of residues at *CAT_sites *according to formula (6). The proximity of *k *was assessed by means of a weighted score *cons_neib_*(*k*) (formula (5)) and a novel abundance-value $abund_{neib}\left(aa_{s}^{k},CAT\text{\_}sites\right)$, deduced from conditional frequencies in the ± 3 neighborhood [8] of *CAT_sites *(formula (7)). Thus, $abund_{neib}\left(aa_{s}^{k},CAT\text{\_}sites\right)$ compares the local environment of site *k *with the one observed for residues $aa_{s}^{k}$ at positions annotated as catalytic sites. In order to quantify the contribution of individual features to classification quality, performance was determined for SVMs exploiting either all four features or a combination of three features, respectively. Analogously, scores for *LIG_sites *were computed, and *SVM_LIG _*was trained and assessed.

It is difficult to unambiguously determine a classifier's performance, if the numbers of positive and negative cases differ to a great extent, as is here the case. This is why we computed a battery of performance values, which are given in Additional file 1: Table S1. Their comparison confirms for our problem that the performance measures support each other, thus we focus on MCC-values [29], which are also listed in Table 1. The MCC-values for *SVM_CAT _*and *SVM_LIG _*were 0.324 and 0.213, respectively. MCC-comparison makes clear that for *CAT_sites *and *LIG_sites *all four features add to classification quality. For *CAT_sites*, *cons_JSD _*(*k*) and *abund*(*k, CAT_sites*) contributed most, for *LIG_sites*, the conservation score *cons_JSD_*(*k*) was most relevant; compare Additional file 1: Table S1 and Additional file 1: Figure S1, which shows ROC and PROC curves.

**Table 1:** Classification performance of SVMs and FRpred on functionally and structurally important residue-positions

	*CAT_sites*	*LIG_sites*	*STRUC_sites*
2C-SVM	0.324	0.213	0.782

CLIPS-1D	0.337	0.117	0.666

FRpred, score ≥ 8	0.231	0.219	41%

FRpred, score = 9	0.250	0.197	22%

The line "2C-SVM" gives MCC-values resulting from a classification of catalytic sites (*CAT_sites*) with *SVM_CAT_*, of ligand-binding sites (*LIG_sites*) with *SVM_LIG_*, and of structurally important sites (*STRUC_sites*) with *SVM_STRUC_*. The line "CLIPS-1D" shows the performance of the MC-SVM. For FRpred, performance resulting from the analysis of HSSP-MSAs is given. For *CAT_sites *and *LIG_sites*, MCC-values are listed resulting from FRcons-cat or FRcons-lig scores of at least 8 or 9, respectively. For *STRUC_sites*, the same percentage of false positives resulted from FRcons-cat and FRcons-lig predictions.

Can *SVM_CAT _*and *SVM_LIG _*compete with state-of-the-art classifiers? For the assessment, we selected FRpred, which has outperformed other approaches and which additionally exploits the predicted secondary structure and solvent accessibility [8]. It has reached 40% precision at 20% sensitivity for the identification of catalytic residues and is accessible as a Web service [8]. FRpred lists two subtypes of predictions, FRcons-cat for catalytic sites and FRcons-lig for ligand-binding sites. All results are scored with values of 0-9; the higher the score, the more probable is a functional role of the residue. A classification of *CAT_sites *and *LIG_sites *with FRpred resulted in MCC-values of 0.250 (FRcons-cat) and 0.197 (FRcons-lig), when considering predictions scored 9 as positive cases. For predictions scored at least 8, the MCC-values were 0.231 and 0.219, respectively. Interestingly, performance was better, when we uploaded our preprocessed HSSP-MSAs than when FRpred compiled MSAs on itself (compare Additional file 1: Table S1), which indicates the high quality of these specifically filtered MSAs. In summary, the comparison of performance values for FRpred, *SVM_CAT_*, and *SVM_LIG _*confirmed that the four features selected by us account for a state-of-the-art classification.

Using corresponding features and the set *STRUC_sites*, we analogously trained *SVM_STRUC _*for the prediction of residue-positions important for structure, which gave an MCC-value of 0.761. Classification quality was determined to the greatest extent by *cons_JSD _*(*k*). When classifying without this feature, MCC was lowered to 0.346. Utilizing the feature *abund_neib_*(*k, STRUC_sites*) deteriorated performance; a higher MCC-value (0.782) was gained by an SVM trained on the remaining three features. Even *abund*(*k, STRUC_sites*) had only a marginal effect, although the respective scores differ considerably from those of *abund*(*k, CAT_sites*) and *abund*(*k, LIG_sites*); compare Table 2 and Additional file 1: Figure S2. Thus, in proteins without enzymatic function, the assessment of conservation contributed most to separate the conserved buried residues from all other ones, which constitute the negative cases. FRpred predicted with score 9 22% and with score 8 41% of the *STRUC_sites *as catalytic sites or ligand-binding sites; see Table 1.

**Table 2:** *abund*(*k, CLASS*)-values for amino acid residues

Residue	*CAT_sites*	*LIG_sites*	*STRUC_sites*
A	-2.0424	-0.3537	-0.1210

C	1.3255	0.7376	1.2398

D	1.1178	0.0426	-0.0498

E	0.6536	-0.3856	-0.6615

F	-0.7708	-0.0081	0.5057

G	-0.7533	0.4195	0.7020

H	1.8883	0.8279	-0.3044

I	-2.8164	-0.3026	-0.6449

K	0.6051	-0.3615	-1.0215

L	-2.4503	-0.5416	0.2116

M	-1.4026	0.1374	-0.4882

N	-0.1972	0.3566	-0.2254

P	-5.0000	-0.4542	0.3643

Q	-0.7243	-0.1841	-0.5615

R	0.6834	0.3879	-0.2593

S	0.0027	-0.0125	-0.7006

T	-0.5435	0.2314	-0.3363

V	-2.9568	-0.4130	-0.3294

W	0.1927	0.5548	1.2811

Y	0.3265	0.4572	0.7058

The score-values were deduced from residues belonging to the respective classes. See formula (6) for a definition of the scores.

### CLIPS-1D: Towards a more diversified prediction of residue function

**Figure 1:**
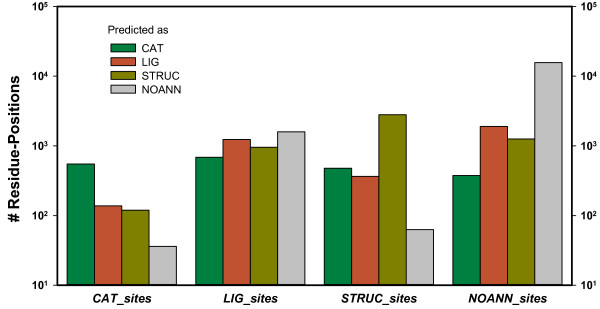
**Classification performance of CLIPS-1D in predicting functionally and structurally important residue-positions**. Based on the maximal class-probability *p_class _*all members of the classes *CAT_sites*, *LIG_sites*, *STRUC_sites*, and *NOANN_sites *were categorized. *NOANN_sites *are all residue-positions not selected as *STRUC_sites *in the *NON_ENZ *dataset, *i.e*. positions without assigned function. Note that the absolute numbers of residue-positions are plotted with a logarithmic scale.

In order to elaborate the subtle differences distinguishing functionally and structurally important residue-positions, all combinations of the above training sets have to be exploited. This is why we prepared a multi-class support vector machine (MC-SVM) for CLIPS-1D, which was trained on the four classes *CAT_sites*, *LIG_sites*, *STRUC_sites*, and *NOANN_sites*, *i.e*., all residue-positions from *NON_ENZ *not selected as *STRUC_sites*. Due to the above findings on 2C-SVMs, we chose the following seven features: *cons_JSD _*(*k*), *cons_neib_*(*k*), *abund*(*k, CAT_sites*), *abund*(*k, LIG_sites*), *abund*(*k, STRUC_sites*), *abund_neib_*(*k, CAT_sites*), and *abund_neib_*(*k, LIG_sites*). The MC-SVM outputs a list of four class-specific probability values *p_class_*. Based on the largest *p_class_*-values, residue-positions were assigned one of the four classes; the resulting distributions are shown in Figure 1. 65% of the *CAT_sites *and 76% of the *STRUC_sites *were correctly assigned. 64% of the *LIG_sites *and 19% of *NOANN_sites *were misclassified, and each class contributed a noticeable fraction of false positives. 13% of the *STRUC_sites *were classified as *CAT_sites *and 10% as *LIG_sites*. Although the algorithm frequently failed to assign the correct class, separating positions with and without a crucial role was more successful: 96% of the *CAT_sites*, 65% of the *LIG_sites*, and 98% of the *STRUC_sites *were classified as structurally or functionally important and 81% of the *NOANN_sites *were classified as having no crucial function. It turned out that the respective MCC-value was optimal, if *CAT_sites *with *p_CAT_*(*k*) > 0.61 were selected as positives. In summary, the corresponding MCC-values were 0.337, 0.117, and 0.666 for *CAT_sites*, *LIG_sites*, and *STRUC_sites*; see Table 1. In comparison with 2C-SVMs, the performance on *CAT_sites *improved moderately. However, the performance on *LIG_sites *and *STRUC_sites *dropped, which indicates that the separation of *LIG_sites *and *STRUC_sites *is difficult.

The comparison of *abund*()-values (compare Table 2) makes clear that residues are unevenly distributed among the classes, which must influence the residue-specific classification quality. Thus, we determined class-specific MCC-values for each residue, which are listed in Table 3. As expected, performance differs drastically for individual residues and between classes. Among *CAT_sites*, Arg, Asp, Cys, His, Lys, and Ser were predicted with high quality. Most of the other MCC-values were near zero and no MCC-value could be computed for Pro and Val due to empty sets. The performance-values for *LIG_sites *were generally lower. Among *STRUC_sites*, the mean MCC-value for the hydrophobic residues Ala, Ile, Leu, Met, Phe, Pro, Trp, and Val was 0.733; the mean of all hydrophilic ones was 0.494. In summary, these findings proposed to determine classification quality in more detail by computing class- and residue-specific *p*-values (see Methods). Thus, the user can assess the statistical significance of each individual prediction. Table 4 lists the resulting performance for *p*-value cut-offs of 0.01, 0.025, and 0.05. As can be seen, specificity is high in all cases; sensitivity and precision are lower and class-dependent.

**Table 3:** Residue-specific MCC-values

Residue	*CAT_sites*	*LIG_sites*	*STRUC_sites*
A	-0.002	0.164	0.774

C	0.404	0.162	0.676

D	0.302	0.016	0.315

E	0.345	0.052	0.348

F	0.058	0.041	0.771

G	0.024	0.262	0.591

H	0.424	-0.063	0.086

I	-0.001	0.135	0.701

K	0.452	0.031	0.337

L	-0.001	0.056	0.815

M	-0.002	0.127	0.666

N	0.071	0.139	0.561

P	-	0.139	0.683

Q	0.098	0.111	0.678

R	0.287	0.040	0.319

S	0.307	0.156	0.595

T	0.055	0.174	0.682

V	-	0.119	0.761

W	-0.008	0.007	0.689

Y	0.097	0.046	0.741

The MCC-values were determined in a class- and residue-specific manner. Due to missing cases, MCC-values could not be determined for Pro and Val residues at *CAT_sites*.

**Table 4:** Performance of CLIPS-1D for different *p*-values

Cut-off	Sensitivity			Specificity			Precision		
	
	CAT	LIG	STRUC	CAT	LIG	STRUC	CAT	LIG	STRUC
0.010	0.170	0.030	0.225	0.996	0.991	0.991	0.316	0.176	0.827

0.025	0.276	0.077	0.445	0.992	0.977	0.977	0.270	0.178	0.789

0.050	0.401	0.137	0.582	0.987	0.954	0.961	0.246	0.165	0.742

The three performance measures were determined (see Methods) by selecting as positive cases all residue-positions with a *p*-value not greater than the given cut-off. Labels: "CAT" *CAT_sites*, "LIG" *LIG_sites*, "STRUC" *STRUC_sites*.

An alternative to CLIPS-1D is the algorithm ConSeq, which predicts functionally or structurally important residue-positions but does not distinguish catalytic and ligand-binding sites. Based on the analysis of five proteins, a success rate of 0.56 has been reported [5]. In order to estimate the performance of the latest ConSeq version [30], we have uploaded one sequence for each of the first five *ENZ *and *NO_ENZ *entries (see Additional file 1: Tables S3 and S4 for PDB-IDs) and used the Web server with default parameters. As ConSeq does not differentiate between catalytic sites and ligand-binding sites, the union of *CAT_sites *and *LIG_sites *was considered as positives in this case. For the combination of these residue-positions, sensitivity was 0.41, specificity 0.84, and precision 0.16; for *STRUC_sites *the values were 0.30, 0.86, and 0.31, respectively. A comparison of the performance values indicates that CLIPS-1D can compete with ConSeq.

### Utilizing CLIPS-1D as a web service

A version of CLIPS-1D trained on the full datasets is available as a Web service at http://www-bioinf.uni-regensburg.de/. Its usage requires to upload an MSA in multiple Fasta-format; the result will be sent to the user *via *email.

To illustrate the application of CLIPS-1D, we present an analysis of the enzyme indole-3-glycerol phosphate synthase (IGPS), which is found in many mesophilic and thermophilic species. IGPS belongs to the large and versatile family of (βα)_8_-barrel proteins, which is one of the oldest folds [31]. Additionally, folding kinetics [32] and 3D-structure of IGPS [33,34] have been studied in detail.

**Figure 2:**
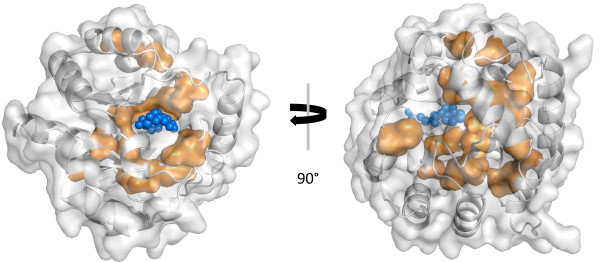
**Localization of *STRUC_sites *in sIGPS**. Based on PDB-ID 1A53, the surface of the whole protein (grey) and of residues predicted as *STRUC_sites *(orange) is shown. The substrate indole-3-glycerole phosphate is plotted in dark blue. The picture was generated by means of PyMOL [39].

We analyzed the HSSP-MSA related to PDB-ID 1A53, *i.e*. the IGPS from *Sulfolobus solfataricus*. Table 5 lists all CLIPS-1D predictions with a *p*-value ≤ 0.025. According to the respective PDB-sum page [35], E51, K53, K110, E159, N180, and S211 are the catalytic residues. Besides N180, which was predicted as *LIG_site*, the other 5 sites were correctly identified as *CAT_sites*. The sites which have contact to the ligand were classified as follows: *CAT_sites *E210, *LIG_sites *I232, *STRUC_sites *F112, L131, L231, *NOANN_sites *G212, G233, S234. Classified as *LIG_sites *were also K55, I179, and S181, which are all neighbors of catalytic sites. 20 residues were predicted as *STRUC_sites*; Figure 2 shows that all belong to the core of the protein. Their function will be discussed below.

**Table 5:** CLIPS-1D predictions for residue-positions in sIGPS (PDB-ID 1A53)

Residue	Position	*p_CAT_*	*p_LIG_*	*p_STRUC_*	*p_NOANN_*	*p*-value	Classification		
							
							CS	LBS	STRUC
I	49	0.001	0.154	0.824	0.022	0.003			SC

E	51	0.806	0.075	0.114	0.005	0.020	CAT		

K	53	0.835	0.065	0.088	0.012	0.004	CAT		

K	55	0.051	0.544	0.197	0.208	0.011		SC	

S	56	0.017	0.170	0.801	0.012	0.004			SC

L	60	0.002	0.128	0.829	0.041	0.019			IA

A	77	0.006	0.172	0.810	0.011	0.018			FC

I	82	0.002	0.259	0.667	0.073	0.011			SR

T	84	0.002	0.111	0.881	0.007	0.003			N

L	108	0.006	0.106	0.863	0.024	0.012			SR

K	110	0.866	0.078	0.046	0.011	0.002	CAT		

F	112	0.146	0.053	0.788	0.014	0.020		STRUC	FC

Q	118	0.007	0.114	0.872	0.008	0.002			FC

A	122	0.001	0.066	0.882	0.051	0.010			FC

A	127	0.024	0.193	0.776	0.008	0.022			N

L	131	0.001	0.071	0.920	0.008	0.006		STRUC	SR

L	132	0.004	0.164	0.794	0.038	0.023			SR,FC

I	133	0.005	0.169	0.790	0.036	0.005			FC

L	137	0.007	0.151	0.813	0.029	0.020			SC,FC

L	157	0.001	0.105	0.886	0.008	0.010			SC,FC

E	159	0.899	0.048	0.050	0.003	0.005	CAT		

D	165	0.189	0.071	0.699	0.040	0.007			N

I	179	0.001	0.819	0.068	0.112	0.021		SCE	

N	180	0.098	0.770	0.116	0.016	0.016	LIG		

S	181	0.011	0.774	0.134	0.081	0.019		SCE	

L	184	0.009	0.157	0.818	0.016	0.020			IA

L	197	0.003	0.130	0.818	0.049	0.020			N

E	210	0.866	0.059	0.068	0.007	0.008		CAT	

S	211	0.738	0.168	0.087	0.007	0.005	CAT		

L	231	0.003	0.224	0.762	0.011	0.025		STRUC	SC

I	232	0.006	0.835	0.059	0.099	0.017		LIG	

The first two columns give the residue and its position in sIGPS. The following four columns list the probabilities for the residue's membership with *CAT_sites*, *LIG_sites*, *STRUC_sites*, or *NOANN_sites*. The column labeled "*p*-value" lists the *p*-value for the class with max(*p_CLASS_*). The columns "CS" and "LBS" indicate the classification of known catalytic and ligand-binding sites. The last column lists the annotation deduced for residues predicted as *STRUC_sites*. Meaning of labels: "CAT", "LIG", "STRUC", residues predicted as *CAT_sites*, *LIG_sites*, or *STRUC_sites*, respectively. "SC" element of a stabilization center pair in sIGPS, "SCE" *ditto *in eIGPS, "SR" stabilization residue in sIGPS; see [36]. "FC" element of the folding core; see [37]. "IA" interaction with substrate; see [38]. "N" no function assigned.

### Strengths and weaknesses of CLIPS-1D

Adding the class *STRUC_sites *allowed us to compare properties of functionally and structurally important residue-positions and to assess their impact on classification quality.

For *CAT_sites*, the abundance scores indicate a strong bias of Arg, Asp, Glu, His, and Lys towards catalytic residue-positions, which is in agreement with previous findings [24]. *CAT_sites*, which were classified as structurally important, were most frequently Cys and Tyr residues. Both residues are not exceedingly overrepresented at catalytic sites and *abund*(*k, CAT_sites*)- and *abund*(*k, STRUC_sites*)-values are similarly high; compare Table 2. For extracellular proteins, structurally important Cys residues are frequently involved in disulphide bonds. Thus, algorithms like DISULFIND [40] can help to clarify CLIPS-1D's Cys classification.

Least specific was the classification of *LIG_sites*, which also suffered the most drastic loss of performance. The MCC-value dropped from 0.21 (gained with *SVM_LIG_*) to 0.12, and most misclassifications gave *STRUC_sites*, which is due to the similarity of these sites with respect to the features used for classification: For both classes, *cons_JSD_*(*k*) is most relevant for classification success, and among all combinations of abundance-values the pairs *abund*(*k, LIG_sites*) and *abund*(*k, STRUC_sites*) differ least; compare Table 2. The similarity of these residue-positions is further confirmed by the large number of *STRUC_sites *classified as functionally important by FRpred, which additionally suggests that the assessment of the predicted secondary structure and the predicted solvent accessibility contributes little to discriminate functionally and structurally important sites. It follows that *LIG_sites *and *STRUC_sites *span a fuzzy continuum, which cannot be divided by means of the considered sequence-based features. On the other hand, each MCC-value characterizes a binary classification and underestimates the performance of CLIPS-1D. For example, when assessing the performance of *LIG_sites via *an MCC-value, residue-positions classified as *STRUC_sites *were counted as false-negatives. A more detailed analysis of Figure 1 and the findings on sIGPS illustrate that *LIG_sites *were often classified as *CAT_sites *or *STRUC_sites *and not as sites without any function (*NOANN_sites*), which is a drastic difference not considered by an MCC-value.

For *STRUC_sites*, the MCC-value decreased from 0.78 to 0.67 for the above reasons; however, the MCC-value is still considerably high. Can one make plausible, why these buried residue-positions are preferentially occupied by a specific set of residues? At mean, hydrophobic interactions contribute 60% and hydrogen bonds 40% to protein stability; for the stability of larger proteins, hydrophobic interactions are even more important [41]. The fraction of misclassified hydrophobic *STRUC_sites *was low; compare MCC-values of Table 3. Thus, CLIPS-1D identifies with high reliability conserved residues of the protein's core, which are most likely important for protein stability. On the other hand, the analysis of *abund*(*k, STRUC_sites*)-values (compare Table 2) shows that not all *STRUC_sites *are conserved hydrophobic residues: The hydrophobic residues Ala, Ile, Met, and Val are underrepresented, whereas the hydrophilic residues Cys, Gly, and Tyr are overrepresented. Additionally, the comparison of abundance scores indicates a preference of Leu, Phe, and Pro for structurally relevant sites. These preferences reflect the specific function of these residues for secondary structure [42]. Additionally, the score-values demonstrate that CLIPS-1D does not exclusively select ILV-residues, which are considered important for protein folding [32]. *STRUC_sites*, misclassified as catalytic ones, were often Arg, Asp, and Glu, which shows that the *abund*(*k, CAT_sites*)-values have a strong effect on classification. *NOANN_sites *predicted as *CAT_sites *were frequently Arg, Asp, and His; Gly, Ser, and Thr were often predicted as *LIG_sites*. Most likely, at least some of these residue-positions belong to binding sites on the protein-surface *e.g*. protein-protein interfaces. Identifying these residues is possible [43], but beyond the scope of this study.

### *STRUC_sites *are crucial elements of the sIGPS structure

A detailed comparison of the two thermostable variants sIGPS from *S. solfataricus *[33], tIGPS from *Thermotoga maritima*, and the thermolabile eIGPS from *Escherichia coli *has made clear that these thermostable proteins have 7 strong salt bridges more than eIGPS, and that only 3 of 17 salt bridges in tIGPS and sIGPS are topologically conserved [44]. It follows that CLIPS-1D can only identify the specific subset of structurally important residue-positions which are relevant for most of the homologous proteins constituting the MSA under study. For sIGPS, tIGPS, and eIGPS stabilization centers (*SC*) and stabilization residues (*SR*) have been determined [36]. Residues of *SCs *form tight networks of cooperative interactions which are energetically stabilized; *SRs *are embedded into a conserved hydrophobic 3D-neigborhood. 20 residue-positions of sIGPS were classified as *STRUC_sites *by CLIPS-1D. 9 of these 20 residue-positions as well as the 3 false-positive *LIG_sites *are a *SC *or *SR *residue in one of the three homologous enzymes; compare Table 5. For sIGPS, the structure of folding cores, *i.e*. local substructures, which form early during protein folding has been determined by means of HD exchange experiments [37]. 8 of the *STRUC_sites *belong to fragments, which are strongest protected against deuterium exchange (> 84%, see Table 3 in reference [37]), which indicates their significant role in the partially folded protein. A molecular dynamics study [38] and a comparison of enzyme variants [34] have made clear that two more *STRUC_sites *belong to loops interacting with the substrate. When combining the above findings, only 4 of the 20 *STRUC_sites *have no accentuated function, which confirms the relevance of these sites for the enzyme's structure.

### Main application of CLIPS-1D: Predicting important sites of uncharacterized proteins

For the test cases of the CASP 7 contest, the *firestar *[17] and the I-TASSER [45] server have reached MCC-values of 0.7 when predicting functionally important residues; the performance of other servers has been substantially lower [17]. Both servers utilize the transfer of information from evolutionary related and well-characterized proteins. If applicable, this approach allows for a superior prediction quality. However, it fails completely if the function of homologous proteins is unknown. For such cases, methods are required that identify functionally and structurally important sites by analyzing conservation signals and propensity values. In contrast to ConSeq [5] and FrPred [8], CLIPS-1D predicts a specific role in catalysis, ligand-binding, or structure for each residue-position. The only prerequisite for its application is the existence of a sufficiently large number of homologous sequences, which can easily be combined to an MSA and which should be filtered according to our experience.

The number of genes which lack annotated homologs is huge: In mid 2011, the Pfam database [46] contained nearly 4000 domains of unknown function. Additionally, a comparison of databases for protein-coding genes and their products unravels a tremendous deficit of knowledge by indicating that function is unknown for more than 40% of all protein-coding genes [47]. These genes may code for unknown folds and novel enzymatic capabilities. However, if computational biology fails to identify function, an enormous battery of experiments have to be accomplished, due to the number of distinct enzymatic activities and other protein functions observed in Nature; see *e.g*. [48]. Therefore, all plausible hypotheses generated by CLIPS-1D and similar methods are of value and help to reduce the number of experimental analyses.

One might expect that exploiting the 3D-structure of a protein contributes a lot to functional assignment. This is not necessarily the case: Structure-based algorithms have failed to outperform MSA-based approaches in predicting catalytic sites and have maximally reached the same MCC-value; see [18] and references therein. However, if 3D-data and an MSA are at hand, features deduced from structure and from homologous sequences can be utilized in a concerted manner. In addition to the above features, signals caused by correlated mutations [3,49] can then be utilized to further characterize catalytic sites, which are surrounded by residues spanning a network of mutual information [50]. This is why we work on exploiting a combination of these features and the near future will show, whether this approach further improves classification quality. There is an urgent need for such methods: In mid 2011, no function has been attributed to more than 4% of the protein structures deposited in the Protein Data Bank [51].

## Conclusions

By analyzing an MSA by means of CLIPS-1D, residue-positions involved in catalysis can be identified with acceptable quality. In contrast, ligand-binding sites and residue-positions important for protein structure are hard to distinguish due to their similar patterns of conservation and residue propensities. Our MC-SVM can be applied to cases where the function of all homologs is unknown. The algorithm supports the user's decisions by computing a *p*-value for each prediction.

## Methods

### *CAT_sites *and *LIG_sites*, datasets of catalytic and ligand-binding residue-positions

To compile a test set of functionally important sites, we processed the content of the Catalytic Site Atlas (CSA) [52]. We exclusively utilized the manually curated entries of CSA and did not consider sites that have been annotated by means of PSI-BLAST alignments. In order to eliminate redundancy of proteins, we used the PISCES server [53] with a sequence-similarity cut-off of 25%. For each protein, an MSA was taken from the HSSP database [28] and selected for further analyses, if it contained at least 125 sequences. The resulting dataset consists of 264 enzymes and related MSAs, which we named *ENZ*. These proteins contain 840 catalytic residues, which we denominated *CAT_sites*. For these proteins we also deduced ligand-binding sites by exploiting PDBsum pages [35]. The resulting dataset consists of 216 proteins and contains 4466 binding sites, which we named *LIG_sites*. The datasets *CAT_sites *and *LIG_sites *do not overlap; their content is listed in Additional file 1: Tables S2 and S3.

In order to eliminate too similar and too distant sequences which might introduce a bias, the number of identical residues *ident*(*s_i_*, *s_j_*) was determined for each pair of sequences *s_i_*, *s_j _*belonging to the same MSA. Sequences were removed until the fraction of identical residues was in the range 0.25 ≤ *ident*(*s_i_*, *s_j_*) ≤ 0.90. Additionally, sequences deviating from the first one in length by more than 30% were deleted.

### *STRUC_sites*, a set of conserved residue-positions in proteins lacking enzymatic function

A set of 480 non-enzyme proteins has been compiled in reference [27]. Based on PDBsum and CSA, we re-annotated all entries and prepared a redundancy-free set of MSAs as explained above. The resulting dataset *NON_ENZ *consists of 136 proteins and related MSAs from HSSP with at least 50 sequences. In order to exclude residues from interfaces and other binding sites, we did not consider residue-positions lying at the protein surface by eliminating all sites with a relative solvent accessible surface area of at least 5% (see [43] and references therein). Among the remaining sites were 3703 with a conservation value *cons_ident _*(*k*) > 1.0 (see formula (2)). For lack of a more biochemically motivated classification scheme, these conserved sites were regarded as important for structure. We named this set *STRUC_sites*, its content is listed in Additional file 1: Table S4. We designated the complement *NO_ANN *sites; these are the remaining 19,223 residue-positions of the *NON_ENZ *dataset.

### Conservation of an individual site

An instructive measure to assess conservation of a single residue-position *k *is *max_frequ*(*k*), the largest amino acid frequency *f_k_*(*aa_i_*) observed in column *k *of an MSA:

(1) $$max\text{\_}frequ\left(k\right)\;=\;\underset{i=1..20}{\max}\left(f_{k}\left(aa_{i}\right)\right)$$

To normalize for MSA-specific variations of conservation, we computed *cons_ident _*(*k*), which is a z-score deduced from *max_frequ*(*k*) according to

(2) $$cons_{ident}\left(k\right)=\frac{max\text{\_}frequ\left(k\right)-\mu_{ident}}{\sigma_{ident}}$$

Mean μ*_ident _*and standard deviation σ*_ident _*values were determined individually for each MSA under study. An alternative conservation measure is the Jensen-Shannon divergence [8] of site *k*:

(3) $$JSD\left(k\right)=H\left(\frac{f_{K}^{obs}-f_{}^{backgr}}{2}\right)-\frac{1}{2}H\left(f_{K}^{obs}\right)-\frac{1}{2}H\left(f_{}^{backgr}\right)$$

$f_{K}^{obs}$ is the probability mass function for site *k *approximated as $f_{K}^{obs}\left(aa_{i}\right)=f_{k}\left(aa_{i}\right)$ by the amino acid frequencies observed in the respective column *k *of the MSA; the mean amino acid frequencies as found in the SwissProt database [54] were taken as background frequencies $f^{backgr}.H(.)$ is Shannon's entropy [55]. For classification, we used the z-score *cons_JSD _*(*k*):

(4) $$cons_{JSD}\left(k\right)=\frac{JSD\left(k\right)-\mu_{JSD}}{\sigma_{JSD}}$$

Mean μ*_JSD _*and standard deviation σ*_JSD _*values were determined individually for each MSA. For the prediction of functionally important residues, *JSD*(*k*) has performed better than other conservation measures [7].

### Conservation of a sequence neighborhood

To characterize the conservation of a sequence neighborhood, *cons_neib_*(*k*) was computed in analogy to [8]:

(5) $$cons_{neib}\left(k\right)=\frac{1}{\left|Neib\right|}\underset{l\;\in Neib}{\sum }w_{l}\;cons_{JSD}\left(k+l\right)$$

*Neib *= {-3,-2,-1,+1,+2,+3} determined the set of neighboring positions. The weights were: *w*_-1 _= *w*_+1 _= 3, *w*_-2 _= *w*_+2 _= 2, *w*_-3 _= *w*_+3 _= 1. Note that conservation of position *k *was not considered to compute *cons_neib_*(*k*).

### Propensities of catalytic sites, ligand-binding sites, and positions important for structure

Inspired by [24], three scores *abund*(*k, CLASS*) were computed as:

(6) $$abund\left(k,CLASS\right)=\overset{20}{\underset{i=1}{\sum }}f_{k}\left(aa_{i}\right)\log\frac{f^{CLASS}\left(aa_{i}\right)}{f_{}^{backgr}\left(aa_{i}\right)}$$

*f^backgr ^(aa_i_) *were the above background frequencies. *f^CLASS ^(aa_i_) *were the frequencies of residues from one set *CLASS *∈ {*CAT*_*sites*, *LIG*_*sites*, *STRUC*_*sites*}.

### Scoring propensities of a neighborhood

To assess the class-specific neighborhood of a site *k*, we introduced:

(7) $$abund_{neib}\left(aa_{s}^{k},CLASS\right)=\frac{1}{\left|Neib\right|}\overset{}{\underset{l\in Neib}{\sum }}\overset{20}{\underset{i=1}{\sum }}f_{k+l}\left(aa_{i}^{}\right)\log\frac{f_{k+l}^{CLASS}\left(aa_{i}|aa_{s}\right)}{f_{}^{backgr}\left(aa_{i}\right)}$$

Here, $aa_{s}^{k}$ is the amino acid *aa_s _*occurring at site *k *under consideration, *f_k+l _(aa_i_) *is the frequency of *aa_i _*at position *l *relative to *k *and $f_{k+l}^{CLASS}\left(aa_{i}|aa_{s}\right)$ is the conditional frequency of *aa_i _*at the same positional offset deduced from the neighborhood of all residues *aa_s _*of a set *CLASS *∈ {*CAT*_*sites*,*LIG*_*sites*,*STRUC*_*sites*}. *Neib *is the ± 3 neighborhood.

### Evaluating classification performance

To assess the performance of a classification, the rates *TPR *(*Sensitivity*), *FPR*, *Specificity*, and *Precision*

(8) $$TPR=\frac{TP}{TP+FN},FPR=\frac{FP}{FP+TN},Specificity=\frac{TN}{TN+FP},Precision=\frac{TP}{TP+FP}$$

as well as ROC and PROC curves were determined [56]. For a ROC curve, depending on a cut-off for one parameter (here it is *p_class _*(*k*)), the *TPR *values are plotted *versus *the *FPR *values. For a PROC curve, *Precision *is plotted *versus TPR*. As a further performance measure, the Matthews correlation coefficient (MCC) has been introduced [29]:

(9) $$MCC=\frac{TP\cdot \;TN-FP\;\cdot FN}{\sqrt{\left(TP+FN\right)\left(TP+FP\right)\left(TN+FP\right)\left(TN+FN\right)}}$$

MCC-values are considered a fair measure to assess performance on unbalanced sets of positives and negatives, as observed here [57]. In all formulae, *TP *is the number of true positives, *TN *the number of true negatives, *FP *the number of false positives and *FN *the number of false negatives. For example, when classifying catalytic sites with *SVM_CAT_*, positives are the selected *CAT_sites *and negatives are all other residue-positions of the considered MSAs.

### Classifying by means of support vector machines

We utilized the *libsvm *library [58] with a Gaussian radial basis function kernel and determined during training optimal parameters γ*_RBF _*and *C *by means of a grid search [59]. Prior to presenting features to the SVM, they were normalized according to

(10) $$V_{e}^{scaled}(k)=\frac{V_{e}(k)-\min(V_{e})}{\max(V_{e})-\min(V_{e})}$$

Here, *V_e_*(*k*) is for residue *k *the value of feature *e*, and min(*V_e_*) and max(*V_e_*) are the smallest and the largest value determined for this feature.

Our 2C-SVMs predict for each residue-position *k*, whether it is a catalytic site (*SVM_CAT_*), a ligand-binding site (*SVM_LIG_*), or a site important for structure (*SVM_STRUC_*). Taking *SVM_CAT _*as an example, an *a posteriori *probability *p_class _*(*k*), here it is *p_CAT _*(*k*), for the label "*k *is a catalytic site" was deduced from the distance of the feature set for *k *and the hyperplane separating catalytic and non-catalytic residue-positions [60].

We utilized *p_class _*(*k*) to assess performance and to assign classes. Training and assessment was organized as an 8-fold cross validation. For each training step, the number of positive and negative cases was balanced, *i.e*. for *SVM_CAT_*, residue-positions from *CAT_sites *and the same number of non-catalytic sites was selected. In order to eliminate sampling bias during the grid search, each parameter was deduced as means from training trials with the same positives and 50 different, randomly selected sets of negative cases. To compute the performance measures (*e.g*. MCC-values), all positive and all negative cases belonging to the selected subset of MSAs were classified.

Analogously, an MC-SVM was applied to the four classes *CAT_sites*, *LIG_sites*, *STRUC_sites*, and *NOANN_sites*. The output of the MC-SVM consists of four class-probabilities *p_class _*(see [60]) for each residue-position. These were deduced from the *a posteriori *probabilities of the six 2C-SVMs, which were trained on one specific combination of two classes, each. Each residue-positions *k *was assigned to the class, whose *p_class_*-value was largest. *p*-values were determined as follows: For each class and each residue, the respective cumulative distribution was deduced from the *p_class_*-values of all residue-positions *k *not belonging to the considered class. *I. e*., the *p*-value for a Glu-residue with *p_STRUC_*-value *s*(*k*) is the fraction of all Glu-residues from *NOANN_sites *reaching or surpassing *s*(*k*).

## Competing interests

The authors declare that they have no competing interests.

## Authors' contributions

JOJ designed and implemented algorithms, and trained and assessed the SVMs. MB, FK, and MP prepared datasets and were involved in programming and assessment. RM conceived of and coordinated the study, and wrote the manuscript. All authors read and approved the manuscript.

## Supplementary Material

Additional file 1 **A plot comparing *abund*(*k*, *CLASS*)-values, Figures and Tables giving performance-values of 2C-SVMs, and Tables listing the composition of datasets. (PDF 327 kb)**. Click here for file
