# Supplementary material for: CLIPS-1D: analysis of multiple sequence alignments to deduce for residue-positions a role in catalysis, ligand-binding, or protein structure
Source: BMC Bioinformatics. 2012 Apr 5;13:55. doi: 10.1186/1471-2105-13-55 (PMC3391178; doi:10.1186/1471-2105-13-55)
Supplement: Additional file 1 — A plot comparing abund(k, CLASS)-values, Figures and Tables giving performance-values of 2C-SVMs, and Tables listing the composition of datasets. (PDF 327 kb). [file 1471-2105-13-55-S1.PDF]

Supplementary Material

CLIPS-1D: Analysis of multiple sequence alignments to deduce for residue-positions a role in catalysis, ligand-binding, or protein structure

Jan-Oliver Janda[1], Markus Busch[1], Fabian Kück[2], Mikhail Porfenenko[1], Rainer Merkl[1$]

[1]Institute of Biophysics and Physical Biochemistry, University of Regensburg, 93040 Regensburg, Germany

[2]Faculty of Mathematics and Computer Science, University of Hagen, 58084 Hagen, Germany

[§]Corresponding author

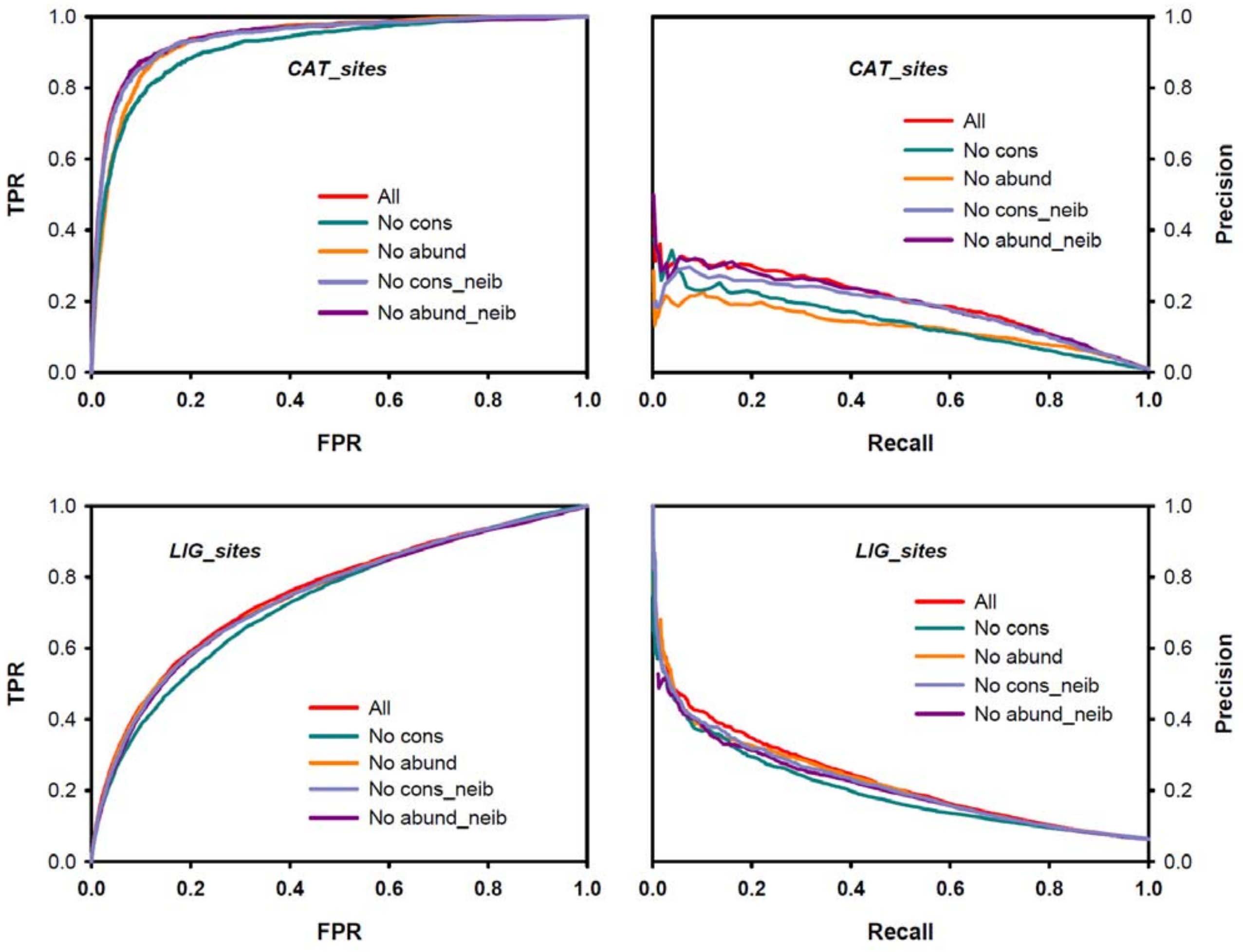

Figure S1: Performance of 2C-SVMs when classifying catalytic sites and ligand binding sites. The upper two panels are ROC-curves (left) and PROC-curves (right) for SVMs classifying all catalytic sites (*CAT_sites*). The lower two panels illustrate the performance for SVMs classifying ligand-binding sites (*LIG_sites*). For each classification task, 5 SVMs were compared which differ in the number of features to be analyzed. "All" gives the performance of an SVM utilizing all four features. The other graphs indicate the performance of an SVM using three features each. The legend names the feature, which was not used for classification: "No cons" for $cons_{JSD}(k)$, "No abund" stands for the $abund(k, CLASS)$-score, "No cons_neib" for $cons_{neib}(k)$, and "No abund_neib" for $abunb_{neib}(aa_s^k, CLASS)$.

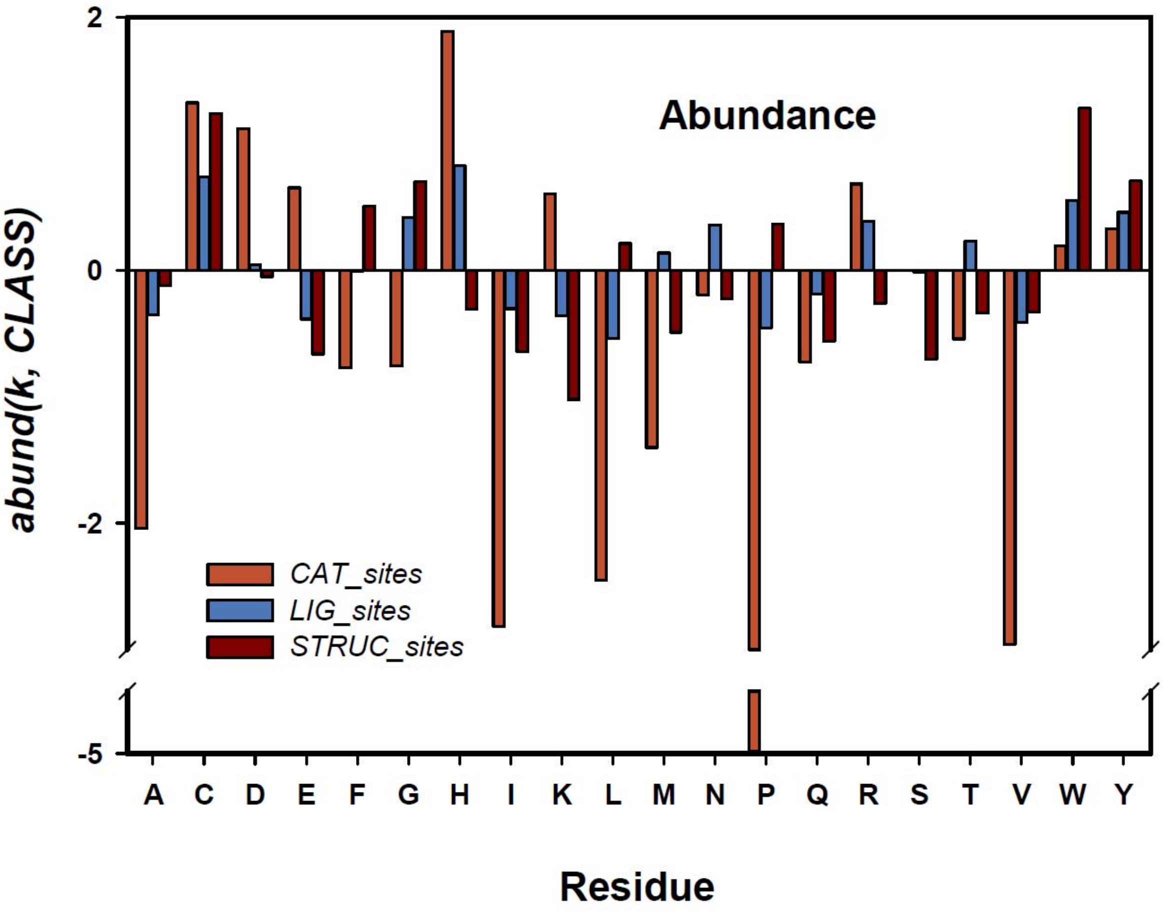

Figure S2: *abund*(*k, CLASS*) –values for amino acid residues. The scores were deduced from sites constituting a $CLASS \in \{CAT\_sites, LIG\_sites, STRUC\_sites\}$; see formula 6.

Features	*CAT_sites*	*LIG_sites*	*STRUC_sites*
All features	0.944/0.199<br>0.169/0.665/0.324	0.732/0.187<br>0.213/0.346/0.212	0.967/0.747<br>0.717/0.900/0.761
No cons	0.914/0.143<br>0.141/0.511/ 0.256	0.710/0.167<br>0.189/0.326/ 0.186	0.762/0.421<br>0.425/0.498/0.346
No abund	0.934/0.130<br>0.125/0.579/0.256	0.725/0.182<br>0.195/0.374/0.207	0.969/0.751<br>0.709/0.945/0.779
No cons_neib	0.941/0.180<br>0.187/0.581/0.319	0.725/0.179<br>0.191/0.363/0.201	0.969/0.751<br>0.734/0.919/0.783
No abund_neib	0.943/0.195<br>0.199/0.540/0.318	0.722/0.165<br>0.165/0.440/0.196	0.969/0.753<br>0.737/0.914/0.782
5 MSA<br>Sequence only	0.025/0.955/0.123<br>0.025/0.942/0.120	0.113/0.682/0.173<br>0.109/0.657/0.159	ND
6 MSA<br>Sequence only	0.035/0.936/0.153<br>0.034/0.910/0.147	0.135/0.585/0.192<br>0.131/0.564/0.180	ND
7 MSA<br>Sequence only	0.055/0.886/0.199<br>0.052/0.842/0.187	0.176/0.455/0.212<br>0.167/0.432/0.196	ND
8 MSA<br>Sequence only	0.076/0.818/0.231<br>0.071/0.768/0.215	0.212/0.365/0.219<br>0.200/0.345/0.202	ND
9 MSA<br>Sequence only	0.114/0.614/0.250<br>0.107/0.575/0.233	0.258/0.223/0.197<br>0.247/0.213/0.186	ND

Table S1: Classification performance of *CLIPS_1D* and FRpred on catalytic sites, ligand binding sites, and sites important for protein structure. The upper part lists performance values of SVM_{CAT}, SVM_{LIG}, and SVM_{STRUC}, when classifying the corresponding residue-positions. The lower part lists the performance, when using FRpred and the cutoff-values 5-9. The first column gives the configuration of the 2C-SVM or the cut-off. For the 2C-SVMs, the following configurations were assessed: "All features", the SVM utilized "*cons*" conservedness of residues (formula (4)), "*abund*" propensities for residues (formula (6)), "*cons_neib*" conservedness of the neighborhood (formula (5)), "*abund_neib*" propensities for

the neighborhood of residues (formula (7)). The performance values listed in rows below “All features” are due to 2C-SVMs utilizing a feature set lacking the one listed in column one. For 2C-SVMs, the first line of performance values are AUC values for ROC (AURC) and PROC (AUPC) curves. The second line gives the precision/recall/MCC values. For SVM_{STRUC}, the feature *abund_neib*(*k, STRUC_sites*) deteriorated prediction quality (compare data of “All features” and “No abund_neib”). Thus, the performance given for “No cons”, “No abund”, and “No cons_neib” was determined without this feature, *i.e.*, was deduced from an SVM utilizing two features each. For FRpred, precision/recall/MCC values are listed resulting from submitting to the program an MSA or an individual sequence.

For example, the final configuration of SVM_{CAT} reached on the set of catalytic residues an AURC of 0.944 and an AUPC of 0.199. Precision was 0.169, recall 0.665 and the MCC was 0.324. For the same dataset, when analyzing predictions with an FRpred score of 9, precision was 0.114, with a recall of 0.614 and an MCC of 0.250 for an analysis of downloaded HSSP-MSAs; the respective values were 0.107, 0.575, and 0.233 when we downloaded individual sequences. ND indicates that data were not determined.

Table S2

The dataset *CAT_sites*. For each site, PDB-ID, chain, amino acid, and position as deduced from the PDB-file are listed. Entries are separated by a ";".

135l, A, E, 35; 135l, A, D, 52; 13pk, A, R, 39; 13pk, A, K, 219; 13pk, A, G, 376; 13pk, A, G, 399; 1a05, A, Y, 140; 1a05, A, K, 190; 1a05, A, D, 222; 1a0j, A, H, 57; 1a0j, A, D, 102; 1a0j, A, S, 195; 1a4i, A, K, 56; 1a50, B, H, 86; 1a50, B, K, 87; 1a50, B, K, 167; 1a50, B, D, 305; 1a8h, A, K, 297; 1a8h, A, K, 300; 1af7, A, R, 98; 1af7, A, D, 154; 1aj0, A, N, 22; 1aj0, A, R, 63; 1aj0, A, R, 255; 1aj8, A, H, 223; 1aj8, A, H, 262; 1aj8, A, D, 312; 1ako, A, N, 7; 1ako, A, D, 151; 1ako, A, N, 153; 1ako, A, D, 229; 1ako, A, H, 259; 1ald, A, D, 33; 1ald, A, E, 187; 1ald, A, K, 229; 1alk, A, S, 102; 1alk, A, R, 166; 1aq0, A, E, 232; 1aq0, A, E, 280; 1aq0, A, K, 283; 1aq0, A, E, 288; 1ar1, A, E, 278; 1ar1, A, K, 354; 1arz, A, H, 159; 1arz, A, K, 163; 1ast, A, E, 93; 1ast, A, Y, 149; 1asy, A, R, 325; 1asy, A, D, 342; 1asy, A, R, 531; 1at1, A, R, 54; 1at1, A, T, 55; 1at1, A, R, 105; 1at1, A, H, 134; 1auo, A, L, 23; 1auo, A, S, 114; 1auo, A, Q, 115; 1auo, A, D, 168; 1auo, A, H, 199; 1auw, A, T, 159; 1auw, A, H, 160; 1auw, A, S, 281; 1auw, A, K, 287; 1auw, A, E, 294; 1ay4, A, W, 140; 1ay4, A, D, 222; 1ay4, A, K, 258; 1azw, A, S, 110; 1azw, A, D, 266; 1azw, A, H, 294; 1b5t, A, E, 28; 1b5t, A, D, 120; 1b66, A, C, 42; 1b66, A, D, 88; 1b66, A, H, 89; 1b66, A, E, 133; 1b6t, A, H, 18; 1b6t, A, R, 91; 1b6t, A, S, 129; 1b73, A, D, 7; 1b73, A, S, 8; 1b73, A, C, 70; 1b73, A, C, 178; 1b8g, A, Y, 145; 1b8g, A, D, 230; 1b8g, A, K, 273; 1b93, A, H, 19; 1b93, A, G, 66; 1b93, A, D, 71; 1b93, A, D, 91; 1b93, A, H, 98; 1b93, A, D, 101; 1b9h, A, F, 88; 1b9h, A, D, 159; 1b9h, A, K, 188; 1bd0, A, K, 39; 1bd0, A, R, 136; 1bd0, A, Y, 265; 1bd0, A, C, 311; 1bf2, A, D, 375; 1bf2, A, E, 435; 1bf2, A, D, 510; 1bg0, A, R, 126; 1bg0, A, E, 225; 1bg0, A, R, 229; 1bg0, A, R, 280; 1bg0, A, R, 309; 1bgl, A, E, 461; 1bgl, A, E, 537; 1bh2, A, E, 43; 1bh2, A, R, 178; 1bh2, A, T, 181; 1bh2, A, Q, 204; 1bmt, A, D, 757; 1bmt, A, H, 759; 1bmt, A, S, 810; 1bp2, A, G, 30; 1bp2, A, H, 48; 1bp2, A, D, 99; 1brw, A, H, 82; 1brw, A, R, 168; 1brw, A, S, 183; 1brw, A, K, 187; 1bs0, A, H, 133; 1bs0, A, E, 175; 1bs0, A, D, 204; 1bs0, A, K, 236; 1bs4, A, G, 45; 1bs4, A, Q, 50; 1bs4, A, L, 91; 1bs4, A, E, 133; 1bwz, A, C, 73; 1bwz, A, H, 159; 1bwz, A, E, 208; 1bwz, A, C, 217; 1bzc, A, D, 181; 1bzc, A, C, 215; 1bzc, A, R, 221; 1bzc, A, S, 222; 1c2t, A, N, 106; 1c2t, A, H, 108; 1c2t, A, S, 135; 1c2t, A, D, 144; 1c4t, A, T, 323; 1c4t, A, H, 375; 1c4z, A, R, 506; 1c4z, A, E, 539; 1c4z, A, E, 550; 1c4z, A, D, 607; 1c4z, A, H, 818; 1c4z, A, C, 820; 1cd5, A, D, 72; 1cd5, A, D, 141; 1cd5, A, H, 143; 1cd5, A, E, 148; 1cel, A, E, 212; 1cel, A, D, 214; 1cel, A, E, 217; 1cel, A, H, 228; 1cev, A, D, 126; 1cev, A, E, 271; 1cg6, A, D, 220; 1cg6, A, D, 222; 1cgk, A, C, 164; 1cgk, A, F, 215; 1cgk, A, H, 303; 1cgk, A, N, 336; 1chd, A, S, 164; 1chd, A, T, 165; 1chd, A, H, 190; 1chd, A, M, 283; 1chd, A, D, 286; 1ci8, A, S, 75; 1ci8, A, K, 78; 1ci8, A, Y, 181; 1ci8, A, W, 348; 1ci8, A, V, 351; 1cns, A, E, 67; 1cns, A, E, 89; 1cns, A, S, 120; 1cqj, A, E, 208; 1cqj, A, H, 246; 1cs1, A, R, 48; 1cs1, A, Y, 101; 1cs1, A, D, 173; 1cw0, A, H, 69; 1cw0, A, D, 97; 1cwy, A, D, 293; 1cwy, A, E, 340; 1cwy, A, D, 395; 1d0s, A, E, 317; 1d1q, A, A, 13; 1d1q, A, R, 19; 1d1q, A, S, 20; 1d1q, A, D, 132; 1d2r, A, K, 192; 1d2r, A, K, 195; 1d6o, A, D, 37; 1d6o, A, I, 56; 1d6o, A, Y, 82; 1d8t, A, D, 21; 1daa, A, K, 145; 1daa, A, E, 177; 1daa, A, L, 201; 1dae, A, T, 11; 1dae, A, K, 15; 1dae, A, K, 37; 1dae, A, S, 41; 1dbt, A, D, 60; 1dbt, A, K, 62; 1dci, A, E, 196; 1dci, A, D, 204; 1dco, A, H, 62; 1dco, A, H, 63; 1dco, A, H, 80; 1dco, A, D, 89; 1dd8, A, C, 163; 1dd8, A, H, 298; 1dd8, A, K, 328; 1dd8, A, H, 333; 1dd8, A, F, 390; 1dd8, A, F, 392; 1dgs, A, K, 116; 1dgs, A, D, 118; 1dgs, A, R, 196; 1dgs, A, K, 312; 1dhp, A, Y, 133; 1dhp, A, R, 138; 1dhp, A, K, 161; 1dj0, A, D, 60; 1dj1, A, A, 48; 1dj1, A, H, 52; 1dj1, A, W, 191; 1djl, A, Y, 890; 1djl, A, R, 925; 1djl, A, Y, 1006; 1djo, A, T, 1020; 1djo, A, Y, 1034; 1djo, A, T, 1100; 1djo, A, E, 1294; 1dnp, A, W, 306; 1dnp, A, W, 359; 1dnp, A, W, 382; 1do8, A, Y, 112; 1do8, A, K, 183; 1do8, A, D, 278; 1dpg, A, D, 177; 1dpg, A, H, 240; 1dqa, A, E, 559; 1dqa,

A, K, 691; 1dqa, A, D, 767; 1dqa, A, H, 866; 1dqr, A, K, 210; 1dqr, A, E, 216; 1dqr, A, G, 271; 1dqr, A, R, 272; 1dqr, A, E, 357; 1dqr, A, H, 388; 1dqr, A, K, 518; 1dup, A, D, 90; 1dup, A, D, 92; 1dxe, A, H, 50; 1dxe, A, R, 75; 1dzr, A, H, 63; 1dzr, A, D, 170; 1e2a, A, H, 78; 1e2a, A, Q, 80; 1e2a, A, D, 81; 1e2a, A, H, 82; 1e7q, A, A, 107; 1e7q, A, C, 109; 1e7q, A, Y, 136; 1e7q, A, K, 140; 1e7q, A, H, 179; 1ebf, A, D, 219; 1ebf, A, K, 223; 1ecx, A, H, 99; 1ecx, A, D, 177; 1ecx, A, K, 203; 1ef8, A, H, 66; 1ef8, A, G, 110; 1ef8, A, Y, 140; 1eg7, A, K, 1074; 1emd, A, D, 150; 1emd, A, H, 177; 1eug, A, D, 64; 1eug, A, H, 187; 1ex1, A, D, 285; 1ex1, A, E, 491; 1eyi, A, D, 68; 1eyi, A, D, 74; 1eyi, A, E, 98; 1f2d, A, K, 51; 1f2d, A, S, 78; 1f2d, A, T, 81; 1f2d, A, Y, 295; 1f2d, A, E, 296; 1f2v, A, H, 43; 1f6d, A, D, 95; 1f6d, A, E, 117; 1f6d, A, E, 131; 1f6d, A, H, 213; 1f75, A, R, 33; 1f75, A, R, 42; 1f75, A, R, 197; 1f75, A, R, 203; 1f7l, A, K, 62; 1f7l, A, H, 105; 1f7u, A, K, 156; 1f7u, A, H, 159; 1f7u, A, H, 162; 1f8m, A, H, 180; 1f8m, A, C, 191; 1f8m, A, R, 228; 1fgh, A, D, 100; 1fgh, A, H, 101; 1fgh, A, H, 147; 1fgh, A, D, 165; 1fgh, A, H, 167; 1fgh, A, E, 262; 1fgh, A, S, 642; 1fiq, A, E, 1261; 1fnb, A, S, 96; 1fnb, A, C, 272; 1fnb, A, E, 312; 1fps, A, R, 126; 1fps, A, F, 253; 1fq0, A, E, 45; 1fq0, A, K, 133; 1fro, A, E, 172; 1fua, A, E, 73; 1fua, A, Y, 113; 1fuq, A, T, 187; 1fuq, A, H, 188; 1fuq, A, K, 324; 1fuq, A, E, 331; 1fva, A, C, 72; 1fva, A, Y, 103; 1fva, A, E, 115; 1fva, A, Y, 155; 1fva, A, C, 218; 1fva, A, C, 227; 1fwk, A, T, 183; 1g64, A, K, 41; 1g64, A, T, 43; 1g6t, A, K, 22; 1g6t, A, D, 313; 1g6t, A, E, 341; 1g6t, A, H, 385; 1g6t, A, K, 411; 1g72, A, D, 297; 1g79, A, R, 197; 1g8f, A, R, 290; 1g8p, A, R, 289; 1g99, A, S, 10; 1g99, A, R, 91; 1g99, A, D, 148; 1g99, A, G, 212; 1g99, A, R, 241; 1gal, A, E, 412; 1gal, A, H, 516; 1gal, A, H, 559; 1get, A, C, 42; 1get, A, C, 47; 1get, A, H, 439; 1get, A, E, 444; 1gim, A, D, 13; 1gim, A, H, 41; 1gim, A, Q, 224; 1gp5, A, K, 213; 1gpa, A, K, 568; 1gpa, A, R, 569; 1gpa, A, K, 574; 1gpa, A, T, 676; 1gpr, A, T, 66; 1gpr, A, H, 68; 1gpr, A, H, 83; 1gpr, A, G, 85; 1gq8, A, Q, 113; 1gq8, A, Q, 135; 1gq8, A, D, 136; 1gq8, A, D, 157; 1gsa, A, K, 160; 1gsa, A, R, 210; 1gsa, A, R, 225; 1gtp, A, H, 112; 1gtp, A, H, 179; 1gxs, A, S, 158; 1gxs, A, W, 270; 1h4g, A, E, 94; 1h4g, A, E, 184; 1h7o, A, D, 131; 1h7o, A, S, 179; 1h7o, A, K, 210; 1h7o, A, K, 263; 1hdh, A, R, 55; 1hdh, A, K, 113; 1hdh, A, H, 115; 1hdh, A, H, 211; 1hdh, A, D, 317; 1hdh, A, K, 375; 1hfs, A, E, 202; 1hfs, A, M, 219; 1hka, A, R, 82; 1hka, A, R, 92; 1hrd, A, K, 125; 1hrd, A, D, 165; 1hrk, A, H, 263; 1hrk, A, H, 341; 1hrk, A, E, 343; 1hti, A, N, 11; 1hti, A, K, 13; 1hti, A, H, 95; 1hti, A, E, 165; 1hti, A, G, 171; 1hv9, A, R, 18; 1i6p, A, D, 44; 1i6p, A, R, 46; 1i8d, A, F, 2; 1i8d, A, S, 41; 1i8d, A, C, 48; 1i8d, A, M, 64; 1i8d, A, H, 102; 1im5, A, D, 10; 1im5, A, K, 94; 1im5, A, A, 129; 1im5, A, C, 133; 1ima, A, E, 70; 1ima, A, T, 95; 1iph, A, H, 128; 1iph, A, S, 167; 1iph, A, N, 201; 1ir3, A, D, 1132; 1ir3, A, R, 1136; 1itx, A, D, 200; 1itx, A, E, 204; 1j00, A, G, 44; 1j00, A, N, 73; 1j00, A, D, 154; 1j00, A, H, 157; 1j09, A, K, 246; 1j53, A, E, 14; 1j53, A, E, 61; 1j53, A, H, 162; 1j79, A, D, 250; 1j7g, A, Q, 78; 1j7g, A, F, 79; 1j7g, A, T, 80; 1jhf, A, M, 118; 1jhf, A, S, 119; 1jhf, A, D, 127; 1jhf, A, E, 152; 1jhf, A, K, 156; 1js4, A, D, 55; 1js4, A, D, 58; 1js4, A, E, 424; 1kae, A, E, 326; 1kae, A, H, 327; 1kaz, A, E, 71; 1kc7, A, H, 455; 1kc7, A, C, 831; 1kra, A, H, 219; 1kra, A, D, 221; 1kra, A, H, 320; 1kra, A, R, 336; 1ksj, A, Q, 70; 1kyw, A, H, 269; 1l1r, A, R, 63; 1l1r, A, E, 100; 1l6p, A, Y, 42; 1l6p, A, D, 68; 1l6p, A, F, 70; 1l6p, A, Y, 71; 1l6p, A, C, 103; 1l6p, A, C, 109; 1l7n, A, D, 11; 1l7n, A, F, 12; 1l7n, A, D, 13; 1l7n, A, G, 100; 1l7n, A, K, 144; 1l7n, A, D, 171; 1lam, A, D, 255; 1lam, A, K, 262; 1lam, A, R, 336; 1ldm, A, D, 166; 1ldm, A, R, 169; 1ldm, A, H, 193; 1lj1, A, A, 402; 1ljl, A, C, 10; 1ljl, A, G, 12; 1ljl, A, N, 13; 1ljl, A, S, 14; 1ljl, A, R, 16; 1ljl, A, S, 17; 1ljl, A, C, 82; 1ljl, A, C, 89; 1ljl, A, D, 105; 1lxa, A, H, 125; 1m21, A, K, 123; 1m21, A, S, 202; 1m21, A, S, 226; 1m9c, A, R, 55; 1m9c, A, F, 60; 1m9c, A, Q, 63; 1m9c, A, N, 102; 1m9c, A, F, 113; 1m9c, A, L, 122; 1mbb, A, R, 159; 1mbb, A, S, 229; 1mbb, A, E, 325; 1mht, A, C, 81; 1mht, A, E, 119; 1mj9, A, S, 304; 1mj9, A, E, 338; 1mka, A, H, 70; 1mka, A, V, 76; 1mka, A, G, 79; 1mka, A, C, 80; 1mka, A, D, 84; 1mla, A, S, 92; 1mla, A, H, 201; 1mla, A, Q, 250; 1moq, A, E, 481; 1moq, A, K, 485; 1moq, A, E, 488; 1moq, A, K, 603; 1mrq, A, D, 50; 1mrq, A, Y, 55; 1mrq, A, K, 84; 1mrq, A, H, 117; 1mud, A, E, 37; 1mud, A, N, 138; 1mvn, A, H, 90; 1mvn, A, S, 175;

1myr, A, R, 95; 1myr, A, Q, 187; 1myr, A, S, 190; 1myr, A, N, 328; 1myr, A, Y, 330; 1myr, A, E, 409; 1n2c, A, C, 62; 1n2c, A, A, 65; 1n2c, A, R, 96; 1n2c, A, C, 154; 1n2c, A, L, 158; 1n2c, A, H, 195; 1nbf, A, N, 218; 1nbf, A, C, 223; 1nbf, A, H, 464; 1nbf, A, D, 481; 1ndh, A, Y, 65; 1ni4, A, H, 263; 1nsp, A, K, 16; 1nsp, A, N, 119; 1nvt, A, D, 102; 1nzy, A, F, 64; 1nzy, A, H, 90; 1nzy, A, G, 114; 1nzy, A, W, 137; 1nzy, A, D, 145; 1o04, A, K, 192; 1o04, A, E, 268; 1o04, A, S, 302; 1o04, A, E, 399; 1o98, A, S, 62; 1o98, A, D, 154; 1o98, A, R, 261; 1oas, A, K, 41; 1oas, A, S, 272; 1oh9, A, K, 8; 1oh9, A, G, 11; 1oh9, A, G, 45; 1oh9, A, K, 217; 1oj4, A, K, 10; 1oj4, A, D, 141; 1onr, A, D, 17; 1onr, A, E, 96; 1onr, A, K, 132; 1os7, A, R, 270; 1p1x, A, D, 102; 1p1x, A, K, 167; 1p1x, A, K, 201; 1pjb, A, K, 74; 1pjb, A, H, 95; 1pjb, A, E, 117; 1pjb, A, D, 269; 1pmi, A, Q, 111; 1pmi, A, E, 294; 1pmi, A, R, 304; 1pud, A, D, 102; 1q18, A, D, 100; 1q3q, A, D, 64; 1q3q, A, T, 97; 1q3q, A, T, 98; 1q3q, A, D, 393; 1qcn, A, H, 133; 1qcn, A, E, 199; 1qcn, A, R, 237; 1qcn, A, Q, 240; 1qcn, A, K, 253; 1qcn, A, E, 364; 1qf6, A, R, 363; 1qh5, A, D, 58; 1qhf, A, H, 8; 1qhf, A, R, 59; 1qhf, A, E, 86; 1qhf, A, H, 181; 1qlh, A, S, 48; 1qlh, A, H, 51; 1qpr, A, R, 105; 1qpr, A, K, 140; 1qpr, A, E, 201; 1qpr, A, D, 222; 1qtn, A, R, 258; 1qtn, A, H, 317; 1qtn, A, G, 350; 1qtn, A, C, 360; 1qum, A, E, 261; 1qz9, A, F, 129; 1qz9, A, D, 201; 1qz9, A, K, 227; 1ra2, A, I, 5; 1ra2, A, M, 20; 1ra2, A, D, 27; 1ra2, A, L, 28; 1ra2, A, F, 31; 1ra2, A, L, 54; 1ra2, A, I, 94; 1rdd, A, H, 124; 1req, A, Y, 89; 1req, A, H, 244; 1req, A, K, 604; 1req, A, D, 608; 1req, A, H, 610; 1rhs, A, R, 186; 1rhs, A, R, 248; 1rhs, A, K, 249; 1rhs, A, G, 250; 1rhs, A, V, 251; 1rhs, A, T, 252; 1s3i, A, H, 106; 1s3i, A, D, 142; 1s95, A, D, 274; 1s95, A, R, 275; 1s95, A, N, 303; 1s95, A, H, 304; 1s95, A, R, 400; 1s95, A, H, 427; 1snn, A, D, 30; 1snn, A, C, 55; 1snn, A, Y, 95; 1snn, A, N, 106; 1snn, A, S, 147; 1snn, A, E, 185; 1t4c, A, Q, 17; 1t4c, A, E, 140; 1t4c, A, D, 169; 1t4c, A, G, 260; 1t4c, A, G, 261; 1tdj, A, K, 62; 1tdj, A, S, 315; 1tmo, A, S, 149; 1trk, A, H, 30; 1trk, A, H, 263; 1tys, A, E, 58; 1tys, A, Y, 94; 1tys, A, S, 146; 1tys, A, R, 166; 1tys, A, D, 169; 1uae, A, N, 23; 1uae, A, C, 115; 1uae, A, D, 305; 1uae, A, R, 397; 1uaq, A, E, 64; 1uaq, A, S, 89; 1uaq, A, C, 91; 1uas, A, D, 130; 1uas, A, D, 185; 1ujn, A, R, 109; 1ujn, A, K, 131; 1ujn, A, K, 210; 1ujn, A, E, 220; 1ujn, A, R, 224; 1ujn, A, N, 228; 1ujn, A, H, 235; 1un1, A, E, 85; 1un1, A, D, 87; 1un1, A, E, 89; 1uok, A, D, 199; 1uok, A, E, 255; 1uok, A, D, 329; 1uqr, A, N, 11; 1uqr, A, R, 18; 1uqr, A, Y, 23; 1uqr, A, E, 98; 1uqr, A, H, 100; 1uqr, A, R, 107; 1uqt, A, H, 154; 1uqt, A, D, 361; 1uro, A, D, 86; 1uro, A, Y, 164; 1vr7, A, S, 55; 1vr7, A, S, 63; 1vr7, A, H, 68; 1vr7, A, C, 83; 1xgm, A, E, 187; 1xny, A, G, 182; 1xny, A, G, 183; 1xny, A, G, 419; 1xny, A, A, 420; 1xqd, A, S, 286; 1xqd, A, D, 393; 1xyz, A, E, 645; 1xyz, A, H, 723; 1xyz, A, E, 754; 1xyz, A, D, 756; 1ycf, A, H, 25; 1ycf, A, Y, 195; 1ze1, A, D, 39; 1ze1, A, Y, 67; 1zio, A, K, 13; 1zio, A, R, 127; 1zio, A, R, 160; 1zio, A, D, 162; 1zio, A, D, 163; 1zio, A, R, 171; 1zoi, A, W, 31; 1zoi, A, S, 97; 1zoi, A, T, 98; 1zoi, A, A, 122; 1zoi, A, D, 227; 1zoi, A, H, 256; 1zym, A, T, 168; 1zym, A, H, 189; 2abk, A, K, 120; 2abk, A, D, 138; 2ace, A, S, 200; 2ace, A, E, 327; 2ace, A, H, 440; 2apr, A, D, 35; 2apr, A, S, 38; 2apr, A, D, 218; 2apr, A, T, 221; 2bif, A, R, 255; 2bif, A, A, 256; 2bif, A, N, 262; 2bif, A, R, 305; 2bif, A, E, 325; 2bif, A, H, 390; 2cpu, A, D, 197; 2cpu, A, E, 233; 2cpu, A, N, 300; 2dhn, A, E, 22; 2dhn, A, K, 100; 2dln, A, E, 15; 2dln, A, S, 150; 2dln, A, Y, 216; 2dln, A, R, 255; 2dln, A, G, 276; 2hdh, A, S, 137; 2hdh, A, H, 158; 2hdh, A, E, 170; 2hdh, A, N, 208; 2hsa, A, H, 185; 2hsa, A, H, 188; 2hsa, A, Y, 190; 2jcw, A, H, 63; 2jcw, A, R, 143; 2npx, A, H, 10; 2npx, A, S, 41; 2npx, A, R, 303; 2oat, A, F, 177; 2oat, A, D, 263; 2oat, A, K, 292; 2pda, A, T, 31; 2pda, A, E, 64; 2pda, A, R, 114; 2pda, A, N, 996; 2pfl, A, W, 333; 2pfl, A, C, 418; 2pfl, A, C, 419; 2pfl, A, G, 734; 2pgd, A, G, 130; 2pgd, A, K, 183; 2pgd, A, N, 187; 2pgd, A, E, 190; 2pia, A, N, 44; 2pia, A, S, 58; 2pia, A, C, 199; 2pia, A, E, 223; 2pth, A, N, 10; 2pth, A, H, 20; 2pth, A, D, 93; 2tdt, A, D, 141; 2tdt, A, G, 166; 2tdt, A, E, 169; 2tps, A, R, 59; 2tps, A, S, 130; 2tps, A, K, 159; 2ypn, A, D, 84; 2ypn, A, R, 131; 2ypn, A, R, 132; 2ypn, A, R, 149; 2ypn, A, R, 155; 2ypn, A, C, 242; 3mdd, A, E, 376; 3r1r, A, C, 225; 3r1r, A, N, 437; 3r1r, A, C, 439; 3r1r, A, E, 441; 3r1r, A, C, 462; 4mdh, A, S, 1; 4mdh, A, D, 158; 4mdh, A, H, 186; 5cpa, A, R, 71; 5cpa, A, R, 127; 5cpa, A, E, 270; 5eat, A, R, 264; 5eat, A,

W, 273; 5eat, A, T, 401; 5eat, A, T, 402; 5eat, A, T, 403; 5eat, A, R, 441; 5eat, A, D, 444; 5eat, A, Y, 520; 5eat, A, D, 525; 5eat, A, Y, 527; 5enl, A, E, 168; 5enl, A, E, 211; 5enl, A, K, 345; 5enl, A, H, 373; 5fit, A, Q, 83; 5fit, A, H, 94; 5fit, A, H, 96; 5rsa, A, H, 12; 5rsa, A, K, 41; 5rsa, A, H, 119; 7atj, A, R, 38; 7atj, A, H, 42; 7atj, A, N, 70;

Table S3

The dataset *LIG_sites*. For each site, PDB-ID, chain, amino acid, and position as deduced from the PDB-file are listed. Entries are separated by a ";".

13pk, A, D, 24; 13pk, A, N, 26; 13pk, A, H, 62; 13pk, A, G, 64; 13pk, A, R, 65; 13pk, A, L, 103; 13pk, A, R, 105; 13pk, A, R, 135; 13pk, A, G, 168; 13pk, A, T, 169; 13pk, A, R, 172; 13pk, A, G, 217; 13pk, A, A, 218; 13pk, A, K, 223; 13pk, A, G, 241; 13pk, A, A, 242; 13pk, A, Y, 245; 13pk, A, F, 294; 13pk, A, A, 314; 13pk, A, L, 315; 13pk, A, N, 338; 13pk, A, P, 340; 13pk, A, G, 342; 13pk, A, V, 343; 13pk, A, F, 344; 13pk, A, E, 345; 13pk, A, V, 347; 13pk, A, G, 374; 13pk, A, G, 375; 13pk, A, D, 377; 13pk, A, S, 378; 13pk, A, L, 384; 1a05, A, E, 88; 1a05, A, R, 95; 1a05, A, R, 105; 1a05, A, R, 133; 1a05, A, V, 193; 1a05, A, D, 246; 1a05, A, D, 250; 1a0j, A, H, 40; 1a0j, A, E, 70; 1a0j, A, N, 72; 1a0j, A, V, 75; 1a0j, A, E, 77; 1a0j, A, E, 80; 1a0j, A, P, 152; 1a0j, A, D, 153; 1a0j, A, T, 154; 1a0j, A, R, 156; 1a0j, A, D, 189; 1a0j, A, S, 190; 1a0j, A, C, 191; 1a0j, A, Q, 192; 1a0j, A, G, 193; 1a0j, A, W, 215; 1a0j, A, G, 216; 1a0j, A, G, 219; 1a0j, A, C, 220; 1a0j, A, G, 226; 1a4i, A, T, 148; 1a4i, A, V, 171; 1a4i, A, G, 172; 1a4i, A, R, 173; 1a4i, A, S, 174; 1a4i, A, V, 177; 1a4i, A, H, 196; 1a4i, A, S, 197; 1a4i, A, L, 202; 1a4i, A, A, 215; 1a4i, A, T, 216; 1a4i, A, G, 217; 1a4i, A, Q, 218; 1a4i, A, M, 221; 1a4i, A, C, 236; 1a4i, A, G, 237; 1a4i, A, I, 238; 1a4i, A, G, 276; 1a4i, A, T, 279; 1a50, B, A, 85; 1a50, B, Q, 114; 1a50, B, T, 190; 1a50, B, C, 230; 1a50, B, V, 231; 1a50, B, G, 232; 1a50, B, G, 233; 1a50, B, G, 234; 1a50, B, S, 235; 1a50, B, N, 236; 1a50, B, P, 270; 1a50, B, G, 303; 1a50, B, F, 306; 1a50, B, S, 308; 1a50, B, A, 348; 1a50, B, E, 350; 1a50, B, S, 377; 1a50, B, G, 378; 1a8h, A, C, 127; 1a8h, A, C, 130; 1a8h, A, C, 144; 1a8h, A, I, 146; 1a8h, A, H, 147; 1af7, A, A, 38; 1af7, A, T, 90; 1af7, A, T, 91; 1af7, A, N, 92; 1af7, A, L, 93; 1af7, A, T, 94; 1af7, A, A, 123; 1af7, A, S, 125; 1af7, A, E, 129; 1af7, A, I, 155; 1af7, A, D, 156; 1af7, A, V, 211; 1af7, A, N, 212; 1af7, A, L, 213; 1af7, A, R, 230; 1af7, A, N, 231; 1af7, A, V, 232; 1af7, A, F, 236; 1aj0, A, I, 20; 1aj0, A, T, 62; 1aj0, A, D, 96; 1aj0, A, N, 115; 1aj0, A, I, 117; 1aj0, A, M, 139; 1aj0, A, D, 185; 1aj0, A, F, 190; 1aj0, A, L, 215; 1aj0, A, G, 217; 1aj0, A, S, 219; 1aj0, A, R, 220; 1aj0, A, K, 221; 1aj0, A, H, 257; 1aj8, A, H, 188; 1aj8, A, N, 191; 1aj8, A, I, 222; 1aj8, A, G, 224; 1aj8, A, A, 226; 1aj8, A, K, 254; 1aj8, A, K, 256; 1aj8, A, I, 257; 1aj8, A, M, 258; 1aj8, A, G, 259; 1aj8, A, A, 260; 1aj8, A, G, 261; 1aj8, A, R, 263; 1aj8, A, R, 271; 1aj8, A, K, 305; 1aj8, A, I, 307; 1aj8, A, N, 310; 1aj8, A, V, 311; 1aj8, A, F, 333; 1aj8, A, R, 337; 1aj8, A, R, 353; 1aj8, A, R, 356; 1alk, A, D, 51; 1alk, A, D, 101; 1alk, A, T, 155; 1alk, A, E, 322; 1alk, A, D, 327; 1alk, A, H, 331; 1alk, A, D, 369; 1alk, A, H, 370; 1alk, A, H, 372; 1alk, A, H, 412; 1aq0, A, L, 188; 1aq0, A, N, 190; 1aq0, A, Y, 201; 1aq0, A, R, 249; 1aq0, A, F, 250; 1aq0, A, H, 254; 1ar1, A, I, 33; 1ar1, A, L, 36; 1ar1, A, F, 37; 1ar1, A, T, 50; 1ar1, A, M, 53; 1ar1, A, R, 54; 1ar1, A, M, 55; 1ar1, A, E, 56; 1ar1, A, Q, 58; 1ar1, A, H, 59; 1ar1, A, P, 60; 1ar1, A, G, 61; 1ar1, A, Q, 63; 1ar1, A, Y, 64; 1ar1, A, W, 87; 1ar1, A, I, 91; 1ar1, A, H, 94; 1ar1, A, G, 95; 1ar1, A, M, 98; 1ar1, A, M, 99; 1ar1, A, V, 102; 1ar1, A, V, 103; 1ar1, A, G, 163; 1ar1, A, W, 164; 1ar1, A, W, 272; 1ar1, A, H, 276; 1ar1, A, V, 279; 1ar1, A, Y, 280; 1ar1, A, H, 325; 1ar1, A, H, 326; 1ar1, A, L, 342; 1ar1, A, T, 344; 1ar1, A, I, 347; 1ar1, A, A, 348; 1ar1, A, T, 351; 1ar1, A, G, 352; 1ar1, A, I, 359; 1ar1, A, W, 363; 1ar1, A, F, 369; 1ar1, A, G, 387; 1ar1, A, V, 388; 1ar1, A, G, 390; 1ar1, A, V, 391; 1ar1, A, L, 393; 1ar1, A, S, 394; 1ar1, A, D, 399; 1ar1, A, H, 403; 1ar1, A, D, 404; 1ar1, A, Y, 406; 1ar1, A, V, 408; 1ar1, A, H, 411; 1ar1, A, F, 412; 1ar1, A, H, 413; 1ar1, A, V, 415; 1ar1, A, M, 416; 1ar1, A, S, 417; 1ar1, A, S, 436; 1ar1, A, W, 443; 1ar1, A, Q, 446; 1ar1, A, F, 460; 1ar1, A, F, 461; 1ar1, A, H, 464; 1ar1, A, F, 465; 1ar1, A, R, 468; 1ar1, A, R, 473; 1ar1, A, R, 474; 1ar1, A, Y, 475; 1ar1, A, D, 477; 1ar1, A, Y, 494; 1ar1, A, S, 496; 1ar1, A, F, 500; 1ar1, A, F, 503; 1ar1, A, G, 505; 1ar1, A, I, 506; 1ar1, A, L, 511; 1arz, A, H, 160; 1arz, A, V, 217; 1arz, A, R, 240; 1ast, A, H, 92; 1ast, A, H, 96; 1ast, A, H, 102; 1asy, A, N,

117; 1asy, A, R, 119; 1asy, A, Q, 120; 1asy, A, Q, 121; 1asy, A, L, 125; 1asy, A, F, 127; 1asy, A, Q, 138; 1asy, A, L, 140; 1asy, A, K, 142; 1asy, A, K, 155; 1asy, A, E, 177; 1asy, A, P, 178; 1asy, A, I, 179; 1asy, A, K, 180; 1asy, A, S, 181; 1asy, A, E, 188; 1asy, A, E, 202; 1asy, A, P, 205; 1asy, A, L, 207; 1asy, A, D, 210; 1asy, A, A, 221; 1asy, A, L, 223; 1asy, A, P, 224; 1asy, A, V, 225; 1asy, A, V, 226; 1asy, A, N, 227; 1asy, A, L, 228; 1asy, A, D, 229; 1asy, A, T, 230; 1asy, A, E, 327; 1asy, A, N, 328; 1asy, A, S, 329; 1asy, A, N, 330; 1asy, A, T, 331; 1asy, A, H, 332; 1asy, A, R, 333; 1asy, A, H, 334; 1asy, A, M, 335; 1asy, A, F, 338; 1asy, A, D, 421; 1asy, A, S, 423; 1asy, A, T, 424; 1asy, A, E, 478; 1asy, A, I, 479; 1asy, A, L, 480; 1asy, A, S, 481; 1asy, A, G, 526; 1asy, A, I, 542; 1asy, A, K, 553; 1at1, A, S, 52; 1at1, A, T, 53; 1at1, A, Q, 137; 1at1, A, R, 167; 1at1, A, T, 168; 1at1, A, R, 229; 1at1, A, Q, 231; 1at1, A, P, 266; 1at1, A, L, 267; 1ay4, A, Y, 70; 1ay4, A, G, 107; 1ay4, A, G, 108; 1ay4, A, T, 109; 1ay4, A, N, 194; 1ay4, A, A, 224; 1ay4, A, Y, 225; 1ay4, A, S, 255; 1ay4, A, S, 257; 1ay4, A, R, 266; 1b5t, A, T, 59; 1b5t, A, Y, 60; 1b5t, A, H, 88; 1b5t, A, T, 90; 1b5t, A, C, 91; 1b5t, A, I, 92; 1b5t, A, L, 117; 1b5t, A, R, 118; 1b5t, A, G, 119; 1b5t, A, M, 130; 1b5t, A, Y, 131; 1b5t, A, A, 132; 1b5t, A, A, 150; 1b5t, A, Y, 152; 1b5t, A, H, 156; 1b5t, A, E, 158; 1b5t, A, A, 159; 1b5t, A, D, 165; 1b5t, A, N, 168; 1b5t, A, R, 171; 1b5t, A, K, 172; 1b5t, A, I, 181; 1b5t, A, Q, 183; 1b5t, A, Y, 275; 1b66, A, H, 23; 1b66, A, L, 25; 1b66, A, H, 48; 1b66, A, H, 50; 1b66, A, T, 105; 1b66, A, T, 106; 1b66, A, E, 107; 1b66, A, N, 137; 1b6t, A, H, 104; 1b6t, A, R, 107; 1b6t, A, S, 121; 1b6t, A, K, 122; 1b8g, A, G, 119; 1b8g, A, A, 120; 1b8g, A, T, 121; 1b8g, A, N, 124; 1b8g, A, N, 202; 1b8g, A, I, 232; 1b8g, A, Y, 233; 1b8g, A, S, 270; 1b8g, A, S, 272; 1b8g, A, R, 281; 1b93, A, L, 16; 1b93, A, V, 17; 1b93, A, A, 18; 1b93, A, K, 23; 1b93, A, L, 26; 1b93, A, T, 45; 1b93, A, G, 46; 1b93, A, T, 48; 1b93, A, S, 65; 1b93, A, F, 88; 1b9h, A, N, 61; 1b9h, A, G, 62; 1b9h, A, T, 63; 1b9h, A, L, 66; 1b9h, A, T, 87; 1b9h, A, S, 90; 1b9h, A, S, 91; 1b9h, A, A, 161; 1b9h, A, H, 162; 1b9h, A, S, 183; 1b9h, A, Q, 185; 1b9h, A, G, 195; 1bd0, A, V, 37; 1bd0, A, Y, 43; 1bd0, A, L, 85; 1bd0, A, H, 166; 1bd0, A, N, 203; 1bd0, A, S, 204; 1bd0, A, R, 219; 1bd0, A, G, 221; 1bd0, A, I, 222; 1bd0, A, Y, 284; 1bd0, A, M, 312; 1bd0, A, D, 313; 1bd0, A, Y, 354; 1bf2, A, D, 128; 1bf2, A, E, 229; 1bf2, A, T, 230; 1bf2, A, N, 232; 1bf2, A, D, 259; 1bg0, A, S, 63; 1bg0, A, G, 64; 1bg0, A, V, 65; 1bg0, A, G, 66; 1bg0, A, Y, 68; 1bg0, A, G, 112; 1bg0, A, L, 113; 1bg0, A, D, 114; 1bg0, A, P, 115; 1bg0, A, S, 122; 1bg0, A, R, 124; 1bg0, A, H, 185; 1bg0, A, F, 194; 1bg0, A, W, 221; 1bg0, A, M, 233; 1bg0, A, G, 237; 1bg0, A, D, 238; 1bg0, A, C, 271; 1bg0, A, T, 273; 1bg0, A, N, 274; 1bg0, A, S, 282; 1bg0, A, V, 283; 1bg0, A, H, 284; 1bg0, A, T, 311; 1bg0, A, R, 312; 1bg0, A, G, 313; 1bg0, A, E, 314; 1bg0, A, H, 315; 1bg0, A, D, 324; 1bgl, A, D, 15; 1bgl, A, N, 18; 1bgl, A, V, 21; 1bgl, A, Y, 161; 1bgl, A, Q, 163; 1bgl, A, D, 193; 1bgl, A, D, 201; 1bgl, A, E, 416; 1bgl, A, H, 418; 1bh2, A, A, 41; 1bh2, A, G, 42; 1bh2, A, S, 44; 1bh2, A, G, 45; 1bh2, A, K, 46; 1bh2, A, S, 47; 1bh2, A, T, 48; 1bh2, A, D, 150; 1bh2, A, S, 151; 1bh2, A, L, 175; 1bh2, A, R, 176; 1bh2, A, T, 177; 1bh2, A, K, 180; 1bh2, A, D, 200; 1bh2, A, G, 202; 1bh2, A, G, 203; 1bh2, A, N, 269; 1bh2, A, K, 270; 1bh2, A, D, 272; 1bh2, A, L, 273; 1bh2, A, C, 325; 1bh2, A, S, 326; 1bh2, A, T, 327; 1bmt, A, E, 694; 1bmt, A, M, 698; 1bmt, A, M, 701; 1bmt, A, V, 704; 1bmt, A, G, 705; 1bmt, A, L, 715; 1bmt, A, V, 718; 1bmt, A, A, 722; 1bmt, A, M, 725; 1bmt, A, I, 751; 1bmt, A, G, 756; 1bmt, A, V, 758; 1bmt, A, D, 760; 1bmt, A, I, 761; 1bmt, A, G, 762; 1bmt, A, I, 765; 1bmt, A, V, 766; 1bmt, A, G, 802; 1bmt, A, L, 803; 1bmt, A, S, 804; 1bmt, A, L, 806; 1bmt, A, I, 807; 1bmt, A, T, 808; 1bmt, A, L, 831; 1bmt, A, G, 833; 1bmt, A, G, 834; 1bmt, A, A, 835; 1bmt, A, T, 836; 1bmt, A, V, 857; 1bmt, A, Q, 858; 1bmt, A, N, 859; 1bmt, A, A, 860; 1bmt, A, T, 863; 1bmt, A, T, 889; 1bp2, A, F, 5; 1bp2, A, L, 19; 1bp2, A, F, 22; 1bp2, A, Y, 28; 1bp2, A, G, 32; 1bp2, A, C, 44; 1bp2, A, T, 47; 1bp2, A, D, 49; 1bp2, A, C, 98; 1bp2, A, N, 101; 1bp2, A, A, 102; 1bp2, A, F, 106; 1brw, A, Y, 46; 1brw, A, F, 47; 1brw, A, R, 48; 1brw, A, G, 49; 1brw, A, K, 81; 1brw, A, S, 83; 1brw, A, G, 88; 1brw, A, T, 90; 1brw, A, T, 92; 1brw, A, K, 108; 1brw, A, S, 110; 1brw, A, T, 120; 1brw, A, R, 216; 1brw, A, L, 243; 1brw, A, A, 246; 1brw, A, E, 255; 1bs0, A, R, 22; 1bs0, A, R, 23; 1bs0, A, S, 107; 1bs0, A, G, 108; 1bs0, A, F, 109; 1bs0, A, R, 130;

1bs0, A, L, 147; 1bs0, A, R, 148; 1bs0, A, R, 149; 1bs4, A, E, 41; 1bs4, A, E, 42; 1bs4, A, G, 43; 1bs4, A, I, 44; 1bs4, A, I, 86; 1bs4, A, E, 88; 1bs4, A, G, 89; 1bs4, A, C, 90; 1bs4, A, P, 94; 1bs4, A, E, 95; 1bs4, A, R, 97; 1bs4, A, H, 132; 1bs4, A, H, 136; 1bzc, A, Y, 46; 1bzc, A, R, 47; 1bzc, A, D, 48; 1bzc, A, K, 120; 1bzc, A, F, 182; 1bzc, A, S, 216; 1bzc, A, A, 217; 1bzc, A, G, 218; 1bzc, A, I, 219; 1bzc, A, G, 220; 1bzc, A, Q, 262; 1c2t, A, N, 10; 1c2t, A, G, 11; 1c2t, A, S, 12; 1c2t, A, N, 13; 1c2t, A, R, 64; 1c2t, A, G, 87; 1c2t, A, F, 88; 1c2t, A, M, 89; 1c2t, A, R, 90; 1c2t, A, I, 91; 1c2t, A, L, 92; 1c2t, A, V, 97; 1c2t, A, I, 107; 1c2t, A, P, 109; 1c2t, A, L, 118; 1c2t, A, V, 139; 1c2t, A, T, 140; 1c2t, A, D, 141; 1c2t, A, E, 142; 1c2t, A, L, 143; 1c2t, A, Q, 170; 1c2t, A, E, 173; 1c4t, A, E, 250; 1c4t, A, L, 278; 1c4t, A, L, 377; 1c4t, A, D, 379; 1c4t, A, G, 380; 1c4t, A, E, 382; 1cel, A, Q, 175; 1cel, A, R, 251; 1cel, A, P, 258; 1cel, A, D, 259; 1cel, A, N, 270; 1cel, A, F, 273; 1cel, A, T, 281; 1cel, A, E, 295; 1cel, A, P, 314; 1cel, A, E, 325; 1cel, A, W, 376; 1cel, A, Y, 381; 1cev, A, H, 99; 1cev, A, D, 122; 1cev, A, H, 124; 1cev, A, D, 226; 1cev, A, D, 228; 1cg6, A, G, 17; 1cg6, A, T, 18; 1cg6, A, R, 60; 1cg6, A, H, 61; 1cg6, A, P, 69; 1cg6, A, T, 93; 1cg6, A, A, 94; 1cg6, A, C, 95; 1cg6, A, G, 96; 1cg6, A, F, 177; 1cg6, A, I, 194; 1cg6, A, N, 195; 1cg6, A, M, 196; 1cg6, A, T, 197; 1cg6, A, T, 219; 1cg6, A, V, 236; 1cgk, A, T, 132; 1cgk, A, S, 133; 1cgk, A, E, 192; 1cgk, A, V, 193; 1cgk, A, T, 194; 1cgk, A, T, 197; 1cgk, A, G, 216; 1cgk, A, D, 217; 1cgk, A, I, 254; 1cgk, A, G, 256; 1cgk, A, L, 263; 1cgk, A, T, 264; 1cgk, A, F, 265; 1cgk, A, S, 338; 1cgk, A, P, 375; 1ci8, A, H, 45; 1ci8, A, Y, 133; 1ci8, A, L, 135; 1ci8, A, D, 150; 1ci8, A, I, 152; 1ci8, A, A, 275; 1ci8, A, R, 292; 1ci8, A, S, 355; 1ci8, A, W, 356; 1ci8, A, F, 357; 1ci8, A, V, 366; 1ci8, A, L, 368; 1ci8, A, T, 382; 1cqj, A, G, 14; 1cqj, A, T, 16; 1cqj, A, G, 17; 1cqj, A, S, 18; 1cqj, A, Q, 19; 1cqj, A, V, 38; 1cqj, A, P, 40; 1cqj, A, K, 42; 1cqj, A, Y, 71; 1cqj, A, V, 72; 1cqj, A, P, 73; 1cqj, A, F, 76; 1cqj, A, S, 80; 1cqj, A, I, 95; 1cqj, A, T, 96; 1cqj, A, E, 97; 1cqj, A, N, 122; 1cqj, A, C, 123; 1cqj, A, P, 124; 1cqj, A, I, 136; 1cqj, A, S, 153; 1cqj, A, G, 154; 1cqj, A, T, 155; 1cw0, A, A, 2; 1cw0, A, D, 3; 1cw0, A, V, 4; 1cw0, A, H, 5; 1cw0, A, R, 10; 1cw0, A, S, 11; 1cw0, A, K, 12; 1cw0, A, N, 13; 1cw0, A, M, 14; 1cw0, A, R, 15; 1cw0, A, A, 16; 1cw0, A, I, 17; 1cw0, A, A, 18; 1cw0, A, T, 19; 1cw0, A, T, 22; 1cw0, A, A, 23; 1cw0, A, I, 24; 1cw0, A, G, 48; 1cw0, A, R, 49; 1cw0, A, D, 51; 1cw0, A, T, 63; 1cw0, A, G, 65; 1cw0, A, C, 66; 1cw0, A, F, 67; 1cw0, A, W, 68; 1cw0, A, H, 71; 1cw0, A, C, 73; 1cw0, A, Y, 74; 1cw0, A, K, 77; 1cw0, A, P, 79; 1cw0, A, A, 80; 1cw0, A, T, 81; 1cw0, A, R, 82; 1cw0, A, W, 86; 1cw0, A, K, 89; 1cw0, A, N, 93; 1cw0, A, R, 96; 1cw0, A, R, 99; 1cw0, A, E, 116; 1cw0, A, C, 117; 1cw0, A, G, 121; 1cw0, A, R, 122; 1cw0, A, K, 124; 1d0s, A, A, 78; 1d0s, A, S, 80; 1d0s, A, V, 84; 1d0s, A, Q, 88; 1d0s, A, L, 175; 1d0s, A, M, 177; 1d0s, A, A, 178; 1d0s, A, N, 179; 1d0s, A, T, 180; 1d0s, A, G, 202; 1d0s, A, A, 203; 1d0s, A, L, 315; 1d1q, A, L, 14; 1d1q, A, G, 15; 1d1q, A, N, 16; 1d1q, A, F, 17; 1d1q, A, C, 18; 1d1q, A, H, 52; 1d1q, A, W, 134; 1d6o, A, G, 83; 1d6o, A, A, 84; 1d6o, A, T, 85; 1d6o, A, G, 86; 1d8t, A, E, 3; 1d8t, A, E, 6; 1d8t, A, H, 19; 1d8t, A, H, 22; 1d8t, A, G, 23; 1d8t, A, K, 24; 1d8t, A, T, 25; 1d8t, A, T, 26; 1d8t, A, Y, 39; 1d8t, A, R, 44; 1d8t, A, F, 46; 1d8t, A, D, 50; 1d8t, A, P, 53; 1d8t, A, S, 65; 1d8t, A, H, 66; 1d8t, A, P, 82; 1d8t, A, H, 84; 1d8t, A, A, 85; 1d8t, A, V, 88; 1d8t, A, M, 98; 1d8t, A, D, 109; 1d8t, A, P, 111; 1d8t, A, M, 112; 1d8t, A, P, 113; 1d8t, A, R, 116; 1d8t, A, R, 123; 1d8t, A, Q, 124; 1d8t, A, V, 125; 1d8t, A, V, 127; 1d8t, A, Y, 129; 1d8t, A, N, 135; 1d8t, A, K, 136; 1d8t, A, D, 138; 1d8t, A, M, 139; 1d8t, A, V, 140; 1d8t, A, D, 141; 1d8t, A, D, 142; 1d8t, A, E, 143; 1d8t, A, L, 146; 1d8t, A, E, 152; 1d8t, A, R, 154; 1d8t, A, E, 155; 1d8t, A, Q, 159; 1d8t, A, D, 165; 1d8t, A, T, 167; 1d8t, A, P, 168; 1d8t, A, I, 169; 1d8t, A, V, 170; 1d8t, A, R, 171; 1d8t, A, S, 173; 1d8t, A, A, 174; 1d8t, A, L, 175; 1d8t, A, W, 184; 1d8t, A, K, 187; 1d8t, A, L, 189; 1d8t, A, E, 190; 1d8t, A, A, 192; 1d8t, A, G, 193; 1d8t, A, D, 196; 1d8t, A, S, 197; 1d8t, A, Y, 198; 1d8t, A, E, 215; 1d8t, A, D, 216; 1d8t, A, F, 218; 1d8t, A, S, 219; 1d8t, A, I, 220; 1d8t, A, S, 221; 1d8t, A, G, 222; 1d8t, A, R, 223; 1d8t, A, V, 226; 1d8t, A, T, 228; 1d8t, A, G, 229; 1d8t, A, R, 230; 1d8t, A, I, 247; 1d8t, A, T, 256; 1d8t, A, G, 257; 1d8t, A, V, 258; 1d8t, A, E, 259; 1d8t, A, M, 260; 1d8t, A, F, 261; 1d8t, A, R, 262; 1d8t, A, L, 264; 1d8t, A, N, 273; 1d8t, A, V, 274; 1d8t, A, G, 275;

1d8t, A, L, 277; 1d8t, A, E, 287; 1d8t, A, H, 301; 1d8t, A, T, 302; 1d8t, A, Y, 309; 1d8t, A, G, 353; 1d8t, A, N, 355; 1d8t, A, L, 362; 1d8t, A, I, 363; 1d8t, A, D, 370; 1d8t, A, R, 373; 1d8t, A, A, 389; 1d8t, A, V, 391; 1d8t, A, L, 392; 1d8t, A, G, 393; 1daa, A, Y, 31; 1daa, A, R, 50; 1daa, A, R, 138; 1daa, A, S, 179; 1daa, A, S, 180; 1daa, A, S, 181; 1daa, A, N, 182; 1daa, A, G, 203; 1daa, A, I, 204; 1daa, A, T, 205; 1daa, A, T, 239; 1daa, A, S, 240; 1daa, A, T, 241; 1dae, A, A, 40; 1dae, A, P, 79; 1dae, A, A, 117; 1dae, A, G, 118; 1dbt, A, D, 11; 1dbt, A, K, 33; 1dbt, A, D, 65; 1dbt, A, I, 66; 1dbt, A, T, 69; 1dbt, A, L, 122; 1dbt, A, T, 123; 1dbt, A, V, 160; 1dbt, A, P, 182; 1dbt, A, R, 185; 1dbt, A, Q, 194; 1dbt, A, V, 212; 1dbt, A, G, 214; 1dbt, A, R, 215; 1dci, A, R, 82; 1dci, A, R, 86; 1dci, A, I, 126; 1dci, A, D, 182; 1dci, A, Q, 193; 1dci, A, K, 195; 1dci, A, V, 205; 1dci, A, G, 216; 1dci, A, R, 218; 1dci, A, N, 222; 1dci, A, K, 230; 1dci, A, L, 278; 1dci, A, I, 279; 1dci, A, L, 301; 1dgs, A, L, 85; 1dgs, A, E, 114; 1dgs, A, H, 115; 1dgs, A, V, 117; 1dgs, A, E, 169; 1dgs, A, Y, 221; 1dgs, A, H, 253; 1dgs, A, K, 288; 1dgs, A, C, 406; 1dgs, A, C, 409; 1dgs, A, C, 422; 1dgs, A, C, 427; 1dhp, A, A, 152; 1dhp, A, V, 154; 1dhp, A, K, 155; 1dhp, A, I, 157; 1dj0, A, H, 202; 1dj0, A, V, 204; 1dj0, A, R, 205; 1dj1, A, P, 44; 1dj1, A, V, 47; 1dj1, A, W, 51; 1dj1, A, P, 145; 1dj1, A, D, 146; 1dj1, A, A, 147; 1dj1, A, F, 158; 1dj1, A, L, 171; 1dj1, A, M, 172; 1dj1, A, A, 174; 1dj1, A, H, 175; 1dj1, A, L, 177; 1dj1, A, G, 178; 1dj1, A, K, 179; 1dj1, A, T, 180; 1dj1, A, H, 181; 1dj1, A, N, 184; 1dj1, A, S, 185; 1dj1, A, L, 232; 1dj1, A, T, 234; 1djl, A, G, 889; 1djl, A, G, 891; 1djl, A, A, 895; 1djl, A, H, 920; 1djl, A, V, 922; 1djl, A, A, 923; 1djl, A, G, 924; 1djl, A, M, 926; 1djl, A, P, 927; 1djl, A, G, 964; 1djl, A, A, 965; 1djl, A, N, 966; 1djl, A, D, 967; 1djl, A, T, 968; 1djl, A, I, 981; 1djl, A, M, 984; 1djl, A, P, 985; 1djl, A, V, 986; 1djl, A, K, 999; 1djl, A, R, 1000; 1djl, A, S, 1001; 1djl, A, G, 1003; 1djl, A, V, 1004; 1djl, A, G, 1005; 1djl, A, G, 1024; 1djl, A, D, 1025; 1djl, A, A, 1026; 1djo, A, G, 1019; 1djo, A, A, 1066; 1djo, A, S, 1067; 1djo, A, E, 1068; 1djo, A, G, 1099; 1djo, A, D, 1101; 1djo, A, S, 1125; 1dnp, A, H, 44; 1dnp, A, E, 106; 1dnp, A, V, 107; 1dnp, A, N, 108; 1dnp, A, E, 109; 1dnp, A, Y, 222; 1dnp, A, T, 234; 1dnp, A, S, 235; 1dnp, A, R, 236; 1dnp, A, L, 237; 1dnp, A, S, 238; 1dnp, A, W, 271; 1dnp, A, E, 274; 1dnp, A, L, 275; 1dnp, A, W, 277; 1dnp, A, R, 278; 1dnp, A, Y, 281; 1dnp, A, C, 292; 1dnp, A, K, 293; 1dnp, A, R, 295; 1dnp, A, W, 338; 1dnp, A, N, 341; 1dnp, A, R, 344; 1dnp, A, M, 345; 1dnp, A, A, 348; 1dnp, A, L, 370; 1dnp, A, D, 372; 1dnp, A, G, 373; 1dnp, A, D, 374; 1dnp, A, A, 377; 1dnp, A, N, 378; 1dnp, A, G, 381; 1do8, A, H, 154; 1do8, A, K, 156; 1do8, A, R, 165; 1do8, A, L, 167; 1do8, A, G, 168; 1do8, A, G, 192; 1do8, A, I, 193; 1do8, A, R, 194; 1do8, A, R, 197; 1do8, A, D, 244; 1do8, A, R, 245; 1do8, A, G, 247; 1do8, A, E, 255; 1do8, A, D, 256; 1do8, A, N, 259; 1do8, A, D, 279; 1do8, A, T, 283; 1do8, A, L, 310; 1do8, A, G, 311; 1do8, A, A, 312; 1do8, A, G, 313; 1do8, A, E, 314; 1do8, A, A, 315; 1do8, A, D, 345; 1do8, A, K, 346; 1do8, A, V, 392; 1do8, A, A, 393; 1do8, A, G, 394; 1do8, A, A, 395; 1do8, A, L, 398; 1do8, A, L, 419; 1do8, A, S, 420; 1do8, A, N, 421; 1do8, A, G, 446; 1do8, A, G, 465; 1do8, A, N, 466; 1do8, A, N, 467; 1do8, A, I, 479; 1do8, A, L, 480; 1do8, A, N, 482; 1do8, A, R, 542; 1do8, A, Y, 552; 1do8, A, R, 556; 1dpg, A, E, 147; 1dpg, A, K, 148; 1dpg, A, I, 176; 1dpg, A, H, 178; 1dpg, A, Y, 179; 1dpg, A, K, 182; 1dpg, A, K, 343; 1dpg, A, Y, 415; 1dqa, A, Y, 479; 1dqa, A, E, 528; 1dqa, A, T, 558; 1dqa, A, C, 561; 1dqa, A, L, 562; 1dqa, A, A, 564; 1dqa, A, S, 565; 1dqa, A, N, 567; 1dqa, A, R, 568; 1dqa, A, R, 571; 1dqa, A, R, 590; 1dqa, A, S, 626; 1dqa, A, R, 627; 1dqa, A, F, 628; 1dqa, A, S, 651; 1dqa, A, G, 652; 1dqa, A, D, 653; 1dqa, A, A, 654; 1dqa, A, M, 655; 1dqa, A, G, 656; 1dqa, A, M, 657; 1dqa, A, N, 658; 1dqa, A, M, 659; 1dqa, A, S, 661; 1dqa, A, S, 684; 1dqa, A, N, 686; 1dqa, A, D, 690; 1dqa, A, K, 692; 1dqa, A, V, 720; 1dqa, A, K, 722; 1dqa, A, K, 735; 1dqa, A, A, 751; 1dqa, A, H, 752; 1dqa, A, N, 755; 1dqa, A, G, 806; 1dqa, A, G, 807; 1dqa, A, A, 826; 1dqa, A, S, 852; 1dqa, A, L, 853; 1dqa, A, A, 856; 1dqa, A, L, 862; 1dqa, A, S, 865; 1dqa, A, H, 869; 1dqa, A, N, 870; 1dqr, A, I, 156; 1dqr, A, G, 157; 1dqr, A, G, 158; 1dqr, A, S, 159; 1dqr, A, A, 208; 1dqr, A, S, 209; 1dqr, A, T, 211; 1dqr, A, T, 214; 1dqr, A, Q, 353; 1dqr, A, Q, 511; 1dxe, A, W, 24; 1dxe, A, Q, 151; 1dxe, A, E, 153; 1dxe, A, F, 174; 1dxe, A, G, 176; 1dxe, A, P, 177; 1dxe, A, D, 179; 1dxe, A, L, 216; 1dzr, A,

M, 1; 1dzr, A, M, 2; 1dzr, A, P, 17; 1dzr, A, K, 18; 1dzr, A, G, 21; 1dzr, A, D, 22; 1dzr, A, G, 25; 1dzr, A, E, 29; 1dzr, A, S, 30; 1dzr, A, Y, 31; 1dzr, A, N, 32; 1dzr, A, F, 36; 1dzr, A, R, 60; 1dzr, A, K, 73; 1dzr, A, W, 99; 1dzr, A, G, 101; 1dzr, A, N, 103; 1dzr, A, R, 110; 1dzr, A, Q, 111; 1dzr, A, H, 120; 1dzr, A, Y, 133; 1dzr, A, S, 167; 1dzr, A, K, 169; 1e7q, A, R, 5; 1e7q, A, G, 10; 1e7q, A, H, 11; 1e7q, A, R, 12; 1e7q, A, G, 13; 1e7q, A, M, 14; 1e7q, A, V, 15; 1e7q, A, S, 17; 1e7q, A, R, 20; 1e7q, A, R, 21; 1e7q, A, V, 32; 1e7q, A, R, 34; 1e7q, A, T, 35; 1e7q, A, R, 36; 1e7q, A, L, 39; 1e7q, A, N, 40; 1e7q, A, L, 41; 1e7q, A, L, 42; 1e7q, A, E, 54; 1e7q, A, A, 62; 1e7q, A, A, 63; 1e7q, A, A, 64; 1e7q, A, V, 66; 1e7q, A, G, 67; 1e7q, A, G, 68; 1e7q, A, I, 69; 1e7q, A, V, 70; 1e7q, A, A, 71; 1e7q, A, I, 86; 1e7q, A, L, 105; 1e7q, A, G, 106; 1e7q, A, S, 108; 1e7q, A, R, 152; 1e7q, A, P, 163; 1e7q, A, T, 164; 1e7q, A, N, 165; 1e7q, A, L, 166; 1e7q, A, H, 170; 1e7q, A, N, 177; 1e7q, A, S, 178; 1e7q, A, D, 198; 1e7q, A, P, 238; 1e7q, A, M, 239; 1e7q, A, R, 269; 1e7q, A, V, 271; 1e7q, A, W, 311; 1ebf, A, I, 11; 1ebf, A, G, 12; 1ebf, A, A, 13; 1ebf, A, G, 14; 1ebf, A, V, 15; 1ebf, A, V, 16; 1ebf, A, E, 40; 1ebf, A, A, 41; 1ebf, A, N, 92; 1ebf, A, T, 93; 1ebf, A, S, 94; 1ebf, A, P, 115; 1ebf, A, N, 116; 1ebf, A, K, 117; 1ebf, A, E, 143; 1ebf, A, V, 146; 1ebf, A, G, 147; 1ebf, A, A, 148; 1ebf, A, L, 150; 1ebf, A, A, 339; 1ebf, A, G, 340; 1ebf, A, T, 344; 1ecx, A, N, 8; 1ecx, A, A, 9; 1ecx, A, C, 69; 1ecx, A, A, 70; 1ecx, A, T, 71; 1ecx, A, M, 146; 1ecx, A, N, 150; 1ecx, A, V, 179; 1ecx, A, Q, 180; 1ecx, A, S, 200; 1ecx, A, H, 202; 1ecx, A, R, 350; 1ef8, A, H, 220; 1eg7, A, S, 1044; 1eg7, A, D, 1046; 1eg7, A, R, 1097; 1eg7, A, Y, 1116; 1eg7, A, R, 1175; 1eg7, A, R, 1178; 1eg7, A, K, 1256; 1eg7, A, G, 1303; 1eg7, A, F, 1304; 1eg7, A, R, 1319; 1eg7, A, N, 1382; 1eg7, A, A, 1383; 1eg7, A, W, 1412; 1eg7, A, Y, 1441; 1eg7, A, N, 1442; 1eg7, A, L, 1443; 1eg7, A, G, 1530; 1eg7, A, I, 1532; 1eg7, A, M, 1533; 1eg7, A, G, 1537; 1emd, A, G, 7; 1emd, A, A, 9; 1emd, A, G, 10; 1emd, A, G, 11; 1emd, A, I, 12; 1emd, A, Y, 33; 1emd, A, D, 34; 1emd, A, I, 35; 1emd, A, S, 76; 1emd, A, A, 77; 1emd, A, G, 78; 1emd, A, V, 79; 1emd, A, R, 80; 1emd, A, R, 81; 1emd, A, R, 87; 1emd, A, N, 94; 1emd, A, I, 97; 1emd, A, L, 101; 1emd, A, I, 117; 1emd, A, T, 118; 1emd, A, N, 119; 1emd, A, V, 121; 1emd, A, V, 146; 1emd, A, L, 149; 1emd, A, R, 153; 1emd, A, G, 210; 1emd, A, S, 222; 1emd, A, A, 223; 1emd, A, T, 224; 1emd, A, M, 227; 1ex1, A, D, 95; 1ex1, A, F, 144; 1ex1, A, R, 158; 1ex1, A, K, 206; 1ex1, A, H, 207; 1ex1, A, E, 220; 1ex1, A, N, 221; 1ex1, A, N, 222; 1ex1, A, M, 250; 1ex1, A, Y, 253; 1ex1, A, S, 255; 1ex1, A, M, 316; 1ex1, A, Y, 425; 1ex1, A, T, 438; 1ex1, A, H, 487; 1ex1, A, D, 495; 1ex1, A, N, 496; 1ex1, A, N, 498; 1ex1, A, T, 500; 1ex1, A, P, 502; 1ex1, A, E, 503; 1eyi, A, G, 26; 1eyi, A, T, 27; 1eyi, A, G, 28; 1eyi, A, E, 29; 1eyi, A, M, 30; 1eyi, A, E, 97; 1eyi, A, K, 112; 1eyi, A, Y, 113; 1eyi, A, D, 118; 1eyi, A, L, 120; 1eyi, A, D, 121; 1eyi, A, G, 122; 1eyi, A, S, 123; 1eyi, A, N, 212; 1eyi, A, Y, 215; 1eyi, A, Y, 244; 1eyi, A, G, 246; 1eyi, A, S, 247; 1eyi, A, M, 248; 1eyi, A, Y, 264; 1eyi, A, K, 274; 1eyi, A, L, 275; 1eyi, A, R, 276; 1eyi, A, E, 280; 1f2d, A, N, 50; 1f2d, A, K, 54; 1f2d, A, N, 79; 1f2d, A, Q, 80; 1f2d, A, S, 166; 1f2d, A, C, 200; 1f2d, A, V, 201; 1f2d, A, T, 202; 1f2d, A, G, 203; 1f2d, A, S, 204; 1f2d, A, T, 205; 1f2d, A, L, 323; 1f2d, A, G, 324; 1f2d, A, G, 325; 1f6d, A, R, 10; 1f6d, A, I, 14; 1f6d, A, Q, 271; 1f6d, A, Y, 273; 1f6d, A, F, 276; 1f6d, A, G, 292; 1f6d, A, E, 296; 1f6d, A, P, 298; 1f6d, A, S, 350; 1f6d, A, A, 352; 1f6d, A, H, 353; 1f6d, A, N, 354; 1f75, A, D, 29; 1f75, A, G, 30; 1f75, A, N, 31; 1f75, A, G, 32; 1f7l, A, D, 8; 1f7l, A, T, 10; 1f7l, A, E, 11; 1f7l, A, R, 14; 1f7l, A, R, 53; 1f7l, A, E, 58; 1f7l, A, N, 84; 1f7l, A, G, 85; 1f7l, A, K, 86; 1f7l, A, P, 87; 1f7l, A, I, 103; 1f7l, A, T, 104; 1f7l, A, E, 108; 1f7u, A, L, 58; 1f7u, A, E, 59; 1f7u, A, W, 60; 1f7u, A, N, 62; 1f7u, A, T, 63; 1f7u, A, R, 66; 1f7u, A, L, 70; 1f7u, A, P, 72; 1f7u, A, P, 74; 1f7u, A, R, 75; 1f7u, A, R, 77; 1f7u, A, N, 106; 1f7u, A, F, 109; 1f7u, A, Q, 111; 1f7u, A, E, 148; 1f7u, A, S, 151; 1f7u, A, N, 153; 1f7u, A, Y, 188; 1f7u, A, G, 193; 1f7u, A, Q, 195; 1f7u, A, E, 294; 1f7u, A, K, 319; 1f7u, A, A, 321; 1f7u, A, K, 332; 1f7u, A, I, 337; 1f7u, A, K, 340; 1f7u, A, S, 341; 1f7u, A, L, 346; 1f7u, A, Y, 347; 1f7u, A, R, 350; 1f7u, A, D, 351; 1f7u, A, Y, 369; 1f7u, A, I, 371; 1f7u, A, Q, 374; 1f7u, A, Q, 375; 1f7u, A, L, 377; 1f7u, A, M, 404; 1f7u, A, Q, 406; 1f7u, A, V, 432; 1f7u, A, M, 433; 1f7u, A, N, 436; 1f7u, A, K, 439; 1f7u, A, G, 465; 1f7u, A, K, 466;

1f7u, A, I, 468; 1f7u, A, N, 469; 1f7u, A, N, 470; 1f7u, A, F, 481; 1f7u, A, E, 482; 1f7u, A, G, 483; 1f7u, A, D, 484; 1f7u, A, P, 487; 1f7u, A, Y, 488; 1f7u, A, Y, 491; 1f7u, A, S, 494; 1f7u, A, R, 495; 1f7u, A, S, 498; 1f7u, A, V, 499; 1f7u, A, N, 502; 1f7u, A, K, 543; 1f7u, A, T, 552; 1f7u, A, H, 559; 1f7u, A, S, 562; 1f7u, A, S, 563; 1f7u, A, Y, 565; 1f7u, A, D, 566; 1f7u, A, L, 568; 1f7u, A, W, 569; 1f7u, A, V, 570; 1f7u, A, A, 571; 1f7u, A, Y, 585; 1f7u, A, R, 606; 1f7u, A, M, 607; 1f8m, A, W, 93; 1f8m, A, D, 108; 1f8m, A, D, 153; 1f8m, A, G, 192; 1f8m, A, H, 193; 1f8m, A, N, 313; 1f8m, A, S, 315; 1f8m, A, S, 317; 1f8m, A, T, 347; 1fgh, A, Q, 72; 1fgh, A, A, 74; 1fgh, A, T, 75; 1fgh, A, S, 166; 1fgh, A, S, 357; 1fgh, A, C, 358; 1fgh, A, C, 421; 1fgh, A, C, 424; 1fgh, A, I, 425; 1fgh, A, N, 446; 1fgh, A, R, 447; 1fgh, A, R, 452; 1fgh, A, R, 580; 1fgh, A, S, 643; 1fgh, A, R, 644; 1fiq, A, Q, 767; 1fiq, A, G, 796; 1fiq, A, G, 797; 1fiq, A, F, 798; 1fiq, A, G, 799; 1fiq, A, E, 802; 1fiq, A, R, 839; 1fiq, A, H, 840; 1fiq, A, L, 873; 1fiq, A, I, 877; 1fiq, A, R, 880; 1fiq, A, T, 909; 1fiq, A, A, 910; 1fiq, A, F, 911; 1fiq, A, R, 912; 1fiq, A, F, 914; 1fiq, A, G, 915; 1fiq, A, Q, 918; 1fiq, A, S, 1008; 1fiq, A, F, 1009; 1fiq, A, T, 1010; 1fiq, A, V, 1011; 1fiq, A, M, 1038; 1fiq, A, G, 1039; 1fiq, A, Q, 1040; 1fiq, A, A, 1078; 1fiq, A, A, 1079; 1fiq, A, S, 1080; 1fiq, A, V, 1081; 1fiq, A, S, 1082; 1fiq, A, Q, 1194; 1fiq, A, G, 1260; 1fnb, A, D, 84; 1fnb, A, H, 90; 1fnb, A, K, 91; 1fnb, A, R, 93; 1fnb, A, L, 94; 1fnb, A, Y, 95; 1fnb, A, C, 114; 1fnb, A, V, 115; 1fnb, A, K, 116; 1fnb, A, L, 118; 1fnb, A, Y, 120; 1fnb, A, G, 130; 1fnb, A, V, 131; 1fnb, A, C, 132; 1fnb, A, S, 133; 1fnb, A, T, 172; 1fnb, A, P, 205; 1fnb, A, S, 234; 1fnb, A, R, 235; 1fnb, A, K, 244; 1fnb, A, Y, 246; 1fnb, A, Y, 314; 1fq0, A, V, 20; 1fq0, A, T, 47; 1fq0, A, R, 49; 1fq0, A, G, 72; 1fq0, A, T, 73; 1fq0, A, P, 94; 1fq0, A, F, 135; 1fq0, A, P, 152; 1fq0, A, T, 161; 1fro, A, Q, 33; 1fro, A, R, 37; 1fro, A, F, 62; 1fro, A, F, 67; 1fro, A, L, 69; 1fro, A, E, 99; 1fro, A, T, 101; 1fro, A, N, 103; 1fro, A, R, 122; 1fro, A, H, 126; 1fro, A, K, 150; 1fro, A, M, 157; 1fro, A, L, 160; 1fro, A, F, 162; 1fro, A, W, 170; 1fro, A, M, 183; 1fua, A, C, 14; 1fua, A, M, 17; 1fua, A, N, 23; 1fua, A, G, 28; 1fua, A, N, 29; 1fua, A, T, 41; 1fua, A, T, 43; 1fua, A, I, 45; 1fua, A, S, 71; 1fua, A, S, 72; 1fua, A, W, 74; 1fua, A, R, 75; 1fua, A, H, 92; 1fua, A, H, 94; 1fua, A, H, 155; 1fuq, A, T, 100; 1fuq, A, R, 126; 1fuq, A, H, 129; 1fuq, A, P, 130; 1fuq, A, N, 131; 1fuq, A, D, 132; 1fuq, A, S, 139; 1fuq, A, S, 140; 1fuq, A, N, 141; 1fwk, A, N, 54; 1fwk, A, I, 55; 1fwk, A, P, 56; 1fwk, A, K, 61; 1fwk, A, N, 62; 1fwk, A, V, 63; 1fwk, A, K, 87; 1fwk, A, A, 91; 1fwk, A, G, 92; 1fwk, A, G, 96; 1fwk, A, S, 97; 1fwk, A, S, 98; 1fwk, A, A, 99; 1fwk, A, S, 101; 1fwk, A, S, 133; 1g64, A, N, 37; 1g64, A, G, 38; 1g64, A, K, 39; 1g64, A, G, 40; 1g64, A, T, 42; 1g64, A, R, 51; 1g64, A, K, 66; 1g64, A, W, 93; 1g64, A, E, 128; 1g64, A, Y, 131; 1g64, A, R, 161; 1g64, A, H, 182; 1g64, A, D, 195; 1g6t, A, P, 8; 1g6t, A, I, 9; 1g6t, A, A, 10; 1g6t, A, R, 11; 1g6t, A, D, 13; 1g6t, A, G, 14; 1g6t, A, S, 23; 1g6t, A, R, 27; 1g6t, A, N, 55; 1g6t, A, T, 58; 1g6t, A, V, 62; 1g6t, A, S, 63; 1g6t, A, Y, 64; 1g6t, A, T, 65; 1g6t, A, L, 66; 1g6t, A, S, 67; 1g6t, A, R, 72; 1g6t, A, E, 89; 1g6t, A, L, 90; 1g6t, A, F, 91; 1g6t, A, N, 94; 1g6t, A, A, 95; 1g6t, A, G, 96; 1g6t, A, T, 97; 1g6t, A, R, 124; 1g6t, A, T, 141; 1g6t, A, L, 143; 1g6t, A, R, 152; 1g6t, A, S, 169; 1g6t, A, S, 170; 1g6t, A, Q, 171; 1g6t, A, S, 197; 1g6t, A, Y, 200; 1g6t, A, T, 259; 1g6t, A, K, 261; 1g6t, A, T, 263; 1g6t, A, D, 292; 1g6t, A, Y, 293; 1g6t, A, R, 298; 1g6t, A, E, 300; 1g6t, A, L, 301; 1g6t, A, F, 324; 1g6t, A, Y, 335; 1g6t, A, N, 336; 1g6t, A, K, 340; 1g6t, A, R, 344; 1g6t, A, H, 363; 1g6t, A, K, 373; 1g6t, A, L, 374; 1g6t, A, A, 380; 1g6t, A, T, 381; 1g6t, A, Y, 382; 1g6t, A, R, 386; 1g6t, A, S, 397; 1g6t, A, D, 398; 1g6t, A, Q, 425; 1g6t, A, A, 426; 1g72, A, E, 55; 1g72, A, C, 103; 1g72, A, C, 104; 1g72, A, V, 107; 1g72, A, R, 109; 1g72, A, T, 153; 1g72, A, S, 168; 1g72, A, G, 169; 1g72, A, A, 170; 1g72, A, E, 171; 1g72, A, T, 235; 1g72, A, W, 237; 1g72, A, N, 255; 1g72, A, R, 324; 1g72, A, N, 387; 1g72, A, W, 467; 1g72, A, G, 530; 1g72, A, W, 531; 1g79, A, R, 23; 1g79, A, R, 24; 1g79, A, C, 43; 1g79, A, D, 49; 1g79, A, P, 50; 1g79, A, T, 51; 1g79, A, R, 67; 1g79, A, I, 68; 1g79, A, V, 69; 1g79, A, L, 70; 1g79, A, K, 72; 1g79, A, Y, 82; 1g79, A, T, 83; 1g79, A, N, 84; 1g79, A, S, 87; 1g79, A, R, 88; 1g79, A, K, 89; 1g79, A, Y, 129; 1g79, A, R, 133; 1g79, A, W, 142; 1g79, A, S, 144; 1g79, A, K, 145; 1g79, A, Q, 146; 1g79, A, S, 147; 1g79, A, S, 148; 1g79, A, R, 149; 1g79, A, F, 177; 1g79, A, F, 202; 1g79, A, R,

215; 1g8f, A, P, 2; 1g8f, A, A, 17; 1g8f, A, L, 18; 1g8f, A, K, 19; 1g8f, A, K, 20; 1g8f, A, N, 21; 1g8f, A, E, 22; 1g8f, A, P, 39; 1g8f, A, C, 43; 1g8f, A, E, 46; 1g8f, A, P, 55; 1g8f, A, L, 56; 1g8f, A, T, 57; 1g8f, A, G, 58; 1g8f, A, D, 64; 1g8f, A, D, 71; 1g8f, A, E, 130; 1g8f, A, R, 134; 1g8f, A, G, 135; 1g8f, A, D, 151; 1g8f, A, P, 164; 1g8f, A, H, 166; 1g8f, A, Y, 167; 1g8f, A, D, 168; 1g8f, A, R, 173; 1g8f, A, K, 174; 1g8f, A, Q, 178; 1g8f, A, E, 182; 1g8f, A, R, 186; 1g8f, A, Q, 187; 1g8f, A, D, 189; 1g8f, A, R, 190; 1g8f, A, Q, 195; 1g8f, A, R, 197; 1g8f, A, H, 235; 1g8f, A, H, 236; 1g8f, A, M, 263; 1g8f, A, G, 282; 1g8f, A, H, 292; 1g8f, A, A, 293; 1g8f, A, K, 297; 1g8f, A, G, 301; 1g8f, A, V, 302; 1g8f, A, D, 303; 1g8f, A, D, 309; 1g8f, A, E, 486; 1g8f, A, D, 489; 1g8f, A, E, 490; 1g8f, A, P, 491; 1g8f, A, H, 494; 1g8f, A, K, 498; 1g99, A, H, 123; 1g99, A, H, 180; 1g99, A, G, 210; 1g99, A, N, 211; 1g99, A, N, 282; 1g99, A, D, 283; 1g99, A, F, 284; 1g99, A, R, 285; 1g99, A, A, 330; 1g99, A, G, 331; 1g99, A, I, 332; 1g99, A, N, 335; 1g99, A, S, 336; 1gal, A, G, 26; 1gal, A, G, 27; 1gal, A, G, 28; 1gal, A, L, 29; 1gal, A, T, 30; 1gal, A, I, 49; 1gal, A, E, 50; 1gal, A, S, 51; 1gal, A, Y, 68; 1gal, A, F, 72; 1gal, A, H, 78; 1gal, A, Y, 80; 1gal, A, T, 87; 1gal, A, N, 89; 1gal, A, R, 95; 1gal, A, G, 97; 1gal, A, N, 98; 1gal, A, G, 99; 1gal, A, G, 102; 1gal, A, S, 103; 1gal, A, V, 106; 1gal, A, N, 107; 1gal, A, G, 108; 1gal, A, G, 109; 1gal, A, T, 110; 1gal, A, Y, 159; 1gal, A, N, 161; 1gal, A, S, 163; 1gal, A, C, 164; 1gal, A, N, 168; 1gal, A, Y, 249; 1gal, A, V, 250; 1gal, A, A, 288; 1gal, A, A, 289; 1gal, A, G, 290; 1gal, A, V, 293; 1gal, A, N, 355; 1gal, A, S, 362; 1gal, A, E, 363; 1gal, A, H, 366; 1gal, A, N, 388; 1gal, A, A, 391; 1gal, A, N, 407; 1gal, A, Y, 509; 1gal, A, Y, 515; 1gal, A, E, 527; 1gal, A, M, 528; 1gal, A, D, 548; 1gal, A, G, 549; 1gal, A, V, 560; 1gal, A, M, 561; 1gal, A, F, 564; 1get, A, I, 10; 1get, A, G, 11; 1get, A, G, 13; 1get, A, S, 14; 1get, A, G, 15; 1get, A, E, 34; 1get, A, A, 35; 1get, A, K, 36; 1get, A, E, 37; 1get, A, G, 40; 1get, A, T, 41; 1get, A, V, 45; 1get, A, G, 46; 1get, A, K, 50; 1get, A, G, 113; 1get, A, F, 114; 1get, A, A, 115; 1get, A, A, 138; 1get, A, T, 139; 1get, A, G, 140; 1get, A, V, 173; 1get, A, G, 174; 1get, A, A, 175; 1get, A, G, 176; 1get, A, Y, 177; 1get, A, I, 178; 1get, A, E, 181; 1get, A, R, 198; 1get, A, R, 204; 1get, A, P, 231; 1get, A, I, 261; 1get, A, G, 262; 1get, A, R, 263; 1get, A, I, 270; 1get, A, G, 302; 1get, A, D, 303; 1get, A, E, 309; 1get, A, L, 310; 1get, A, T, 311; 1get, A, P, 312; 1get, A, T, 341; 1get, A, V, 342; 1get, A, P, 440; 1gim, A, W, 11; 1gim, A, G, 12; 1gim, A, E, 14; 1gim, A, G, 15; 1gim, A, K, 16; 1gim, A, G, 17; 1gim, A, K, 18; 1gim, A, N, 38; 1gim, A, A, 39; 1gim, A, G, 40; 1gim, A, T, 42; 1gim, A, G, 127; 1gim, A, T, 128; 1gim, A, T, 129; 1gim, A, A, 223; 1gim, A, L, 228; 1gim, A, V, 238; 1gim, A, T, 239; 1gim, A, V, 273; 1gim, A, G, 274; 1gim, A, G, 298; 1gim, A, A, 299; 1gim, A, T, 300; 1gim, A, T, 301; 1gim, A, R, 303; 1gim, A, R, 305; 1gim, A, K, 331; 1gim, A, D, 333; 1gim, A, V, 334; 1gim, A, S, 414; 1gim, A, T, 415; 1gim, A, G, 416; 1gim, A, P, 417; 1gp5, A, K, 20; 1gp5, A, E, 21; 1gp5, A, I, 23; 1gp5, A, R, 24; 1gp5, A, K, 128; 1gp5, A, N, 131; 1gp5, A, S, 134; 1gp5, A, G, 135; 1gp5, A, Q, 136; 1gp5, A, Y, 142; 1gp5, A, F, 144; 1gp5, A, N, 215; 1gp5, A, Y, 217; 1gp5, A, V, 229; 1gp5, A, E, 230; 1gp5, A, H, 232; 1gp5, A, T, 233; 1gp5, A, D, 234; 1gp5, A, V, 235; 1gp5, A, S, 236; 1gp5, A, H, 288; 1gp5, A, R, 298; 1gp5, A, S, 300; 1gp5, A, F, 304; 1gp5, A, E, 306; 1gp5, A, F, 334; 1gp5, A, I, 338; 1gp5, A, K, 341; 1gp5, A, L, 342; 1gpa, A, Y, 90; 1gpa, A, G, 135; 1gpa, A, R, 138; 1gpa, A, R, 242; 1gpa, A, R, 310; 1gpa, A, W, 491; 1gpa, A, Y, 573; 1gpa, A, Y, 648; 1gpa, A, R, 649; 1gpa, A, V, 650; 1gpa, A, A, 653; 1gpa, A, G, 675; 1gpa, A, G, 677; 1gpa, A, K, 680; 1gq8, A, W, 227; 1gsa, A, K, 18; 1gsa, A, D, 19; 1gsa, A, S, 20; 1gsa, A, S, 21; 1gsa, A, R, 86; 1gsa, A, D, 88; 1gsa, A, P, 89; 1gsa, A, K, 125; 1gsa, A, I, 158; 1gsa, A, G, 164; 1gsa, A, M, 165; 1gsa, A, G, 166; 1gsa, A, G, 167; 1gsa, A, A, 168; 1gsa, A, I, 170; 1gsa, A, Q, 198; 1gsa, A, N, 199; 1gsa, A, Y, 200; 1gsa, A, L, 201; 1gsa, A, I, 204; 1gsa, A, D, 208; 1gsa, A, T, 232; 1gsa, A, R, 233; 1gsa, A, G, 234; 1gsa, A, N, 235; 1gsa, A, L, 236; 1gsa, A, A, 237; 1gsa, A, D, 273; 1gsa, A, I, 275; 1gsa, A, E, 281; 1gsa, A, N, 283; 1gsa, A, T, 285; 1gsa, A, S, 286; 1gsa, A, P, 287; 1gsa, A, T, 288; 1gtp, A, H, 113; 1gtp, A, S, 135; 1gtp, A, K, 136; 1gtp, A, R, 139; 1gtp, A, R, 185; 1gtp, A, G, 186; 1gxs, A, G, 36; 1gxs, A, R, 37; 1gxs, A, N, 61; 1gxs, A, G, 62; 1gxs, A, G, 63; 1gxs, A, P, 64; 1gxs, A, G, 112; 1gxs, A, F, 113; 1gxs, A,

Y, 115; 1gxs, A, N, 117; 1gxs, A, D, 126; 1gxs, A, E, 157; 1gxs, A, H, 160; 1gxs, A, F, 161; 1gxs, A, N, 249; 1gxs, A, Y, 251; 1gxs, A, F, 269; 1h4g, A, E, 17; 1h4g, A, W, 19; 1h4g, A, L, 47; 1h4g, A, R, 49; 1h4g, A, Y, 85; 1h4g, A, W, 87; 1h4g, A, Y, 96; 1h4g, A, R, 129; 1h4g, A, P, 133; 1h4g, A, S, 134; 1h4g, A, I, 135; 1h4g, A, F, 141; 1h4g, A, Q, 143; 1h4g, A, R, 152; 1h4g, A, T, 153; 1h4g, A, S, 154; 1h7o, A, F, 89; 1h7o, A, C, 133; 1h7o, A, C, 135; 1h7o, A, C, 143; 1h7o, A, Y, 207; 1h7o, A, Y, 216; 1h7o, A, F, 219; 1h7o, A, Y, 287; 1h7o, A, V, 289; 1h7o, A, S, 290; 1h7o, A, Y, 329; 1hdh, A, D, 13; 1hdh, A, D, 14; 1hdh, A, T, 50; 1hdh, A, M, 72; 1hdh, A, G, 138; 1hdh, A, T, 262; 1hdh, A, R, 263; 1hdh, A, N, 318; 1hdh, A, R, 438; 1hdh, A, A, 451; 1hdh, A, H, 452; 1hdh, A, T, 456; 1hdh, A, R, 466; 1hfs, A, D, 107; 1hfs, A, D, 141; 1hfs, A, H, 151; 1hfs, A, D, 153; 1hfs, A, Y, 155; 1hfs, A, D, 158; 1hfs, A, G, 159; 1hfs, A, G, 161; 1hfs, A, N, 162; 1hfs, A, V, 163; 1hfs, A, L, 164; 1hfs, A, A, 165; 1hfs, A, H, 166; 1hfs, A, A, 167; 1hfs, A, Y, 168; 1hfs, A, G, 173; 1hfs, A, N, 175; 1hfs, A, D, 177; 1hfs, A, H, 179; 1hfs, A, D, 181; 1hfs, A, D, 182; 1hfs, A, E, 184; 1hfs, A, L, 197; 1hfs, A, H, 201; 1hfs, A, H, 205; 1hfs, A, H, 211; 1hfs, A, A, 217; 1hfs, A, L, 218; 1hfs, A, Y, 220; 1hfs, A, P, 221; 1hfs, A, L, 222; 1hfs, A, Y, 223; 1hfs, A, H, 224; 1hfs, A, L, 226; 1hrk, A, M, 76; 1hrk, A, L, 92; 1hrk, A, F, 93; 1hrk, A, L, 98; 1hrk, A, M, 99; 1hrk, A, L, 101; 1hrk, A, L, 107; 1hrk, A, R, 114; 1hrk, A, L, 115; 1hrk, A, I, 119; 1hrk, A, C, 196; 1hrk, A, S, 197; 1hrk, A, L, 265; 1hrk, A, P, 266; 1hrk, A, V, 269; 1hrk, A, R, 272; 1hrk, A, V, 305; 1hrk, A, G, 306; 1hrk, A, M, 308; 1hrk, A, W, 310; 1hrk, A, S, 402; 1hrk, A, C, 403; 1hrk, A, C, 406; 1hrk, A, C, 411; 1hv9, A, L, 11; 1hv9, A, A, 13; 1hv9, A, G, 14; 1hv9, A, Q, 76; 1hv9, A, Q, 79; 1hv9, A, L, 80; 1hv9, A, G, 81; 1hv9, A, T, 82; 1hv9, A, Y, 103; 1hv9, A, D, 105; 1hv9, A, Y, 139; 1hv9, A, G, 140; 1hv9, A, E, 154; 1hv9, A, N, 169; 1hv9, A, T, 170; 1hv9, A, Y, 197; 1hv9, A, I, 198; 1hv9, A, T, 199; 1hv9, A, S, 405; 1hv9, A, D, 406; 1hv9, A, A, 422; 1hv9, A, A, 423; 1hv9, A, I, 438; 1hv9, A, R, 440; 1i6p, A, C, 42; 1i6p, A, H, 98; 1i6p, A, C, 101; 1im5, A, D, 52; 1im5, A, H, 54; 1im5, A, H, 71; 1ima, A, D, 90; 1ima, A, I, 92; 1ima, A, D, 93; 1ima, A, G, 94; 1ima, A, E, 162; 1ima, A, G, 194; 1ima, A, T, 195; 1ima, A, A, 196; 1ima, A, E, 213; 1ima, A, G, 215; 1ima, A, I, 216; 1ima, A, D, 220; 1iph, A, I, 114; 1iph, A, D, 118; 1iph, A, R, 125; 1iph, A, V, 127; 1iph, A, R, 165; 1iph, A, G, 184; 1iph, A, V, 199; 1iph, A, G, 200; 1iph, A, F, 206; 1iph, A, F, 214; 1iph, A, I, 274; 1iph, A, H, 275; 1iph, A, F, 391; 1iph, A, L, 407; 1iph, A, R, 411; 1iph, A, S, 414; 1iph, A, Y, 415; 1iph, A, T, 418; 1iph, A, Q, 419; 1iph, A, R, 422; 1ir3, A, L, 1002; 1ir3, A, G, 1003; 1ir3, A, G, 1005; 1ir3, A, S, 1006; 1ir3, A, V, 1010; 1ir3, A, A, 1028; 1ir3, A, K, 1030; 1ir3, A, M, 1076; 1ir3, A, E, 1077; 1ir3, A, M, 1079; 1ir3, A, D, 1083; 1ir3, A, N, 1137; 1ir3, A, D, 1150; 1ir3, A, K, 1165; 1ir3, A, K, 1168; 1ir3, A, G, 1169; 1ir3, A, L, 1170; 1ir3, A, L, 1171; 1ir3, A, P, 1172; 1ir3, A, V, 1173; 1ir3, A, W, 1175; 1ir3, A, N, 1215; 1itx, A, Y, 49; 1itx, A, N, 59; 1itx, A, N, 61; 1itx, A, D, 64; 1itx, A, F, 77; 1itx, A, W, 82; 1itx, A, N, 83; 1itx, A, G, 87; 1itx, A, P, 89; 1itx, A, N, 110; 1itx, A, P, 112; 1itx, A, N, 113; 1itx, A, N, 168; 1itx, A, R, 169; 1itx, A, N, 195; 1itx, A, D, 202; 1itx, A, D, 242; 1itx, A, K, 244; 1itx, A, A, 251; 1itx, A, G, 253; 1itx, A, A, 254; 1itx, A, S, 255; 1itx, A, M, 277; 1itx, A, Y, 279; 1itx, A, D, 280; 1itx, A, D, 343; 1itx, A, G, 344; 1itx, A, Q, 347; 1itx, A, R, 387; 1itx, A, Y, 388; 1itx, A, W, 389; 1itx, A, D, 391; 1itx, A, K, 394; 1itx, A, N, 403; 1itx, A, K, 404; 1itx, A, R, 405; 1itx, A, E, 413; 1itx, A, W, 433; 1j00, A, M, 17; 1j00, A, S, 18; 1j00, A, A, 21; 1j00, A, R, 160; 1j09, A, R, 5; 1j09, A, A, 7; 1j09, A, S, 9; 1j09, A, H, 15; 1j09, A, G, 17; 1j09, A, E, 41; 1j09, A, Y, 187; 1j09, A, N, 191; 1j09, A, R, 205; 1j09, A, A, 206; 1j09, A, E, 208; 1j09, A, W, 209; 1j09, A, L, 235; 1j09, A, L, 236; 1j09, A, K, 243; 1j09, A, I, 244; 1j09, A, S, 245; 1j09, A, R, 247; 1j53, A, R, 7; 1j53, A, Q, 8; 1j53, A, D, 12; 1j53, A, T, 13; 1j53, A, T, 15; 1j53, A, G, 17; 1j53, A, M, 18; 1j53, A, V, 38; 1j53, A, V, 39; 1j53, A, V, 65; 1j53, A, H, 66; 1j53, A, H, 98; 1j53, A, N, 99; 1j53, A, F, 102; 1j53, A, N, 122; 1j53, A, T, 123; 1j53, A, F, 124; 1j53, A, C, 125; 1j53, A, S, 144; 1j53, A, L, 145; 1j53, A, R, 159; 1j53, A, D, 167; 1j79, A, H, 16; 1j79, A, H, 18; 1j79, A, R, 20; 1j79, A, N, 44; 1j79, A, H, 139; 1j79, A, H, 177; 1j79, A, N, 208; 1j79, A, H, 209; 1j79, A, V, 212; 1j79, A, C, 221; 1j79, A, L, 222; 1j79, A, A, 252; 1j79, A, H, 254; 1j79, A,

A, 266; 1j79, A, G, 267; 1jhf, A, T, 27; 1jhf, A, R, 28; 1jhf, A, S, 63; 1jhf, A, R, 64; 1js4, A, W, 209; 1js4, A, S, 210; 1js4, A, G, 211; 1js4, A, D, 214; 1js4, A, E, 215; 1js4, A, W, 256; 1js4, A, W, 260; 1js4, A, D, 261; 1js4, A, D, 262; 1js4, A, R, 317; 1js4, A, Y, 318; 1js4, A, T, 504; 1js4, A, D, 506; 1js4, A, D, 571; 1js4, A, N, 574; 1js4, A, D, 575; 1kae, A, S, 11; 1kae, A, C, 12; 1kae, A, Q, 16; 1kae, A, F, 58; 1kae, A, S, 81; 1kae, A, D, 82; 1kae, A, Y, 130; 1kae, A, P, 132; 1kae, A, G, 133; 1kae, A, S, 140; 1kae, A, P, 162; 1kae, A, G, 185; 1kae, A, G, 186; 1kae, A, Q, 188; 1kae, A, P, 209; 1kae, A, G, 210; 1kae, A, N, 211; 1kae, A, F, 213; 1kae, A, V, 214; 1kae, A, S, 237; 1kae, A, P, 249; 1kae, A, Q, 259; 1kae, A, H, 262; 1kae, A, R, 280; 1kae, A, R, 287; 1kae, A, P, 293; 1kae, A, E, 356; 1kae, A, D, 360; 1kae, A, Y, 361; 1kae, A, H, 367; 1kae, A, E, 414; 1kae, A, L, 416; 1kae, A, H, 419; 1kaz, A, D, 10; 1kaz, A, G, 12; 1kaz, A, T, 13; 1kaz, A, T, 14; 1kaz, A, Y, 15; 1kaz, A, N, 31; 1kaz, A, D, 32; 1kaz, A, Q, 33; 1kaz, A, K, 126; 1kaz, A, Y, 149; 1kaz, A, D, 199; 1kaz, A, G, 201; 1kaz, A, G, 202; 1kaz, A, G, 203; 1kaz, A, T, 204; 1kaz, A, D, 206; 1kaz, A, G, 230; 1kaz, A, E, 268; 1kaz, A, K, 271; 1kaz, A, R, 272; 1kaz, A, S, 275; 1kaz, A, R, 311; 1kaz, A, G, 338; 1kaz, A, G, 339; 1kaz, A, S, 340; 1kaz, A, R, 342; 1kaz, A, I, 343; 1kaz, A, K, 345; 1kaz, A, K, 348; 1kaz, A, D, 366; 1kc7, A, K, 22; 1kc7, A, M, 99; 1kc7, A, P, 100; 1kc7, A, G, 101; 1kc7, A, M, 102; 1kc7, A, M, 103; 1kc7, A, R, 337; 1kc7, A, R, 523; 1kc7, A, W, 527; 1kc7, A, L, 559; 1kc7, A, R, 561; 1kc7, A, R, 617; 1kc7, A, P, 660; 1kc7, A, M, 661; 1kc7, A, R, 665; 1kc7, A, K, 718; 1kc7, A, M, 743; 1kc7, A, E, 745; 1kc7, A, E, 758; 1kc7, A, E, 759; 1kc7, A, G, 766; 1kc7, A, T, 767; 1kc7, A, N, 768; 1kc7, A, D, 769; 1kc7, A, R, 823; 1kc7, A, G, 832; 1ksj, A, L, 24; 1ksj, A, D, 25; 1ksj, A, N, 26; 1ksj, A, A, 27; 1ksj, A, G, 28; 1ksj, A, K, 29; 1ksj, A, T, 30; 1ksj, A, T, 31; 1ksj, A, I, 44; 1ksj, A, P, 46; 1ksj, A, T, 47; 1ksj, A, G, 68; 1ksj, A, G, 69; 1ksj, A, V, 91; 1ksj, A, D, 92; 1ksj, A, D, 95; 1ksj, A, R, 98; 1ksj, A, C, 102; 1ksj, A, N, 125; 1ksj, A, K, 126; 1ksj, A, D, 128; 1ksj, A, L, 129; 1ksj, A, S, 158; 1ksj, A, A, 159; 1ksj, A, V, 160; 1l1r, A, A, 24; 1l1r, A, F, 25; 1l1r, A, K, 26; 1l1r, A, E, 61; 1l1r, A, S, 62; 1l1r, A, M, 99; 1l1r, A, Y, 101; 1l1r, A, D, 124; 1l1r, A, D, 125; 1l1r, A, V, 126; 1l1r, A, A, 128; 1l1r, A, T, 129; 1l1r, A, G, 130; 1l1r, A, G, 131; 1l1r, A, T, 132; 1l7n, A, N, 48; 1l7n, A, S, 99; 1l7n, A, K, 140; 1l7n, A, E, 141; 1l7n, A, D, 167; 1l7n, A, N, 170; 1lam, A, P, 44; 1lam, A, L, 46; 1lam, A, A, 48; 1lam, A, T, 51; 1lam, A, R, 52; 1lam, A, W, 82; 1lam, A, N, 88; 1lam, A, L, 170; 1lam, A, M, 171; 1lam, A, T, 173; 1lam, A, H, 233; 1lam, A, Y, 234; 1lam, A, P, 244; 1lam, A, K, 250; 1lam, A, R, 271; 1lam, A, D, 273; 1lam, A, M, 274; 1lam, A, N, 295; 1lam, A, I, 296; 1lam, A, V, 297; 1lam, A, D, 332; 1lam, A, A, 333; 1lam, A, E, 334; 1lam, A, G, 335; 1lam, A, F, 349; 1lam, A, L, 360; 1lam, A, P, 461; 1ldm, A, V, 28; 1ldm, A, G, 29; 1ldm, A, A, 30; 1ldm, A, V, 31; 1ldm, A, D, 52; 1ldm, A, V, 53; 1ldm, A, M, 54; 1ldm, A, T, 95; 1ldm, A, A, 96; 1ldm, A, G, 97; 1ldm, A, A, 98; 1ldm, A, R, 99; 1ldm, A, Q, 100; 1ldm, A, R, 106; 1ldm, A, L, 109; 1ldm, A, N, 113; 1ldm, A, I, 116; 1ldm, A, I, 120; 1ldm, A, V, 136; 1ldm, A, S, 137; 1ldm, A, N, 138; 1ldm, A, V, 140; 1ldm, A, S, 161; 1ldm, A, L, 165; 1ldm, A, R, 171; 1ldm, A, H, 186; 1ldm, A, W, 188; 1ldm, A, A, 235; 1ldm, A, T, 245; 1ldm, A, I, 249; 1ldm, A, V, 267; 1lj1, A, L, 4; 1lj1, A, F, 7; 1lj1, A, H, 8; 1lj1, A, V, 9; 1lj1, A, Q, 12; 1lj1, A, C, 14; 1lj1, A, S, 16; 1lj1, A, C, 17; 1lj1, A, H, 18; 1lj1, A, L, 24; 1lj1, A, Q, 35; 1lj1, A, C, 36; 1lj1, A, C, 39; 1lj1, A, H, 40; 1lj1, A, G, 41; 1lj1, A, T, 42; 1lj1, A, L, 43; 1lj1, A, V, 46; 1lj1, A, T, 50; 1lj1, A, H, 52; 1lj1, A, H, 54; 1lj1, A, Y, 55; 1lj1, A, N, 56; 1lj1, A, A, 57; 1lj1, A, H, 58; 1lj1, A, S, 60; 1lj1, A, H, 61; 1lj1, A, F, 62; 1lj1, A, V, 66; 1lj1, A, A, 67; 1lj1, A, C, 68; 1lj1, A, T, 69; 1lj1, A, C, 71; 1lj1, A, H, 72; 1lj1, A, S, 73; 1lj1, A, A, 74; 1lj1, A, H, 75; 1lj1, A, V, 80; 1lj1, A, C, 82; 1lj1, A, S, 84; 1lj1, A, C, 85; 1lj1, A, H, 86; 1lj1, A, F, 88; 1lj1, A, F, 90; 1lj1, A, N, 91; 1lj1, A, M, 92; 1lj1, A, Y, 94; 1lj1, A, V, 132; 1lj1, A, G, 133; 1lj1, A, G, 135; 1lj1, A, G, 136; 1lj1, A, A, 137; 1lj1, A, E, 156; 1lj1, A, K, 157; 1lj1, A, E, 158; 1lj1, A, G, 162; 1lj1, A, G, 163; 1lj1, A, N, 164; 1lj1, A, A, 165; 1lj1, A, L, 167; 1lj1, A, A, 168; 1lj1, A, A, 169; 1lj1, A, G, 170; 1lj1, A, G, 171; 1lj1, A, M, 236; 1lj1, A, T, 276; 1lj1, A, R, 277; 1lj1, A, G, 278; 1lj1, A, Y, 298; 1lj1, A, A, 312; 1lj1, A, T, 313; 1lj1, A, G, 314; 1lj1, A, T, 336; 1lj1, A, N, 337; 1lj1, A, Q, 338; 1lj1, A, D, 344; 1lj1, A, H, 365; 1lj1,

A, V, 374; 1lj1, A, M, 375; 1lj1, A, T, 377; 1lj1, A, E, 378; 1lj1, A, K, 431; 1lj1, A, K, 434; 1lj1, A, Y, 435; 1lj1, A, L, 438; 1lj1, A, H, 504; 1lj1, A, H, 505; 1lj1, A, T, 506; 1lj1, A, M, 507; 1lj1, A, G, 508; 1lj1, A, G, 533; 1lj1, A, E, 534; 1lj1, A, T, 536; 1lj1, A, R, 544; 1lj1, A, G, 546; 1lj1, A, G, 547; 1lj1, A, N, 548; 1lj1, A, A, 549; 1lj1, A, I, 550; 1lj1, A, I, 553; 1ljl, A, S, 36; 1ljl, A, T, 63; 1ljl, A, D, 65; 1m21, A, A, 171; 1m21, A, N, 172; 1m21, A, F, 173; 1m21, A, S, 180; 1m21, A, C, 200; 1m21, A, G, 201; 1m21, A, T, 223; 1m21, A, D, 224; 1m21, A, C, 229; 1m21, A, I, 254; 1m21, A, R, 360; 1m21, A, L, 363; 1m21, A, L, 407; 1m21, A, D, 466; 1m21, A, F, 468; 1m21, A, Y, 473; 1mbb, A, L, 44; 1mbb, A, I, 45; 1mbb, A, L, 46; 1mbb, A, G, 47; 1mbb, A, E, 48; 1mbb, A, G, 49; 1mbb, A, S, 50; 1mbb, A, N, 51; 1mbb, A, V, 52; 1mbb, A, N, 65; 1mbb, A, I, 110; 1mbb, A, P, 111; 1mbb, A, G, 112; 1mbb, A, C, 113; 1mbb, A, S, 116; 1mbb, A, I, 119; 1mbb, A, Q, 120; 1mbb, A, I, 122; 1mbb, A, G, 123; 1mbb, A, A, 124; 1mbb, A, Y, 125; 1mbb, A, Y, 158; 1mbb, A, F, 171; 1mbb, A, A, 172; 1mbb, A, I, 173; 1mbb, A, Y, 190; 1mbb, A, R, 214; 1mbb, A, K, 217; 1mbb, A, L, 218; 1mbb, A, N, 226; 1mbb, A, G, 228; 1mbb, A, F, 231; 1mbb, A, K, 232; 1mbb, A, N, 233; 1mbb, A, P, 252; 1mbb, A, Y, 254; 1mbb, A, A, 264; 1mbb, A, G, 266; 1mbb, A, W, 267; 1mbb, A, D, 270; 1mbb, A, K, 275; 1mbb, A, Q, 288; 1mbb, A, A, 289; 1mbb, A, R, 327; 1mht, A, F, 18; 1mht, A, A, 19; 1mht, A, G, 20; 1mht, A, L, 21; 1mht, A, G, 23; 1mht, A, F, 24; 1mht, A, N, 39; 1mht, A, E, 40; 1mht, A, W, 41; 1mht, A, D, 42; 1mht, A, Y, 44; 1mht, A, D, 60; 1mht, A, I, 61; 1mht, A, G, 78; 1mht, A, F, 79; 1mht, A, P, 80; 1mht, A, Q, 82; 1mht, A, S, 85; 1mht, A, I, 86; 1mht, A, S, 87; 1mht, A, G, 88; 1mht, A, K, 89; 1mht, A, Q, 90; 1mht, A, R, 97; 1mht, A, N, 120; 1mht, A, V, 121; 1mht, A, N, 123; 1mht, A, S, 126; 1mht, A, K, 162; 1mht, A, R, 163; 1mht, A, R, 165; 1mht, A, R, 209; 1mht, A, T, 226; 1mht, A, R, 228; 1mht, A, K, 234; 1mht, A, G, 236; 1mht, A, Q, 237; 1mht, A, E, 239; 1mht, A, R, 240; 1mht, A, Y, 242; 1mht, A, I, 249; 1mht, A, T, 250; 1mht, A, S, 252; 1mht, A, A, 253; 1mht, A, Y, 254; 1mht, A, G, 255; 1mht, A, G, 256; 1mht, A, G, 257; 1mht, A, I, 258; 1mht, A, Y, 285; 1mht, A, S, 294; 1mht, A, S, 296; 1mht, A, Q, 297; 1mht, A, G, 303; 1mht, A, N, 304; 1mht, A, S, 305; 1mht, A, V, 306; 1mj9, A, W, 180; 1mj9, A, F, 258; 1mj9, A, L, 259; 1mj9, A, A, 303; 1mj9, A, I, 305; 1mj9, A, L, 306; 1mj9, A, T, 307; 1mj9, A, Q, 312; 1mj9, A, R, 313; 1mj9, A, M, 314; 1mj9, A, G, 315; 1mj9, A, G, 317; 1mj9, A, K, 318; 1mj9, A, L, 341; 1mj9, A, S, 342; 1mj9, A, L, 344; 1mj9, A, G, 345; 1mj9, A, S, 348; 1mj9, A, R, 421; 1mka, A, Q, 27; 1mka, A, F, 71; 1mka, A, M, 77; 1mka, A, P, 78; 1mka, A, W, 87; 1mka, A, G, 91; 1mka, A, R, 104; 1mka, A, A, 105; 1mka, A, L, 106; 1mka, A, F, 113; 1mka, A, G, 115; 1mka, A, Q, 116; 1mka, A, F, 171; 1moq, A, Y, 248; 1moq, A, Y, 257; 1moq, A, E, 258; 1moq, A, A, 262; 1moq, A, N, 265; 1moq, A, C, 300; 1moq, A, G, 301; 1moq, A, T, 302; 1moq, A, S, 303; 1moq, A, S, 347; 1moq, A, Q, 348; 1moq, A, S, 349; 1moq, A, T, 352; 1moq, A, N, 392; 1moq, A, A, 393; 1moq, A, V, 399; 1moq, A, S, 401; 1moq, A, H, 465; 1moq, A, H, 466; 1moq, A, D, 474; 1moq, A, A, 520; 1moq, A, N, 522; 1moq, A, N, 523; 1moq, A, L, 526; 1moq, A, E, 527; 1moq, A, M, 559; 1moq, A, E, 569; 1moq, A, A, 572; 1moq, A, A, 602; 1moq, A, S, 604; 1mrq, A, H, 14; 1mrq, A, F, 15; 1mrq, A, G, 22; 1mrq, A, T, 23; 1mrq, A, Y, 24; 1mrq, A, L, 54; 1mrq, A, F, 80; 1mrq, A, E, 127; 1mrq, A, V, 128; 1mrq, A, I, 129; 1mrq, A, S, 166; 1mrq, A, N, 167; 1mrq, A, Q, 190; 1mrq, A, Y, 216; 1mrq, A, S, 217; 1mrq, A, A, 218; 1mrq, A, L, 219; 1mrq, A, G, 220; 1mrq, A, S, 221; 1mrq, A, H, 222; 1mrq, A, W, 227; 1mrq, A, L, 236; 1mrq, A, A, 253; 1mrq, A, L, 268; 1mrq, A, A, 269; 1mrq, A, K, 270; 1mrq, A, S, 271; 1mrq, A, Y, 272; 1mrq, A, N, 273; 1mrq, A, R, 276; 1mrq, A, Q, 279; 1mrq, A, N, 280; 1mrq, A, L, 306; 1mrq, A, L, 308; 1mud, A, W, 24; 1mud, A, L, 40; 1mud, A, R, 58; 1mud, A, D, 64; 1mud, A, N, 67; 1mud, A, A, 68; 1mud, A, R, 147; 1mud, A, Q, 182; 1mud, A, M, 185; 1mud, A, C, 192; 1mud, A, T, 193; 1mud, A, R, 194; 1mud, A, S, 195; 1mud, A, P, 197; 1mud, A, C, 199; 1mud, A, C, 202; 1mud, A, Q, 205; 1mud, A, C, 208; 1mud, A, A, 211; 1mvn, A, S, 27; 1mvn, A, G, 28; 1mvn, A, S, 29; 1mvn, A, V, 30; 1mvn, A, A, 31; 1mvn, A, K, 34; 1mvn, A, T, 53; 1mvn, A, S, 55; 1mvn, A, F, 59; 1mvn, A, S, 106; 1mvn, A, A, 107; 1mvn, A, N, 108; 1mvn, A, T, 109; 1mvn, A, A, 140;

1mvn, A, M, 141; 1mvn, A, N, 142; 1mvn, A, M, 145; 1mvn, A, R, 172; 1mvn, A, L, 173; 1mvn, A, A, 174; 1mvn, A, G, 181; 1mvn, A, A, 182; 1mvn, A, M, 183; 1myr, A, Q, 7; 1myr, A, E, 8; 1myr, A, N, 9; 1myr, A, T, 17; 1myr, A, D, 18; 1myr, A, G, 19; 1myr, A, N, 21; 1myr, A, S, 24; 1myr, A, Q, 39; 1myr, A, F, 54; 1myr, A, H, 56; 1myr, A, R, 57; 1myr, A, D, 60; 1myr, A, H, 66; 1myr, A, D, 70; 1myr, A, N, 90; 1myr, A, K, 108; 1myr, A, R, 109; 1myr, A, R, 111; 1myr, A, V, 113; 1myr, A, I, 129; 1myr, A, G, 132; 1myr, A, T, 134; 1myr, A, H, 141; 1myr, A, Q, 146; 1myr, A, Q, 149; 1myr, A, D, 150; 1myr, A, E, 153; 1myr, A, K, 165; 1myr, A, L, 170; 1myr, A, E, 173; 1myr, A, N, 186; 1myr, A, R, 194; 1myr, A, P, 203; 1myr, A, R, 205; 1myr, A, S, 207; 1myr, A, Y, 215; 1myr, A, N, 218; 1myr, A, T, 221; 1myr, A, D, 239; 1myr, A, L, 240; 1myr, A, N, 244; 1myr, A, H, 247; 1myr, A, Q, 248; 1myr, A, G, 249; 1myr, A, R, 259; 1myr, A, Y, 264; 1myr, A, N, 265; 1myr, A, D, 266; 1myr, A, T, 267; 1myr, A, D, 268; 1myr, A, R, 269; 1myr, A, H, 270; 1myr, A, A, 273; 1myr, A, R, 277; 1myr, A, N, 292; 1myr, A, T, 294; 1myr, A, Q, 297; 1myr, A, I, 300; 1myr, A, R, 306; 1myr, A, Q, 333; 1myr, A, T, 345; 1myr, A, N, 346; 1myr, A, L, 357; 1myr, A, N, 361; 1myr, A, A, 362; 1myr, A, S, 363; 1myr, A, H, 365; 1myr, A, Y, 427; 1myr, A, W, 457; 1myr, A, E, 464; 1myr, A, F, 465; 1myr, A, N, 482; 1myr, A, D, 485; 1myr, A, K, 490; 1myr, A, S, 500; 1myr, A, P, 501; 1n2c, A, Y, 64; 1n2c, A, V, 70; 1n2c, A, P, 85; 1n2c, A, V, 86; 1n2c, A, C, 88; 1n2c, A, Y, 91; 1n2c, A, E, 153; 1n2c, A, I, 159; 1n2c, A, G, 185; 1n2c, A, Q, 191; 1n2c, A, Y, 229; 1n2c, A, I, 231; 1n2c, A, C, 275; 1n2c, A, S, 278; 1n2c, A, I, 355; 1n2c, A, G, 356; 1n2c, A, G, 357; 1n2c, A, L, 358; 1n2c, A, R, 359; 1n2c, A, F, 381; 1n2c, A, G, 424; 1n2c, A, I, 425; 1n2c, A, K, 426; 1n2c, A, K, 433; 1n2c, A, E, 440; 1n2c, A, H, 442; 1ndh, A, R, 63; 1ndh, A, P, 64; 1ndh, A, T, 66; 1ndh, A, I, 81; 1ndh, A, K, 82; 1ndh, A, V, 83; 1ndh, A, F, 85; 1ndh, A, K, 86; 1ndh, A, K, 91; 1ndh, A, G, 95; 1ndh, A, G, 96; 1ndh, A, K, 97; 1ndh, A, M, 98; 1ndh, A, S, 99; 1ndh, A, T, 153; 1ndh, A, T, 156; 1ni4, A, Y, 89; 1ni4, A, R, 90; 1ni4, A, G, 136; 1ni4, A, I, 137; 1ni4, A, V, 138; 1ni4, A, G, 166; 1ni4, A, D, 167; 1ni4, A, G, 168; 1ni4, A, A, 169; 1ni4, A, N, 196; 1ni4, A, Y, 198; 1ni4, A, G, 199; 1nvt, A, H, 19; 1nvt, A, G, 135; 1nvt, A, A, 136; 1nvt, A, G, 137; 1nvt, A, G, 138; 1nvt, A, A, 139; 1nvt, A, N, 157; 1nvt, A, R, 158; 1nvt, A, T, 159; 1nvt, A, K, 162; 1nvt, A, L, 187; 1nvt, A, A, 200; 1nvt, A, T, 201; 1nvt, A, P, 202; 1nvt, A, I, 203; 1nvt, A, M, 205; 1nvt, A, L, 229; 1nvt, A, I, 230; 1nvt, A, Y, 231; 1nvt, A, G, 252; 1nvt, A, M, 255; 1nvt, A, L, 256; 1nzy, A, R, 22; 1nzy, A, H, 23; 1nzy, A, R, 24; 1nzy, A, A, 26; 1nzy, A, G, 49; 1nzy, A, A, 62; 1nzy, A, G, 63; 1nzy, A, Y, 65; 1nzy, A, L, 66; 1nzy, A, R, 67; 1nzy, A, W, 89; 1nzy, A, G, 113; 1nzy, A, A, 136; 1nzy, A, I, 140; 1nzy, A, T, 146; 1nzy, A, L, 202; 1nzy, A, A, 203; 1nzy, A, A, 205; 1nzy, A, T, 207; 1nzy, A, Q, 210; 1nzy, A, F, 252; 1nzy, A, R, 257; 1nzy, A, A, 258; 1nzy, A, D, 259; 1o04, A, Q, 14; 1o04, A, F, 18; 1o04, A, T, 39; 1o04, A, V, 40; 1o04, A, N, 41; 1o04, A, T, 44; 1o04, A, E, 46; 1o04, A, V, 47; 1o04, A, I, 48; 1o04, A, F, 70; 1o04, A, L, 72; 1o04, A, Y, 101; 1o04, A, L, 108; 1o04, A, D, 109; 1o04, A, I, 146; 1o04, A, D, 147; 1o04, A, F, 150; 1o04, A, F, 151; 1o04, A, Y, 153; 1o04, A, R, 155; 1o04, A, E, 157; 1o04, A, P, 158; 1o04, A, V, 159; 1o04, A, I, 165; 1o04, A, I, 166; 1o04, A, P, 167; 1o04, A, W, 168; 1o04, A, N, 169; 1o04, A, M, 174; 1o04, A, W, 177; 1o04, A, A, 194; 1o04, A, E, 195; 1o04, A, Q, 196; 1o04, A, Y, 203; 1o04, A, G, 225; 1o04, A, P, 226; 1o04, A, G, 229; 1o04, A, A, 230; 1o04, A, F, 243; 1o04, A, T, 244; 1o04, A, G, 245; 1o04, A, S, 246; 1o04, A, I, 249; 1o04, A, I, 253; 1o04, A, L, 269; 1o04, A, G, 270; 1o04, A, F, 401; 1o04, A, N, 440; 1o04, A, Y, 441; 1o04, A, S, 443; 1o04, A, Q, 444; 1o04, A, A, 445; 1o04, A, V, 458; 1o04, A, F, 459; 1o04, A, F, 465; 1o04, A, Y, 468; 1o04, A, Q, 497; 1o98, A, D, 12; 1o98, A, R, 17; 1o98, A, E, 19; 1o98, A, N, 61; 1o98, A, H, 123; 1o98, A, R, 134; 1o98, A, R, 153; 1o98, A, K, 170; 1o98, A, Y, 174; 1o98, A, R, 185; 1o98, A, R, 191; 1o98, A, R, 194; 1o98, A, D, 196; 1o98, A, R, 197; 1o98, A, R, 203; 1o98, A, Y, 207; 1o98, A, R, 264; 1o98, A, R, 282; 1o98, A, G, 283; 1o98, A, P, 284; 1o98, A, H, 297; 1o98, A, S, 299; 1o98, A, E, 300; 1o98, A, K, 336; 1o98, A, Y, 337; 1o98, A, F, 342; 1o98, A, E, 349; 1o98, A, E, 351; 1o98, A, R, 357; 1o98, A, D, 403; 1o98, A, H, 407; 1o98, A, D, 444; 1o98, A, H, 445; 1o98, A, N, 447; 1o98, A, H, 462; 1oas,

A, N, 71; 1oas, A, G, 174; 1oas, A, V, 175; 1oas, A, G, 176; 1oas, A, T, 177; 1oas, A, G, 178; 1oas, A, G, 179; 1oas, A, T, 180; 1oas, A, Q, 227; 1oas, A, G, 228; 1oas, A, I, 229; 1oas, A, P, 299; 1oas, A, S, 300; 1oas, A, Y, 305; 1oh9, A, G, 10; 1oh9, A, G, 43; 1oh9, A, G, 44; 1oh9, A, L, 65; 1oh9, A, R, 66; 1oh9, A, L, 80; 1oh9, A, V, 122; 1oh9, A, S, 147; 1oh9, A, N, 158; 1oh9, A, V, 159; 1oh9, A, N, 160; 1oh9, A, A, 161; 1oh9, A, D, 162; 1oh9, A, S, 180; 1oh9, A, G, 184; 1oh9, A, I, 185; 1oh9, A, L, 186; 1oh9, A, I, 209; 1oh9, A, I, 210; 1oh9, A, T, 211; 1oh9, A, D, 212; 1oh9, A, G, 213; 1oh9, A, M, 214; 1oj4, A, N, 12; 1oj4, A, G, 24; 1oj4, A, Y, 25; 1oj4, A, H, 26; 1oj4, A, L, 28; 1oj4, A, F, 32; 1oj4, A, V, 60; 1oj4, A, N, 65; 1oj4, A, L, 66; 1oj4, A, K, 96; 1oj4, A, G, 101; 1oj4, A, G, 102; 1oj4, A, G, 103; 1oj4, A, L, 104; 1oj4, A, G, 105; 1oj4, A, G, 106; 1oj4, A, G, 107; 1oj4, A, S, 108; 1oj4, A, N, 110; 1oj4, A, G, 139; 1oj4, A, A, 140; 1oj4, A, R, 147; 1oj4, A, V, 156; 1oj4, A, T, 181; 1oj4, A, P, 182; 1oj4, A, F, 185; 1os7, A, H, 70; 1os7, A, Y, 73; 1os7, A, L, 85; 1os7, A, N, 95; 1os7, A, H, 99; 1os7, A, D, 101; 1os7, A, V, 102; 1os7, A, F, 104; 1os7, A, L, 114; 1os7, A, T, 126; 1os7, A, F, 159; 1os7, A, F, 206; 1os7, A, W, 240; 1os7, A, H, 255; 1os7, A, R, 266; 1os7, A, M, 268; 1pmi, A, H, 113; 1pmi, A, E, 138; 1pmi, A, H, 285; 1pud, A, C, 318; 1pud, A, C, 320; 1pud, A, C, 323; 1pud, A, H, 349; 1q3q, A, T, 42; 1q3q, A, L, 43; 1q3q, A, G, 44; 1q3q, A, P, 45; 1q3q, A, N, 63; 1q3q, A, C, 65; 1q3q, A, G, 94; 1q3q, A, D, 95; 1q3q, A, G, 96; 1q3q, A, T, 99; 1q3q, A, T, 160; 1q3q, A, T, 163; 1q3q, A, A, 410; 1q3q, A, G, 411; 1q3q, A, I, 447; 1q3q, A, L, 451; 1q3q, A, I, 479; 1q3q, A, I, 494; 1q3q, A, E, 496; 1qcn, A, D, 126; 1qcn, A, Y, 128; 1qcn, A, V, 137; 1qcn, A, R, 142; 1qcn, A, Y, 159; 1qcn, A, E, 201; 1qcn, A, D, 233; 1qcn, A, P, 246; 1qcn, A, L, 247; 1qf6, A, T, 111; 1qf6, A, K, 200; 1qf6, A, T, 201; 1qf6, A, A, 202; 1qf6, A, G, 203; 1qf6, A, A, 204; 1qf6, A, Y, 205; 1qf6, A, G, 208; 1qf6, A, S, 210; 1qf6, A, M, 214; 1qf6, A, R, 217; 1qf6, A, Y, 219; 1qf6, A, R, 245; 1qf6, A, E, 258; 1qf6, A, P, 261; 1qf6, A, H, 309; 1qf6, A, Y, 313; 1qf6, A, A, 316; 1qf6, A, M, 317; 1qf6, A, P, 331; 1qf6, A, C, 334; 1qf6, A, E, 365; 1qf6, A, P, 366; 1qf6, A, S, 367; 1qf6, A, G, 368; 1qf6, A, S, 369; 1qf6, A, L, 370; 1qf6, A, M, 374; 1qf6, A, R, 375; 1qf6, A, V, 376; 1qf6, A, F, 379; 1qf6, A, Q, 381; 1qf6, A, D, 383; 1qf6, A, H, 385; 1qf6, A, Y, 462; 1qf6, A, R, 476; 1qf6, A, Q, 479; 1qf6, A, C, 480; 1qf6, A, T, 482; 1qf6, A, Q, 484; 1qf6, A, H, 511; 1qf6, A, G, 516; 1qf6, A, S, 517; 1qf6, A, R, 520; 1qf6, A, I, 547; 1qf6, A, T, 548; 1qf6, A, D, 549; 1qf6, A, N, 575; 1qf6, A, E, 576; 1qf6, A, K, 577; 1qf6, A, I, 578; 1qf6, A, G, 579; 1qf6, A, F, 580; 1qf6, A, R, 583; 1qf6, A, R, 589; 1qf6, A, V, 595; 1qf6, A, G, 597; 1qf6, A, D, 598; 1qf6, A, K, 599; 1qf6, A, E, 600; 1qf6, A, A, 607; 1qf6, A, R, 609; 1qf6, A, R, 612; 1qh5, A, H, 54; 1qh5, A, H, 56; 1qh5, A, H, 59; 1qh5, A, H, 110; 1qh5, A, D, 134; 1qh5, A, F, 137; 1qh5, A, C, 141; 1qh5, A, G, 142; 1qh5, A, K, 143; 1qh5, A, Y, 145; 1qh5, A, H, 173; 1qh5, A, Y, 175; 1qh5, A, R, 249; 1qh5, A, K, 252; 1qhf, A, R, 7; 1qhf, A, G, 9; 1qhf, A, Q, 10; 1qhf, A, S, 11; 1qhf, A, N, 14; 1qhf, A, F, 19; 1qhf, A, T, 20; 1qhf, A, Y, 89; 1qhf, A, K, 97; 1qhf, A, R, 113; 1qhf, A, T, 207; 1qhf, A, V, 240; 1qlh, A, C, 46; 1qlh, A, R, 47; 1qlh, A, H, 67; 1qlh, A, C, 97; 1qlh, A, G, 98; 1qlh, A, C, 100; 1qlh, A, C, 103; 1qlh, A, C, 111; 1qlh, A, C, 174; 1qlh, A, T, 178; 1qlh, A, G, 199; 1qlh, A, G, 201; 1qlh, A, V, 203; 1qlh, A, D, 223; 1qlh, A, I, 224; 1qlh, A, N, 225; 1qlh, A, K, 228; 1qlh, A, I, 269; 1qlh, A, R, 271; 1qlh, A, T, 274; 1qlh, A, V, 292; 1qlh, A, A, 293; 1qlh, A, A, 317; 1qlh, A, I, 318; 1qlh, A, F, 319; 1qpr, A, R, 48; 1qpr, A, T, 138; 1qpr, A, R, 139; 1qpr, A, H, 161; 1qpr, A, R, 162; 1qpr, A, L, 170; 1qpr, A, K, 172; 1qpr, A, D, 173; 1qpr, A, S, 248; 1qpr, A, G, 249; 1qpr, A, G, 270; 1qpr, A, A, 271; 1qpr, A, H, 274; 1qpr, A, D, 280; 1qtn, A, R, 260; 1qtn, A, E, 290; 1qtn, A, E, 294; 1qtn, A, G, 318; 1qtn, A, Y, 334; 1qtn, A, T, 337; 1qtn, A, Q, 358; 1qum, A, H, 7; 1qum, A, S, 9; 1qum, A, A, 10; 1qum, A, A, 11; 1qum, A, G, 12; 1qum, A, F, 32; 1qum, A, N, 35; 1qum, A, Q, 36; 1qum, A, R, 37; 1qum, A, Q, 38; 1qum, A, W, 39; 1qum, A, H, 69; 1qum, A, Y, 72; 1qum, A, L, 73; 1qum, A, N, 75; 1qum, A, H, 78; 1qum, A, H, 109; 1qum, A, L, 114; 1qum, A, E, 145; 1qum, A, Q, 150; 1qum, A, G, 151; 1qum, A, S, 152; 1qum, A, D, 179; 1qum, A, H, 182; 1qum, A, H, 216; 1qum, A, V, 228; 1qum, A, D, 229; 1qum, A, R, 230; 1qum, A, H, 231; 1qz9, A, S, 36; 1qz9, A, T, 96; 1qz9, A, T, 97; 1qz9, A, S, 98; 1qz9, A, T, 131; 1qz9, A,

D, 132; 1qz9, A, T, 172; 1qz9, A, A, 203; 1qz9, A, H, 204; 1qz9, A, C, 224; 1qz9, A, Y, 226; 1qz9, A, R, 369; 1qz9, A, R, 375; 1ra2, A, A, 6; 1ra2, A, A, 7; 1ra2, A, I, 14; 1ra2, A, G, 15; 1ra2, A, M, 16; 1ra2, A, K, 32; 1ra2, A, G, 43; 1ra2, A, R, 44; 1ra2, A, H, 45; 1ra2, A, T, 46; 1ra2, A, I, 50; 1ra2, A, R, 52; 1ra2, A, R, 57; 1ra2, A, L, 62; 1ra2, A, S, 63; 1ra2, A, S, 64; 1ra2, A, K, 76; 1ra2, A, V, 78; 1ra2, A, G, 95; 1ra2, A, G, 96; 1ra2, A, G, 97; 1ra2, A, R, 98; 1ra2, A, V, 99; 1ra2, A, Y, 100; 1ra2, A, Q, 102; 1ra2, A, T, 113; 1ra2, A, T, 123; 1rdd, A, D, 10; 1rdd, A, G, 11; 1rdd, A, N, 44; 1rdd, A, E, 48; 1req, A, A, 41; 1req, A, E, 42; 1req, A, Y, 75; 1req, A, T, 77; 1req, A, M, 78; 1req, A, F, 81; 1req, A, R, 82; 1req, A, T, 85; 1req, A, R, 87; 1req, A, S, 114; 1req, A, A, 116; 1req, A, F, 117; 1req, A, L, 119; 1req, A, A, 139; 1req, A, S, 164; 1req, A, T, 166; 1req, A, T, 195; 1req, A, V, 206; 1req, A, R, 207; 1req, A, T, 209; 1req, A, N, 236; 1req, A, Y, 243; 1req, A, E, 247; 1req, A, A, 250; 1req, A, T, 251; 1req, A, R, 283; 1req, A, S, 285; 1req, A, F, 287; 1req, A, M, 292; 1req, A, N, 293; 1req, A, R, 326; 1req, A, T, 327; 1req, A, H, 328; 1req, A, Q, 330; 1req, A, G, 333; 1req, A, W, 334; 1req, A, L, 336; 1req, A, Q, 361; 1req, A, S, 362; 1req, A, E, 370; 1req, A, A, 371; 1req, A, A, 373; 1req, A, L, 374; 1req, A, Q, 454; 1req, A, L, 602; 1req, A, G, 609; 1req, A, D, 611; 1req, A, R, 612; 1req, A, G, 613; 1req, A, I, 617; 1req, A, Y, 621; 1req, A, G, 653; 1req, A, S, 655; 1req, A, L, 657; 1req, A, A, 658; 1req, A, G, 659; 1req, A, G, 685; 1req, A, G, 686; 1req, A, V, 687; 1req, A, Y, 705; 1req, A, T, 706; 1req, A, P, 707; 1req, A, T, 709; 1req, A, S, 714; 1s3i, A, T, 31; 1s3i, A, I, 32; 1s3i, A, F, 56; 1s3i, A, W, 59; 1s3i, A, C, 86; 1s3i, A, Q, 88; 1s3i, A, Y, 105; 1s3i, A, G, 115; 1s3i, A, L, 146; 1s3i, A, L, 147; 1s3i, A, C, 152; 1s3i, A, E, 153; 1s3i, A, R, 166; 1s3i, A, P, 189; 1s3i, A, C, 191; 1s3i, A, C, 238; 1s95, A, K, 192; 1s95, A, Q, 196; 1s95, A, D, 242; 1s95, A, H, 244; 1s95, A, L, 251; 1s95, A, E, 255; 1s95, A, D, 271; 1s95, A, H, 352; 1snn, A, R, 25; 1snn, A, E, 26; 1snn, A, D, 96; 1snn, A, E, 97; 1snn, A, S, 100; 1snn, A, F, 101; 1snn, A, L, 151; 1snn, A, R, 161; 1snn, A, G, 163; 1snn, A, H, 164; 1snn, A, T, 165; 1snn, A, I, 183; 1snn, A, E, 204; 1snn, A, H, 206; 1snn, A, N, 207; 1t4c, A, H, 15; 1t4c, A, V, 16; 1t4c, A, A, 18; 1t4c, A, R, 38; 1t4c, A, M, 44; 1t4c, A, L, 72; 1t4c, A, D, 73; 1t4c, A, M, 74; 1t4c, A, K, 75; 1t4c, A, N, 96; 1t4c, A, F, 97; 1t4c, A, G, 98; 1t4c, A, A, 101; 1t4c, A, R, 104; 1t4c, A, M, 105; 1t4c, A, V, 124; 1t4c, A, K, 125; 1t4c, A, V, 138; 1t4c, A, Y, 139; 1t4c, A, M, 200; 1tdj, A, F, 61; 1tdj, A, N, 89; 1tdj, A, F, 157; 1tdj, A, G, 188; 1tdj, A, G, 189; 1tdj, A, G, 190; 1tdj, A, G, 191; 1tdj, A, L, 192; 1tdj, A, G, 241; 1tdj, A, V, 242; 1tdj, A, E, 286; 1tdj, A, S, 288; 1tdj, A, G, 316; 1tmo, A, G, 117; 1tmo, A, W, 118; 1tmo, A, R, 119; 1tmo, A, A, 120; 1tmo, A, S, 127; 1tmo, A, S, 187; 1tmo, A, K, 192; 1tmo, A, N, 193; 1tmo, A, V, 196; 1tmo, A, I, 226; 1tmo, A, D, 227; 1tmo, A, P, 228; 1tmo, A, T, 231; 1tmo, A, Q, 247; 1tmo, A, D, 249; 1tmo, A, W, 328; 1tmo, A, C, 329; 1tmo, A, R, 332; 1tmo, A, Q, 333; 1tmo, A, G, 441; 1tmo, A, N, 442; 1tmo, A, N, 443; 1tmo, A, N, 446; 1tmo, A, H, 447; 1tmo, A, Q, 449; 1tmo, A, V, 467; 1tmo, A, D, 468; 1tmo, A, T, 472; 1tmo, A, A, 484; 1tmo, A, R, 490; 1tmo, A, D, 520; 1tmo, A, W, 646; 1tmo, A, Q, 648; 1tmo, A, S, 649; 1tmo, A, C, 650; 1tmo, A, H, 651; 1tmo, A, P, 652; 1tmo, A, R, 655; 1tmo, A, L, 656; 1tmo, A, H, 657; 1tmo, A, S, 658; 1tmo, A, Q, 659; 1tmo, A, E, 724; 1tmo, A, N, 753; 1tmo, A, Y, 771; 1trk, A, A, 33; 1trk, A, H, 69; 1trk, A, G, 116; 1trk, A, P, 117; 1trk, A, L, 118; 1trk, A, G, 156; 1trk, A, D, 157; 1trk, A, G, 158; 1trk, A, E, 162; 1trk, A, N, 187; 1trk, A, I, 189; 1trk, A, T, 190; 1trk, A, I, 191; 1trk, A, I, 250; 1trk, A, D, 382; 1trk, A, I, 416; 1trk, A, E, 418; 1trk, A, F, 445; 1trk, A, Y, 448; 1trk, A, H, 481; 1tys, A, R, 21; 1tys, A, H, 51; 1tys, A, I, 79; 1tys, A, W, 80; 1tys, A, W, 83; 1tys, A, L, 143; 1tys, A, H, 147; 1tys, A, Q, 165; 1tys, A, S, 167; 1tys, A, C, 168; 1tys, A, L, 172; 1tys, A, G, 173; 1tys, A, F, 176; 1tys, A, N, 177; 1tys, A, H, 207; 1tys, A, Y, 209; 1tys, A, V, 262; 1tys, A, A, 263; 1uae, A, K, 22; 1uae, A, R, 91; 1uae, A, W, 95; 1uae, A, G, 114; 1uae, A, I, 117; 1uae, A, R, 120; 1uae, A, P, 121; 1uae, A, V, 122; 1uae, A, D, 123; 1uae, A, L, 124; 1uae, A, H, 125; 1uae, A, K, 160; 1uae, A, V, 161; 1uae, A, S, 162; 1uae, A, V, 163; 1uae, A, G, 164; 1uae, A, T, 304; 1uae, A, V, 327; 1uae, A, F, 328; 1uae, A, R, 331; 1uaq, A, I, 33; 1uaq, A, N, 51; 1uaq, A, H, 62; 1uaq, A, G, 63; 1uaq, A, P, 90; 1uaq, A, C, 94; 1uaq, A, D, 155; 1uas, A, T, 8; 1uas, A, P, 9; 1uas, A, Q, 10; 1uas,

A, M, 11; 1uas, A, W, 16; 1uas, A, G, 44; 1uas, A, Q, 46; 1uas, A, Y, 47; 1uas, A, D, 51; 1uas, A, D, 52; 1uas, A, Y, 57; 1uas, A, Y, 93; 1uas, A, S, 98; 1uas, A, Q, 99; 1uas, A, C, 101; 1uas, A, S, 102; 1uas, A, P, 106; 1uas, A, L, 109; 1uas, A, D, 110; 1uas, A, E, 112; 1uas, A, E, 113; 1uas, A, K, 128; 1uas, A, A, 135; 1uas, A, R, 137; 1uas, A, M, 140; 1uas, A, R, 145; 1uas, A, C, 162; 1uas, A, W, 164; 1uas, A, P, 169; 1uas, A, A, 170; 1uas, A, T, 171; 1uas, A, W, 172; 1uas, A, A, 173; 1uas, A, G, 174; 1uas, A, M, 176; 1uas, A, W, 180; 1uas, A, R, 181; 1uas, A, T, 183; 1uas, A, G, 184; 1uas, A, W, 190; 1uas, A, T, 194; 1uas, A, R, 196; 1uas, A, E, 199; 1uas, A, Y, 206; 1uas, A, M, 217; 1uas, A, S, 226; 1uas, A, E, 229; 1uas, A, Q, 271; 1uas, A, R, 306; 1uas, A, P, 351; 1uas, A, H, 352; 1un1, A, L, 91; 1un1, A, G, 92; 1un1, A, N, 93; 1un1, A, Q, 97; 1un1, A, P, 98; 1un1, A, I, 100; 1un1, A, Y, 118; 1un1, A, A, 180; 1un1, A, T, 181; 1un1, A, R, 182; 1uqr, A, G, 53; 1uqr, A, E, 54; 1uqr, A, E, 55; 1uqr, A, N, 74; 1uqr, A, T, 81; 1uqr, A, S, 82; 1uqr, A, L, 101; 1uqr, A, S, 102; 1uqt, A, W, 85; 1uqt, A, Q, 185; 1uqt, A, I, 225; 1uqt, A, V, 260; 1uqt, A, R, 262; 1uqt, A, K, 267; 1uqt, A, P, 297; 1uqt, A, H, 338; 1uqt, A, F, 339; 1uqt, A, R, 341; 1uqt, A, L, 344; 1uqt, A, G, 362; 1uqt, A, M, 363; 1uqt, A, N, 364; 1uqt, A, L, 365; 1uqt, A, V, 366; 1uqt, A, E, 369; 1uro, A, G, 222; 1uro, A, H, 223; 1uro, A, D, 264; 1vr7, A, V, 9; 1vr7, A, E, 11; 1vr7, A, H, 106; 1vr7, A, V, 108; 1xgm, A, D, 82; 1xgm, A, D, 93; 1xgm, A, H, 153; 1xgm, A, E, 280; 1xny, A, N, 80; 1xny, A, F, 109; 1xny, A, A, 112; 1xny, A, G, 142; 1xny, A, G, 143; 1xny, A, A, 144; 1xny, A, R, 145; 1xny, A, I, 146; 1xny, A, Q, 147; 1xny, A, L, 153; 1xny, A, Y, 156; 1xny, A, I, 205; 1xny, A, T, 206; 1xny, A, C, 347; 1xny, A, P, 377; 1xny, A, G, 378; 1xny, A, F, 379; 1xny, A, P, 381; 1xny, A, F, 417; 1xny, A, G, 418; 1xny, A, I, 453; 1xny, A, R, 456; 1xqd, A, K, 62; 1xqd, A, R, 64; 1xqd, A, G, 75; 1xqd, A, G, 76; 1xqd, A, Q, 78; 1xqd, A, A, 79; 1xqd, A, F, 86; 1xqd, A, V, 87; 1xqd, A, H, 94; 1xqd, A, R, 98; 1xqd, A, F, 105; 1xqd, A, I, 153; 1xqd, A, R, 174; 1xqd, A, T, 175; 1xqd, A, A, 184; 1xqd, A, N, 188; 1xqd, A, L, 235; 1xqd, A, L, 236; 1xqd, A, V, 238; 1xqd, A, A, 239; 1xqd, A, G, 240; 1xqd, A, T, 243; 1xqd, A, M, 244; 1xqd, A, M, 247; 1xqd, A, A, 289; 1xqd, A, I, 290; 1xqd, A, R, 292; 1xqd, A, N, 315; 1xqd, A, G, 344; 1xqd, A, F, 345; 1xqd, A, G, 346; 1xqd, A, H, 350; 1xqd, A, C, 352; 1xqd, A, I, 353; 1xqd, A, A, 354; 1xqd, A, A, 358; 1ycf, A, S, 2; 1ycf, A, F, 24; 1ycf, A, E, 60; 1ycf, A, H, 81; 1ycf, A, E, 83; 1ycf, A, D, 85; 1ycf, A, H, 86; 1ycf, A, H, 148; 1ycf, A, W, 149; 1ycf, A, D, 167; 1ycf, A, I, 199; 1ycf, A, H, 228; 1ycf, A, T, 261; 1ycf, A, M, 262; 1ycf, A, W, 263; 1ycf, A, L, 264; 1ycf, A, S, 265; 1ycf, A, T, 266; 1ycf, A, H, 271; 1ycf, A, D, 275; 1ycf, A, P, 312; 1ycf, A, T, 313; 1ycf, A, I, 314; 1ycf, A, N, 315; 1ycf, A, N, 316; 1ycf, A, A, 344; 1ycf, A, Y, 345; 1ycf, A, G, 346; 1ycf, A, W, 347; 1ycf, A, G, 348; 1ycf, A, G, 349; 1ycf, A, W, 376; 1ze1, A, H, 17; 1ze1, A, H, 34; 1zio, A, P, 9; 1zio, A, G, 10; 1zio, A, A, 11; 1zio, A, G, 12; 1zio, A, G, 14; 1zio, A, T, 15; 1zio, A, T, 31; 1zio, A, G, 32; 1zio, A, F, 35; 1zio, A, R, 36; 1zio, A, M, 53; 1zio, A, D, 57; 1zio, A, L, 58; 1zio, A, V, 59; 1zio, A, T, 64; 1zio, A, G, 85; 1zio, A, F, 86; 1zio, A, R, 88; 1zio, A, Q, 92; 1zio, A, R, 123; 1zio, A, L, 124; 1zio, A, C, 130; 1zio, A, C, 133; 1zio, A, T, 136; 1zio, A, Y, 137; 1zio, A, H, 138; 1zio, A, F, 141; 1zio, A, C, 150; 1zio, A, C, 153; 1zio, A, Q, 199; 1zio, A, M, 201; 2abk, A, T, 148; 2abk, A, H, 182; 2abk, A, C, 187; 2abk, A, C, 194; 2abk, A, C, 197; 2abk, A, I, 199; 2abk, A, E, 200; 2abk, A, C, 203; 2abk, A, Y, 205; 2ace, A, G, 118; 2ace, A, G, 119; 2ace, A, A, 201; 2ace, A, W, 233; 2ace, A, F, 288; 2ace, A, F, 330; 2apr, A, G, 220; 2bif, A, L, 46; 2bif, A, P, 47; 2bif, A, A, 48; 2bif, A, R, 49; 2bif, A, G, 50; 2bif, A, K, 51; 2bif, A, T, 52; 2bif, A, Y, 53; 2bif, A, G, 75; 2bif, A, R, 78; 2bif, A, F, 91; 2bif, A, L, 92; 2bif, A, P, 93; 2bif, A, R, 102; 2bif, A, L, 110; 2bif, A, D, 128; 2bif, A, A, 129; 2bif, A, T, 130; 2bif, A, T, 132; 2bif, A, T, 133; 2bif, A, R, 136; 2bif, A, M, 139; 2bif, A, S, 156; 2bif, A, N, 167; 2bif, A, Q, 170; 2bif, A, R, 193; 2bif, A, C, 196; 2bif, A, Y, 197; 2bif, A, S, 200; 2bif, A, V, 220; 2bif, A, H, 232; 2bif, A, V, 246; 2bif, A, I, 267; 2bif, A, G, 268; 2bif, A, F, 299; 2bif, A, F, 320; 2bif, A, L, 323; 2bif, A, Y, 336; 2bif, A, R, 350; 2bif, A, K, 354; 2bif, A, Y, 365; 2bif, A, Q, 370; 2bif, A, E, 373; 2bif, A, P, 374; 2bif, A, V, 375; 2bif, A, E, 378; 2bif, A, Q, 391; 2bif, A, R, 395; 2bif, A, L, 416; 2bif, A, Y, 427; 2bif, A, T, 443; 2cpu, A, N, 100; 2cpu, A, R,

158; 2cpu, A, D, 167; 2cpu, A, R, 195; 2cpu, A, H, 201; 2cpu, A, T, 254; 2cpu, A, N, 298; 2cpu, A, R, 337; 2dhn, A, A, 18; 2dhn, A, L, 72; 2dhn, A, L, 73; 2dhn, A, E, 74; 2dln, A, K, 97; 2dln, A, I, 142; 2dln, A, K, 144; 2dln, A, E, 148; 2dln, A, G, 149; 2dln, A, S, 151; 2dln, A, M, 154; 2dln, A, E, 180; 2dln, A, K, 181; 2dln, A, W, 182; 2dln, A, L, 183; 2dln, A, E, 187; 2dln, A, F, 209; 2dln, A, Y, 210; 2dln, A, K, 215; 2dln, A, D, 257; 2dln, A, M, 259; 2dln, A, L, 269; 2dln, A, E, 270; 2dln, A, N, 272; 2dln, A, P, 275; 2dln, A, S, 281; 2dln, A, L, 282; 2hdh, A, I, 21; 2hdh, A, G, 24; 2hdh, A, L, 25; 2hdh, A, D, 45; 2hdh, A, Q, 46; 2hdh, A, A, 107; 2hdh, A, I, 108; 2hdh, A, V, 109; 2hdh, A, E, 110; 2hdh, A, V, 114; 2hdh, A, K, 115; 2hdh, A, N, 135; 2hdh, A, T, 136; 2hdh, A, F, 159; 2hsa, A, K, 20; 2hsa, A, F, 21; 2hsa, A, A, 30; 2hsa, A, P, 31; 2hsa, A, M, 32; 2hsa, A, T, 33; 2hsa, A, G, 64; 2hsa, A, Q, 106; 2hsa, A, D, 232; 2hsa, A, R, 233; 2hsa, A, R, 237; 2hsa, A, T, 280; 2hsa, A, G, 288; 2hsa, A, Q, 289; 2hsa, A, E, 291; 2hsa, A, S, 319; 2hsa, A, G, 320; 2hsa, A, G, 321; 2hsa, A, Y, 341; 2hsa, A, G, 342; 2hsa, A, R, 343; 2hsa, A, Y, 364; 2hsa, A, R, 366; 2hsa, A, F, 369; 2hsa, A, Y, 370; 2jcw, A, H, 46; 2jcw, A, H, 48; 2jcw, A, H, 71; 2jcw, A, H, 80; 2jcw, A, D, 83; 2jcw, A, H, 120; 2npx, A, L, 6; 2npx, A, G, 7; 2npx, A, S, 9; 2npx, A, G, 11; 2npx, A, E, 32; 2npx, A, K, 33; 2npx, A, T, 77; 2npx, A, E, 78; 2npx, A, I, 79; 2npx, A, S, 110; 2npx, A, P, 111; 2npx, A, G, 112; 2npx, A, A, 113; 2npx, A, L, 118; 2npx, A, M, 131; 2npx, A, R, 132; 2npx, A, I, 155; 2npx, A, G, 156; 2npx, A, G, 158; 2npx, A, Y, 159; 2npx, A, I, 160; 2npx, A, E, 163; 2npx, A, D, 179; 2npx, A, I, 180; 2npx, A, L, 181; 2npx, A, Y, 188; 2npx, A, A, 241; 2npx, A, V, 242; 2npx, A, G, 243; 2npx, A, N, 247; 2npx, A, G, 280; 2npx, A, D, 281; 2npx, A, A, 297; 2npx, A, L, 298; 2npx, A, A, 299; 2npx, A, T, 300; 2npx, A, A, 302; 2npx, A, G, 328; 2oat, A, Y, 55; 2oat, A, T, 141; 2oat, A, G, 142; 2oat, A, V, 143; 2oat, A, W, 178; 2oat, A, G, 179; 2oat, A, R, 180; 2oat, A, E, 230; 2oat, A, E, 235; 2oat, A, I, 265; 2oat, A, Q, 266; 2oat, A, G, 320; 2oat, A, S, 321; 2oat, A, T, 322; 2pda, A, Y, 28; 2pda, A, P, 29; 2pda, A, I, 30; 2pda, A, K, 459; 2pda, A, P, 682; 2pda, A, W, 684; 2pda, A, C, 689; 2pda, A, I, 690; 2pda, A, C, 692; 2pda, A, N, 693; 2pda, A, C, 695; 2pda, A, C, 699; 2pda, A, P, 700; 2pda, A, A, 703; 2pda, A, I, 704; 2pda, A, I, 738; 2pda, A, C, 745; 2pda, A, M, 746; 2pda, A, G, 747; 2pda, A, C, 748; 2pda, A, G, 749; 2pda, A, N, 750; 2pda, A, C, 751; 2pda, A, C, 755; 2pda, A, P, 756; 2pda, A, P, 757; 2pda, A, A, 761; 2pda, A, L, 762; 2pda, A, C, 812; 2pda, A, C, 815; 2pda, A, E, 817; 2pda, A, G, 839; 2pda, A, C, 840; 2pda, A, I, 843; 2pda, A, F, 869; 2pda, A, E, 870; 2pda, A, G, 962; 2pda, A, D, 963; 2pda, A, G, 964; 2pda, A, W, 965; 2pda, A, I, 969; 2pda, A, D, 983; 2pda, A, V, 984; 2pda, A, N, 985; 2pda, A, T, 991; 2pda, A, V, 993; 2pda, A, Y, 994; 2pda, A, S, 995; 2pda, A, T, 997; 2pda, A, A, 1056; 2pda, A, E, 1057; 2pda, A, F, 1059; 2pda, A, G, 1061; 2pda, A, S, 1063; 2pda, A, C, 1071; 2pda, A, I, 1072; 2pda, A, M, 1202; 2pda, A, M, 1203; 2pfl, A, A, 652; 2pfl, A, L, 654; 2pfl, A, E, 700; 2pfl, A, G, 701; 2pgd, A, N, 102; 2pgd, A, Y, 191; 2pgd, A, G, 211; 2pgd, A, H, 212; 2pgd, A, H, 248; 2pgd, A, Q, 259; 2pgd, A, K, 260; 2pgd, A, R, 287; 2pia, A, R, 55; 2pia, A, T, 56; 2pia, A, Y, 57; 2pia, A, A, 72; 2pia, A, V, 73; 2pia, A, K, 74; 2pia, A, D, 76; 2pia, A, G, 79; 2pia, A, R, 80; 2pia, A, G, 81; 2pia, A, G, 82; 2pia, A, S, 83; 2pia, A, I, 121; 2pia, A, T, 124; 2pia, A, S, 224; 2pia, A, F, 225; 2pia, A, S, 270; 2pia, A, S, 271; 2pia, A, C, 272; 2pia, A, E, 273; 2pia, A, S, 274; 2pia, A, G, 275; 2pia, A, T, 276; 2pia, A, C, 277; 2pia, A, G, 278; 2pia, A, C, 280; 2pia, A, M, 306; 2pia, A, C, 308; 2tdt, A, R, 104; 2tdt, A, M, 124; 2tdt, A, M, 139; 2tdt, A, S, 159; 2tdt, A, G, 160; 2tdt, A, F, 183; 2tdt, A, G, 185; 2tdt, A, A, 186; 2tdt, A, S, 203; 2tdt, A, M, 204; 2tdt, A, R, 217; 2tdt, A, V, 234; 2tdt, A, V, 253; 2tdt, A, K, 254; 2tdt, A, K, 259; 2tdt, A, T, 260; 2tdt, A, K, 263; 2tdt, A, L, 270; 2tps, A, Y, 29; 2tps, A, I, 31; 2tps, A, Q, 57; 2tps, A, K, 61; 2tps, A, N, 92; 2tps, A, D, 93; 2tps, A, H, 107; 2tps, A, G, 109; 2tps, A, D, 112; 2tps, A, G, 149; 2tps, A, T, 156; 2tps, A, T, 158; 2tps, A, I, 186; 2tps, A, G, 188; 2tps, A, M, 207; 2tps, A, I, 208; 2tps, A, S, 209; 2ypn, A, L, 15; 2ypn, A, Q, 19; 2ypn, A, S, 81; 2ypn, A, K, 83; 2ypn, A, T, 127; 2ypn, A, S, 128; 2ypn, A, S, 129; 2ypn, A, L, 148; 2ypn, A, L, 169; 2ypn, A, A, 170; 2ypn, A, A, 195; 2ypn, A, Q, 198; 2ypn, A, G, 199; 3mdd, A, Y, 133; 3mdd, A, V, 135; 3mdd, A, T, 136; 3mdd, A, G, 141; 3mdd, A, S, 142; 3mdd, A, W, 166; 3mdd, A, I, 167;

3mdd, A, T, 168; 3mdd, A, N, 214; 3mdd, A, R, 281; 3mdd, A, T, 283; 3mdd, A, F, 284; 3mdd, A, L, 288; 3mdd, A, H, 291; 3mdd, A, I, 294; 3mdd, A, Q, 349; 3mdd, A, V, 350; 3mdd, A, G, 352; 3mdd, A, G, 353; 3mdd, A, I, 371; 3mdd, A, I, 374; 3mdd, A, T, 378; 3mdd, A, Q, 380; 3mdd, A, I, 381; 3mdd, A, I, 384; 3r1r, A, K, 9; 3r1r, A, E, 15; 3r1r, A, R, 16; 3r1r, A, I, 17; 3r1r, A, N, 18; 3r1r, A, K, 21; 3r1r, A, I, 22; 3r1r, A, V, 25; 3r1r, A, T, 55; 3r1r, A, H, 59; 3r1r, A, K, 91; 3r1r, A, A, 559; 3r1r, A, K, 560; 3r1r, A, E, 561; 3r1r, A, Q, 562; 3r1r, A, G, 563; 3r1r, A, A, 564; 3r1r, A, F, 568; 3r1r, A, H, 609; 4mdh, A, G, 10; 4mdh, A, A, 12; 4mdh, A, G, 13; 4mdh, A, Q, 14; 4mdh, A, I, 15; 4mdh, A, D, 41; 4mdh, A, I, 42; 4mdh, A, V, 86; 4mdh, A, G, 87; 4mdh, A, S, 88; 4mdh, A, M, 89; 4mdh, A, P, 90; 4mdh, A, R, 91; 4mdh, A, I, 107; 4mdh, A, V, 128; 4mdh, A, G, 129; 4mdh, A, N, 130; 4mdh, A, A, 132; 4mdh, A, L, 154; 4mdh, A, L, 157; 4mdh, A, R, 161; 4mdh, A, G, 230; 4mdh, A, S, 241; 4mdh, A, A, 245; 5cpa, A, H, 69; 5cpa, A, E, 72; 5cpa, A, H, 196; 5eat, A, D, 301; 5eat, A, D, 305; 5eat, A, Y, 376; 5eat, A, E, 379; 5eat, A, Y, 404; 5eat, A, C, 440; 5eat, A, D, 445; 5eat, A, T, 448; 5eat, A, E, 452; 5eat, A, T, 528; 5enl, A, D, 246; 5enl, A, E, 295; 5enl, A, D, 320; 5enl, A, S, 372; 5enl, A, R, 374; 5enl, A, S, 375; 5enl, A, K, 396; 5fit, A, F, 5; 5fit, A, H, 8; 5fit, A, L, 25; 5fit, A, V, 26; 5fit, A, N, 27; 5fit, A, R, 28; 5fit, A, K, 29; 5fit, A, H, 35; 5fit, A, L, 37; 5fit, A, G, 89; 5fit, A, Q, 90; 5fit, A, T, 91; 5fit, A, V, 92; 5fit, A, H, 98; 5rsa, A, K, 1; 5rsa, A, E, 2; 5rsa, A, T, 3; 5rsa, A, A, 4; 5rsa, A, A, 5; 5rsa, A, A, 6; 5rsa, A, K, 7; 5rsa, A, F, 8; 5rsa, A, E, 9; 5rsa, A, R, 10; 5rsa, A, Q, 11; 5rsa, A, M, 13; 5rsa, A, D, 14; 5rsa, A, S, 15; 5rsa, A, S, 16; 5rsa, A, T, 17; 5rsa, A, S, 18; 5rsa, A, A, 19; 5rsa, A, A, 20; 5rsa, A, S, 22; 5rsa, A, S, 23; 5rsa, A, N, 24; 5rsa, A, Y, 25; 5rsa, A, C, 26; 5rsa, A, N, 27; 5rsa, A, Q, 28; 5rsa, A, M, 29; 5rsa, A, K, 31; 5rsa, A, S, 32; 5rsa, A, R, 33; 5rsa, A, N, 34; 5rsa, A, L, 35; 5rsa, A, T, 36; 5rsa, A, K, 37; 5rsa, A, D, 38; 5rsa, A, R, 39; 5rsa, A, C, 40; 5rsa, A, P, 42; 5rsa, A, V, 43; 5rsa, A, N, 44; 5rsa, A, T, 45; 5rsa, A, H, 48; 5rsa, A, E, 49; 5rsa, A, S, 50; 5rsa, A, L, 51; 5rsa, A, A, 52; 5rsa, A, D, 53; 5rsa, A, Q, 55; 5rsa, A, A, 56; 5rsa, A, C, 58; 5rsa, A, S, 59; 5rsa, A, Q, 60; 5rsa, A, A, 64; 5rsa, A, C, 65; 5rsa, A, K, 66; 5rsa, A, N, 67; 5rsa, A, G, 68; 5rsa, A, Q, 69; 5rsa, A, N, 71; 5rsa, A, Y, 73; 5rsa, A, Q, 74; 5rsa, A, S, 75; 5rsa, A, Y, 76; 5rsa, A, S, 77; 5rsa, A, M, 79; 5rsa, A, S, 80; 5rsa, A, D, 83; 5rsa, A, R, 85; 5rsa, A, E, 86; 5rsa, A, S, 89; 5rsa, A, S, 90; 5rsa, A, K, 91; 5rsa, A, Y, 92; 5rsa, A, P, 93; 5rsa, A, N, 94; 5rsa, A, C, 95; 5rsa, A, Y, 97; 5rsa, A, K, 98; 5rsa, A, T, 99; 5rsa, A, T, 100; 5rsa, A, Q, 101; 5rsa, A, N, 103; 5rsa, A, H, 105; 5rsa, A, A, 109; 5rsa, A, C, 110; 5rsa, A, E, 111; 5rsa, A, G, 112; 5rsa, A, N, 113; 5rsa, A, P, 114; 5rsa, A, Y, 115; 5rsa, A, V, 116; 5rsa, A, P, 117; 5rsa, A, V, 118; 5rsa, A, F, 120; 5rsa, A, D, 121; 5rsa, A, A, 122; 5rsa, A, S, 123; 5rsa, A, V, 124; 7atj, A, N, 13; 7atj, A, N, 16; 7atj, A, I, 17; 7atj, A, D, 20; 7atj, A, R, 31; 7atj, A, A, 34; 7atj, A, S, 35; 7atj, A, L, 37; 7atj, A, F, 41; 7atj, A, D, 43; 7atj, A, V, 46; 7atj, A, G, 48; 7atj, A, D, 50; 7atj, A, S, 52; 7atj, A, F, 68; 7atj, A, G, 69; 7atj, A, S, 73; 7atj, A, R, 75; 7atj, A, P, 139; 7atj, A, A, 140; 7atj, A, P, 141; 7atj, A, F, 142; 7atj, A, L, 148; 7atj, A, F, 152; 7atj, A, L, 163; 7atj, A, L, 166; 7atj, A, S, 167; 7atj, A, G, 169; 7atj, A, H, 170; 7atj, A, T, 171; 7atj, A, F, 172; 7atj, A, G, 173; 7atj, A, K, 174; 7atj, A, N, 175; 7atj, A, Q, 176; 7atj, A, F, 179; 7atj, A, F, 221; 7atj, A, D, 222; 7atj, A, T, 225; 7atj, A, I, 228; 7atj, A, D, 230; 7atj, A, I, 244; 7atj, A, S, 246;

Table S4

The dataset *STRUC_sites*. For each site, PDB-ID, chain, amino acid, and position as deduced from the PDB-file are listed. Entries are separated by a ";". Listed are buried residues with a $cons_{ident}(k)$-values ≥ 1.0.

1a0k, A, A, 23; 1a0k, A, A, 34; 1a0k, A, D, 8; 1a0k, A, E, 46; 1a0k, A, E, 120; 1a0k, A, G, 27; 1a0k, A, G, 64; 1a0k, A, G, 91; 1a0k, A, I, 25; 1a0k, A, K, 86; 1a0k, A, L, 11; 1a0k, A, L, 126; 1a0k, A, S, 36; 1a0k, A, T, 97; 1a0k, A, V, 119; 1a0k, A, W, 3; 1a0k, A, W, 33; 1a0k, A, Y, 6; 1a0k, A, Y, 106; 1a0k, A, Y, 125; 1a44, A, A, 72; 1a44, A, A, 164; 1a44, A, D, 69; 1a44, A, D, 71; 1a44, A, E, 82; 1a44, A, F, 86; 1a44, A, F, 148; 1a44, A, F, 153; 1a44, A, G, 37; 1a44, A, G, 99; 1a44, A, G, 115; 1a44, A, H, 117; 1a44, A, L, 65; 1a44, A, L, 67; 1a44, A, L, 159; 1a44, A, M, 91; 1a44, A, P, 42; 1a44, A, P, 49; 1a44, A, P, 70; 1a44, A, P, 73; 1a44, A, P, 78; 1a44, A, P, 110; 1a44, A, P, 162; 1a44, A, Q, 126; 1a44, A, R, 81; 1a44, A, R, 118; 1a44, A, R, 145; 1a44, A, S, 74; 1a44, A, T, 64; 1a44, A, V, 88; 1a44, A, V, 176; 1a44, A, Y, 28; 1a44, A, Y, 63; 1a44, A, Y, 105; 1a44, A, Y, 175; 1a45, A, D, 108; 1a45, A, E, 7; 1a45, A, E, 94; 1a45, A, E, 104; 1a45, A, F, 11; 1a45, A, G, 1; 1a45, A, G, 13; 1a45, A, G, 40; 1a45, A, G, 52; 1a45, A, G, 60; 1a45, A, G, 100; 1a45, A, G, 129; 1a45, A, G, 141; 1a45, A, G, 149; 1a45, A, H, 22; 1a45, A, Q, 54; 1a45, A, R, 79; 1a45, A, R, 168; 1a45, A, R, 169; 1a45, A, S, 34; 1a45, A, S, 77; 1a45, A, S, 123; 1a45, A, S, 166; 1a45, A, V, 37; 1a45, A, V, 126; 1a45, A, W, 131; 1a45, A, Y, 45; 1a45, A, Y, 62; 1a45, A, Y, 134; 1a45, A, Y, 139; 1a45, A, Y, 151; 1a62, A, D, 78; 1a62, A, D, 95; 1a62, A, E, 56; 1a62, A, E, 108; 1a62, A, F, 64; 1a62, A, F, 89; 1a62, A, G, 53; 1a62, A, G, 61; 1a62, A, G, 63; 1a62, A, G, 94; 1a62, A, G, 99; 1a62, A, I, 86; 1a62, A, L, 55; 1a62, A, L, 65; 1a62, A, L, 91; 1a62, A, L, 113; 1a62, A, P, 104; 1a62, A, P, 124; 1a62, A, Q, 85; 1a62, A, R, 66; 1a62, A, R, 102; 1a62, A, S, 82; 1a62, A, Y, 72; 1a62, A, Y, 80; 1a7g, E, F, 353; 1a7g, E, G, 300; 1a7g, E, G, 368; 1a7g, E, L, 305; 1a7g, E, P, 294; 1a7g, E, P, 360; 1a7g, E, Q, 349; 1a7g, E, R, 350; 1a7g, E, S, 323; 1a7g, E, V, 357; 1a8a, A, A, 25; 1a8a, A, A, 91; 1a8a, A, A, 97; 1a8a, A, A, 174; 1a8a, A, A, 180; 1a8a, A, A, 250; 1a8a, A, D, 18; 1a8a, A, D, 173; 1a8a, A, D, 188; 1a8a, A, D, 278; 1a8a, A, E, 110; 1a8a, A, E, 276; 1a8a, A, F, 13; 1a8a, A, F, 55; 1a8a, A, G, 73; 1a8a, A, I, 51; 1a8a, A, I, 111; 1a8a, A, I, 195; 1a8a, A, I, 282; 1a8a, A, L, 22; 1a8a, A, L, 40; 1a8a, A, L, 63; 1a8a, A, L, 108; 1a8a, A, L, 135; 1a8a, A, L, 151; 1a8a, A, L, 154; 1a8a, A, L, 177; 1a8a, A, L, 204; 1a8a, A, L, 249; 1a8a, A, L, 253; 1a8a, A, L, 267; 1a8a, A, L, 294; 1a8a, A, L, 310; 1a8a, A, L, 313; 1a8a, A, M, 67; 1a8a, A, Q, 47; 1a8a, A, R, 43; 1a8a, A, R, 48; 1a8a, A, R, 115; 1a8a, A, R, 159; 1a8a, A, R, 199; 1a8a, A, R, 269; 1a8a, A, R, 274; 1a8a, A, S, 200; 1a8a, A, T, 6; 1a8a, A, T, 262; 1a8a, A, T, 302; 1a8a, A, Y, 127; 1a8a, A, Y, 211; 1a8a, A, Y, 306; 1a99, A, A, 136; 1a99, A, A, 210; 1a99, A, A, 251; 1a99, A, A, 286; 1a99, A, A, 292; 1a99, A, A, 306; 1a99, A, C, 175; 1a99, A, D, 80; 1a99, A, D, 102; 1a99, A, D, 124; 1a99, A, D, 181; 1a99, A, E, 66; 1a99, A, E, 108; 1a99, A, E, 271; 1a99, A, F, 49; 1a99, A, F, 79; 1a99, A, F, 129; 1a99, A, F, 224; 1a99, A, F, 295; 1a99, A, G, 75; 1a99, A, G, 78; 1a99, A, G, 139; 1a99, A, G, 152; 1a99, A, G, 176; 1a99, A, G, 236; 1a99, A, G, 246; 1a99, A, G, 272; 1a99, A, I, 41; 1a99, A, I, 55; 1a99, A, K, 367; 1a99, A, L, 101; 1a99, A, L, 151; 1a99, A, L, 191; 1a99, A, L, 214; 1a99, A, L, 296; 1a99, A, M, 282; 1a99, A, N, 36; 1a99, A, N, 65; 1a99, A, N, 127; 1a99, A, N, 143; 1a99, A, N, 318; 1a99, A, P, 84; 1a99, A, P, 132; 1a99, A, P, 166; 1a99, A, P, 269; 1a99, A, P, 283; 1a99, A, P, 302; 1a99, A, P, 337; 1a99, A, S, 64; 1a99, A, S, 159; 1a99, A, T, 53; 1a99, A, V, 57; 1a99, A, W, 135; 1a99, A, W, 160; 1a99, A, Y, 35; 1a99, A, Y, 59; 1a99, A, Y, 133; 1ahq, A, A, 63; 1ahq, A, A, 117; 1ahq, A, C, 60; 1ahq, A, D, 66; 1ahq, A, E, 122; 1ahq, A, F, 13; 1ahq, A, F, 51; 1ahq, A, F, 81; 1ahq, A, G, 110; 1ahq, A, I, 79; 1ahq, A, K, 78; 1ahq, A, K, 94; 1ahq, A, L, 55; 1ahq, A, L, 108; 1ahq, A, P, 86; 1ahq,

A, R, 61; 1ahq, A, T, 100; 1ahq, A, V, 5; 1ahq, A, V, 38; 1ahq, A, W, 84; 1ahq, A, Y, 48; 1ahq, A, Y, 62; 1ahq, A, Y, 97; 1aqd, A, C, 107; 1aqd, A, C, 163; 1aqd, A, D, 35; 1aqd, A, D, 142; 1aqd, A, D, 159; 1aqd, A, E, 30; 1aqd, A, E, 166; 1aqd, A, F, 32; 1aqd, A, F, 54; 1aqd, A, F, 145; 1aqd, A, F, 153; 1aqd, A, G, 20; 1aqd, A, G, 169; 1aqd, A, H, 167; 1aqd, A, I, 106; 1aqd, A, L, 60; 1aqd, A, L, 70; 1aqd, A, L, 99; 1aqd, A, L, 105; 1aqd, A, L, 151; 1aqd, A, L, 170; 1aqd, A, N, 103; 1aqd, A, N, 124; 1aqd, A, P, 87; 1aqd, A, P, 114; 1aqd, A, P, 115; 1aqd, A, P, 155; 1aqd, A, V, 34; 1aqd, A, V, 97; 1aqd, A, V, 116; 1aqd, A, V, 165; 1aqd, A, W, 43; 1aqd, A, W, 121; 1aqd, A, W, 178; 1aqd, A, Y, 150; 1aqd, A, Y, 161; 1ar0, A, D, 117; 1ar0, A, F, 14; 1ar0, A, F, 22; 1ar0, A, F, 61; 1ar0, A, F, 103; 1ar0, A, F, 119; 1ar0, A, G, 48; 1ar0, A, G, 87; 1ar0, A, I, 52; 1ar0, A, K, 42; 1ar0, A, K, 55; 1ar0, A, L, 29; 1ar0, A, L, 59; 1ar0, A, L, 89; 1ar0, A, L, 105; 1ar0, A, L, 121; 1ar0, A, N, 116; 1ar0, A, Q, 101; 1ar0, A, R, 26; 1ar0, A, S, 37; 1ar0, A, T, 40; 1ar0, A, V, 85; 1ar0, A, Y, 18; 1ar0, A, Y, 19; 1ar0, A, Y, 33; 1atg, A, A, 48; 1atg, A, A, 79; 1atg, A, A, 109; 1atg, A, A, 121; 1atg, A, A, 144; 1atg, A, A, 165; 1atg, A, A, 167; 1atg, A, A, 207; 1atg, A, D, 57; 1atg, A, F, 22; 1atg, A, F, 53; 1atg, A, G, 35; 1atg, A, G, 38; 1atg, A, G, 81; 1atg, A, G, 119; 1atg, A, G, 122; 1atg, A, G, 162; 1atg, A, G, 227; 1atg, A, I, 44; 1atg, A, L, 64; 1atg, A, L, 83; 1atg, A, L, 85; 1atg, A, L, 100; 1atg, A, L, 126; 1atg, A, N, 112; 1atg, A, P, 113; 1atg, A, P, 184; 1atg, A, Q, 138; 1atg, A, Q, 193; 1atg, A, S, 111; 1atg, A, Y, 78; 1aue, A, A, 2035; 1aue, A, A, 2082; 1aue, A, A, 2101; 1aue, A, E, 2068; 1aue, A, F, 2071; 1aue, A, L, 2023; 1aue, A, L, 2032; 1aue, A, L, 2052; 1aue, A, L, 2055; 1aue, A, L, 2079; 1aue, A, M, 2048; 1aue, A, S, 2036; 1aue, A, V, 2108; 1aue, A, W, 2024; 1aue, A, W, 2028; 1aue, A, W, 2102; 1aue, A, Y, 2105; 1aun, A, C, 10; 1aun, A, C, 52; 1aun, A, C, 62; 1aun, A, C, 67; 1aun, A, C, 73; 1aun, A, C, 126; 1aun, A, C, 134; 1aun, A, C, 144; 1aun, A, C, 148; 1aun, A, C, 157; 1aun, A, C, 158; 1aun, A, C, 163; 1aun, A, C, 176; 1aun, A, C, 193; 1aun, A, C, 205; 1aun, A, D, 66; 1aun, A, D, 95; 1aun, A, D, 98; 1aun, A, D, 103; 1aun, A, D, 185; 1aun, A, E, 85; 1aun, A, F, 172; 1aun, A, F, 204; 1aun, A, G, 58; 1aun, A, G, 65; 1aun, A, G, 77; 1aun, A, G, 104; 1aun, A, K, 173; 1aun, A, L, 27; 1aun, A, L, 83; 1aun, A, L, 138; 1aun, A, N, 8; 1aun, A, N, 106; 1aun, A, P, 80; 1aun, A, P, 135; 1aun, A, P, 206; 1aun, A, R, 45; 1aun, A, R, 49; 1aun, A, S, 100; 1aun, A, T, 64; 1aun, A, W, 15; 1aun, A, W, 47; 1aun, A, Y, 180; 1aun, A, Y, 182; 1aun, A, Y, 200; 1axi, B, C, 38; 1axi, B, C, 48; 1axi, B, C, 83; 1axi, B, C, 94; 1axi, B, D, 164; 1axi, B, E, 42; 1axi, B, E, 44; 1axi, B, E, 61; 1axi, B, E, 82; 1axi, B, F, 35; 1axi, B, F, 46; 1axi, B, F, 67; 1axi, B, F, 96; 1axi, B, G, 62; 1axi, B, H, 49; 1axi, B, I, 64; 1axi, B, I, 128; 1axi, B, L, 66; 1axi, B, L, 202; 1axi, B, P, 33; 1axi, B, P, 41; 1axi, B, P, 84; 1axi, B, P, 131; 1axi, B, P, 133; 1axi, B, P, 134; 1axi, B, P, 160; 1axi, B, Q, 65; 1axi, B, R, 39; 1axi, B, R, 43; 1axi, B, R, 70; 1axi, B, R, 213; 1axi, B, S, 40; 1axi, B, S, 47; 1axi, B, S, 226; 1axi, B, T, 36; 1axi, B, T, 45; 1axi, B, T, 69; 1axi, B, T, 101; 1axi, B, V, 129; 1axi, B, W, 50; 1axi, B, W, 157; 1axi, B, W, 169; 1axi, B, W, 186; 1axi, B, Y, 68; 1axi, B, Y, 86; 1axi, B, Y, 107; 1axi, B, Y, 174; 1ayf, A, A, 81; 1ayf, A, E, 74; 1ayf, A, H, 56; 1ayf, A, L, 57; 1ayf, A, L, 78; 1ayf, A, L, 90; 1ayf, A, P, 108; 1ayf, A, Q, 93; 1ayf, A, R, 89; 1ayf, A, S, 88; 1ayf, A, T, 54; 1aym, 1, S, 23; 1aym, 1, T, 39; 1aym, 1, T, 53; 1aym, 1, F, 70; 1aym, 1, W, 96; 1aym, 1, R, 107; 1aym, 1, K, 109; 1aym, 1, E, 111; 1aym, 1, F, 113; 1aym, 1, T, 114; 1aym, 1, Y, 115; 1aym, 1, R, 117; 1aym, 1, F, 118; 1aym, 1, D, 119; 1aym, 1, E, 121; 1aym, 1, T, 123; 1aym, 1, V, 125; 1aym, 1, Q, 141; 1aym, 1, M, 143; 1aym, 1, Y, 144; 1aym, 1, P, 152; 1aym, 1, W, 160; 1aym, 1, N, 165; 1aym, 1, S, 167; 1aym, 1, F, 183; 1aym, 1, A, 189; 1aym, 1, F, 193; 1aym, 1, Y, 194; 1aym, 1, G, 196; 1aym, 1, G, 207; 1aym, 1, G, 215; 1aym, 1, R, 220; 1aym, 1, V, 222; 1aym, 1, R, 235; 1aym, 1, Y, 237; 1aym, 1, K, 239; 1aym, 1, K, 241; 1aym, 1, W, 246; 1aym, 1, R, 249; 1aym, 1, R, 252; 1aym, 1, Y, 256; 1aym, 2, S, 10; 1aym, 2, R, 12; 1aym, 2, G, 19; 1aym, 2, S, 21; 1aym, 2, T, 22; 1aym, 2, Y, 35; 1aym, 2, P, 39; 1aym, 2, A, 47; 1aym, 2, D, 51; 1aym, 2, P, 56; 1aym, 2, R, 62; 1aym, 2, F, 63; 1aym, 2, Y, 64; 1aym, 2, W, 71; 1aym, 2, G, 77; 1aym, 2, K, 81; 1aym, 2, P, 83; 1aym, 2, L, 86; 1aym, 2, F, 92; 1aym, 2, G, 93; 1aym, 2, H, 99; 1aym, 2, R, 103; 1aym, 2, G, 105; 1aym, 2, V, 110; 1aym, 2, Q, 111; 1aym, 2, H, 118; 1aym, 2, G,

120; 1aym, 2, L, 122; 1aym, 2, V, 124; 1aym, 2, P, 128; 1aym, 2, P, 186; 1aym, 2, H, 187; 1aym, 2, Q, 188; 1aym, 2, I, 190; 1aym, 2, N, 191; 1aym, 2, L, 192; 1aym, 2, N, 195; 1aym, 2, A, 198; 1aym, 2, P, 203; 1aym, 2, Y, 204; 1aym, 2, D, 211; 1aym, 2, H, 216; 1aym, 2, L, 221; 1aym, 2, L, 229; 1aym, 2, T, 241; 1aym, 2, P, 246; 1aym, 2, G, 253; 1aym, 2, R, 255; 1aym, 3, G, 9; 1aym, 3, D, 17; 1aym, 3, V, 40; 1aym, 3, E, 45; 1aym, 3, N, 56; 1aym, 3, T, 97; 1aym, 3, G, 100; 1aym, 3, W, 109; 1aym, 3, G, 111; 1aym, 3, S, 112; 1aym, 3, F, 117; 1aym, 3, F, 119; 1aym, 3, G, 121; 1aym, 3, K, 128; 1aym, 3, L, 130; 1aym, 3, Y, 133; 1aym, 3, P, 135; 1aym, 3, P, 136; 1aym, 3, P, 141; 1aym, 3, R, 144; 1aym, 3, A, 147; 1aym, 3, M, 148; 1aym, 3, G, 150; 1aym, 3, T, 151; 1aym, 3, H, 152; 1aym, 3, W, 155; 1aym, 3, P, 168; 1aym, 3, W, 169; 1aym, 3, S, 171; 1aym, 3, R, 176; 1aym, 3, G, 187; 1aym, 3, Y, 193; 1aym, 3, Q, 194; 1aym, 3, P, 200; 1aym, 3, F, 219; 1b09, A, C, 36; 1b09, A, C, 97; 1b09, A, D, 155; 1b09, A, D, 163; 1b09, A, F, 11; 1b09, A, F, 52; 1b09, A, F, 146; 1b09, A, G, 105; 1b09, A, G, 113; 1b09, A, G, 136; 1b09, A, G, 144; 1b09, A, G, 154; 1b09, A, H, 95; 1b09, A, I, 156; 1b09, A, L, 22; 1b09, A, L, 30; 1b09, A, L, 135; 1b09, A, L, 152; 1b09, A, L, 166; 1b09, A, M, 161; 1b09, A, N, 158; 1b09, A, N, 183; 1b09, A, P, 12; 1b09, A, Q, 137; 1b09, A, R, 47; 1b09, A, S, 102; 1b09, A, S, 151; 1b09, A, T, 34; 1b09, A, V, 20; 1b09, A, V, 165; 1b09, A, V, 198; 1b09, A, W, 100; 1b09, A, W, 110; 1b09, A, W, 162; 1b09, A, W, 187; 1b09, A, W, 205; 1b09, A, Y, 192; 1b3a, A, A, 51; 1b3a, A, C, 10; 1b3a, A, C, 11; 1b3a, A, C, 34; 1b3a, A, C, 50; 1b3a, A, F, 41; 1b3a, A, L, 65; 1b3a, A, P, 53; 1b3a, A, T, 30; 1b3a, A, V, 39; 1b3a, A, V, 58; 1b3a, A, W, 57; 1b3a, A, Y, 27; 1b67, A, A, 25; 1b67, A, A, 44; 1b67, A, A, 51; 1b67, A, A, 64; 1b67, A, D, 60; 1b67, A, E, 34; 1b8z, A, A, 84; 1b8z, A, F, 50; 1b8z, A, F, 79; 1b8z, A, G, 46; 1b8z, A, G, 48; 1b8z, A, G, 82; 1b8z, A, L, 6; 1b8z, A, L, 85; 1b8z, A, M, 1; 1b8z, A, P, 81; 1bd8, A, A, 15; 1bd8, A, A, 16; 1bd8, A, A, 65; 1bd8, A, A, 81; 1bd8, A, A, 82; 1bd8, A, A, 98; 1bd8, A, A, 114; 1bd8, A, A, 146; 1bd8, A, D, 71; 1bd8, A, G, 19; 1bd8, A, G, 52; 1bd8, A, G, 74; 1bd8, A, G, 85; 1bd8, A, G, 107; 1bd8, A, G, 118; 1bd8, A, G, 139; 1bd8, A, H, 79; 1bd8, A, L, 12; 1bd8, A, L, 27; 1bd8, A, L, 28; 1bd8, A, L, 46; 1bd8, A, L, 60; 1bd8, A, L, 61; 1bd8, A, L, 93; 1bd8, A, L, 126; 1bd8, A, L, 158; 1bd8, A, P, 77; 1bd8, A, P, 110; 1bd8, A, P, 142; 1bd8, A, T, 44; 1bd8, A, T, 141; 1bd8, A, V, 24; 1bd8, A, V, 78; 1bft, A, A, 249; 1bft, A, F, 228; 1bft, A, F, 239; 1bft, A, F, 286; 1bft, A, G, 208; 1bft, A, I, 196; 1bft, A, I, 224; 1bft, A, I, 250; 1bft, A, L, 194; 1bft, A, L, 215; 1bft, A, L, 272; 1bft, A, P, 255; 1bft, A, P, 290; 1bft, A, R, 274; 1bft, A, S, 281; 1bft, A, T, 254; 1bft, A, V, 219; 1bft, A, V, 244; 1bft, A, V, 268; 1bft, A, W, 233; 1bft, A, Y, 257; 1bhd, A, A, 184; 1bhd, A, A, 187; 1bhd, A, A, 214; 1bhd, A, A, 218; 1bhd, A, F, 175; 1bhd, A, F, 185; 1bhd, A, F, 215; 1bhd, A, G, 182; 1bhd, A, H, 190; 1bhd, A, L, 156; 1bhd, A, L, 157; 1bhd, A, L, 228; 1bhd, A, P, 238; 1bhd, A, R, 210; 1bhd, A, S, 178; 1bhd, A, V, 170; 1bhd, A, W, 159; 1bhd, A, W, 179; 1bhd, A, Y, 167; 1bhd, A, Y, 246; 1bkb, A, A, 46; 1bkb, A, C, 30; 1bkb, A, D, 95; 1bkb, A, F, 56; 1bkb, A, G, 20; 1bkb, A, G, 41; 1bkb, A, G, 44; 1bkb, A, I, 134; 1bkb, A, K, 47; 1bkb, A, K, 133; 1bkb, A, P, 29; 1bkb, A, P, 66; 1bkb, A, P, 74; 1bv1, A, A, 21; 1bv1, A, D, 25; 1bv1, A, D, 75; 1bv1, A, E, 148; 1bv1, A, G, 46; 1bv1, A, G, 48; 1bv1, A, G, 49; 1bv1, A, G, 51; 1bv1, A, G, 88; 1bv1, A, G, 111; 1bv1, A, I, 53; 1bv1, A, K, 54; 1bv1, A, K, 103; 1bv1, A, K, 115; 1bv1, A, K, 137; 1bv1, A, P, 31; 1bv1, A, V, 2; 1bv1, A, Y, 83; 1bv1, A, Y, 150; 1bv1, A, Y, 158; 1c5k, A, A, 216; 1c5k, A, A, 249; 1c5k, A, A, 260; 1c5k, A, D, 62; 1c5k, A, D, 121; 1c5k, A, D, 151; 1c5k, A, D, 187; 1c5k, A, D, 189; 1c5k, A, D, 255; 1c5k, A, D, 299; 1c5k, A, D, 378; 1c5k, A, E, 293; 1c5k, A, F, 43; 1c5k, A, F, 69; 1c5k, A, F, 164; 1c5k, A, F, 220; 1c5k, A, F, 305; 1c5k, A, G, 39; 1c5k, A, G, 67; 1c5k, A, G, 103; 1c5k, A, G, 162; 1c5k, A, G, 190; 1c5k, A, G, 212; 1c5k, A, G, 256; 1c5k, A, G, 332; 1c5k, A, G, 344; 1c5k, A, G, 387; 1c5k, A, G, 409; 1c5k, A, G, 420; 1c5k, A, H, 147; 1c5k, A, I, 38; 1c5k, A, L, 63; 1c5k, A, L, 119; 1c5k, A, L, 157; 1c5k, A, L, 183; 1c5k, A, L, 263; 1c5k, A, L, 372; 1c5k, A, L, 402; 1c5k, A, L, 415; 1c5k, A, N, 247; 1c5k, A, N, 335; 1c5k, A, N, 386; 1c5k, A, P, 202; 1c5k, A, P, 206; 1c5k, A, P, 210; 1c5k, A, P, 250; 1c5k, A, P, 254; 1c5k, A, P, 294; 1c5k, A, P, 298; 1c5k, A, P, 313; 1c5k, A, P, 381; 1c5k, A, P, 385; 1c5k, A, P, 425; 1c5k, A,

P, 429; 1c5k, A, Q, 314; 1c5k, A, R, 143; 1c5k, A, R, 327; 1c5k, A, S, 66; 1c5k, A, S, 199; 1c5k, A, S, 205; 1c5k, A, S, 209; 1c5k, A, S, 253; 1c5k, A, S, 300; 1c5k, A, S, 307; 1c5k, A, T, 158; 1c5k, A, T, 166; 1c5k, A, T, 285; 1c5k, A, T, 292; 1c5k, A, V, 171; 1c5k, A, V, 218; 1c5k, A, V, 237; 1c5k, A, W, 92; 1c5k, A, W, 208; 1c5k, A, W, 427; 1c5k, A, Y, 170; 1c5k, A, Y, 181; 1c5k, A, Y, 217; 1c5k, A, Y, 272; 1c5k, A, Y, 316; 1c5k, A, Y, 392; 1cc7, A, A, 21; 1cc7, A, C, 15; 1cc7, A, C, 18; 1cc7, A, K, 65; 1cc7, A, M, 13; 1cc7, A, T, 63; 1cc7, A, V, 11; 1cc7, A, V, 22; 1cc7, A, V, 25; 1cc7, A, V, 47; 1cfm, A, A, 5; 1cfm, A, A, 22; 1cfm, A, A, 84; 1cfm, A, C, 21; 1cfm, A, C, 24; 1cfm, A, E, 14; 1cfm, A, F, 45; 1cfm, A, F, 81; 1cfm, A, F, 237; 1cfm, A, G, 17; 1cfm, A, G, 64; 1cfm, A, G, 80; 1cfm, A, G, 116; 1cfm, A, G, 120; 1cfm, A, G, 151; 1cfm, A, G, 177; 1cfm, A, G, 211; 1cfm, A, G, 235; 1cfm, A, G, 236; 1cfm, A, G, 238; 1cfm, A, H, 25; 1cfm, A, L, 77; 1cfm, A, L, 246; 1cfm, A, N, 23; 1cfm, A, N, 111; 1cfm, A, N, 167; 1cfm, A, N, 233; 1cfm, A, P, 2; 1cfm, A, P, 12; 1cfm, A, P, 36; 1cfm, A, P, 78; 1cfm, A, P, 129; 1cfm, A, P, 133; 1cfm, A, P, 135; 1cfm, A, P, 232; 1cfm, A, Q, 6; 1cfm, A, Q, 158; 1cfm, A, Q, 239; 1cfm, A, Q, 247; 1cfm, A, R, 13; 1cfm, A, R, 154; 1cfm, A, S, 166; 1cfm, A, V, 39; 1cfm, A, V, 234; 1cfm, A, V, 245; 1cfm, A, Y, 53; 1cfm, A, Y, 105; 1cs3, A, A, 24; 1cs3, A, A, 47; 1cs3, A, A, 53; 1cs3, A, A, 105; 1cs3, A, A, 106; 1cs3, A, C, 34; 1cs3, A, C, 118; 1cs3, A, D, 35; 1cs3, A, F, 45; 1cs3, A, F, 59; 1cs3, A, H, 48; 1cs3, A, L, 21; 1cs3, A, L, 52; 1cs3, A, L, 84; 1cs3, A, L, 109; 1cs3, A, M, 58; 1cs3, A, N, 25; 1cs3, A, R, 28; 1cs3, A, S, 56; 1cs3, A, T, 89; 1cs3, A, V, 40; 1cs3, A, V, 51; 1cs3, A, Y, 88; 1csp, A, A, 60; 1csp, A, D, 25; 1csp, A, F, 9; 1csp, A, F, 17; 1csp, A, F, 27; 1csp, A, G, 4; 1csp, A, G, 14; 1csp, A, G, 16; 1csp, A, G, 57; 1csp, A, H, 29; 1csp, A, K, 7; 1csp, A, L, 41; 1csp, A, V, 6; 1csp, A, V, 47; 1csp, A, W, 8; 1cyo, A, D, 31; 1cyo, A, D, 53; 1cyo, A, E, 11; 1cyo, A, G, 42; 1cyo, A, G, 51; 1cyo, A, G, 77; 1cyo, A, H, 15; 1cyo, A, T, 33; 1cyo, A, T, 55; 1cyo, A, V, 29; 1cyo, A, W, 22; 1cyo, A, Y, 30; 1d00, A, A, 368; 1d00, A, D, 419; 1d00, A, D, 436; 1d00, A, D, 488; 1d00, A, F, 381; 1d00, A, F, 426; 1d00, A, F, 456; 1d00, A, F, 472; 1d00, A, G, 352; 1d00, A, G, 387; 1d00, A, G, 398; 1d00, A, G, 402; 1d00, A, G, 416; 1d00, A, H, 406; 1d00, A, K, 389; 1d00, A, L, 396; 1d00, A, L, 422; 1d00, A, L, 434; 1d00, A, N, 397; 1d00, A, N, 464; 1d00, A, P, 425; 1d00, A, P, 459; 1d00, A, Q, 437; 1d00, A, S, 378; 1d00, A, S, 408; 1d00, A, T, 383; 1d00, A, T, 431; 1d00, A, V, 496; 1d00, A, W, 356; 1d00, A, W, 424; 1d00, A, Y, 382; 1d00, A, Y, 388; 1d00, A, Y, 484; 1d06, A, A, 165; 1d06, A, D, 148; 1d06, A, D, 249; 1d06, A, E, 176; 1d06, A, F, 170; 1d06, A, F, 226; 1d06, A, G, 157; 1d06, A, G, 205; 1d06, A, N, 181; 1d06, A, P, 187; 1d06, A, S, 153; 1d06, A, T, 204; 1d06, A, Y, 172; 1d5w, A, A, 90; 1d5w, A, A, 93; 1d5w, A, A, 98; 1d5w, A, A, 118; 1d5w, A, D, 10; 1d5w, A, D, 11; 1d5w, A, D, 100; 1d5w, A, F, 101; 1d5w, A, G, 62; 1d5w, A, G, 83; 1d5w, A, G, 85; 1d5w, A, I, 111; 1d5w, A, K, 104; 1d5w, A, L, 23; 1d5w, A, L, 40; 1d5w, A, M, 57; 1d5w, A, P, 77; 1d5w, A, R, 16; 1d5w, A, T, 82; 1d7p, M, A, 2192; 1d7p, M, C, 2174; 1d7p, M, C, 2326; 1d7p, M, D, 2233; 1d7p, M, D, 2288; 1d7p, M, E, 2322; 1d7p, M, F, 2234; 1d7p, M, F, 2260; 1d7p, M, F, 2283; 1d7p, M, G, 2179; 1d7p, M, G, 2247; 1d7p, M, G, 2285; 1d7p, M, G, 2325; 1d7p, M, I, 2185; 1d7p, M, L, 2178; 1d7p, M, L, 2210; 1d7p, M, L, 2230; 1d7p, M, N, 2286; 1d7p, M, P, 2310; 1d7p, M, Q, 2231; 1d7p, M, R, 2209; 1d7p, M, R, 2304; 1d7p, M, R, 2307; 1d7p, M, S, 2193; 1d7p, M, S, 2194; 1d7p, M, S, 2265; 1d7p, M, T, 2245; 1d7p, M, V, 2257; 1d7p, M, W, 2219; 1d7p, M, W, 2271; 1d7p, M, W, 2313; 1dk8, A, A, 157; 1dk8, A, A, 178; 1dk8, A, A, 222; 1dk8, A, C, 158; 1dk8, A, D, 150; 1dk8, A, E, 146; 1dk8, A, F, 139; 1dk8, A, F, 142; 1dk8, A, F, 154; 1dk8, A, F, 219; 1dk8, A, F, 238; 1dk8, A, G, 135; 1dk8, A, I, 181; 1dk8, A, L, 129; 1dk8, A, L, 130; 1dk8, A, L, 143; 1dk8, A, L, 151; 1dk8, A, L, 239; 1dk8, A, M, 230; 1dk8, A, Q, 223; 1dk8, A, S, 237; 1dk8, A, S, 241; 1dk8, A, T, 159; 1dk8, A, T, 229; 1dk8, A, T, 234; 1dk8, A, W, 122; 1dk8, A, W, 155; 1dk8, A, Y, 244; 1dqz, A, A, 130; 1dqz, A, A, 134; 1dqz, A, D, 38; 1dqz, A, D, 79; 1dqz, A, D, 190; 1dqz, A, E, 102; 1dqz, A, E, 228; 1dqz, A, F, 98; 1dqz, A, G, 39; 1dqz, A, G, 71; 1dqz, A, G, 72; 1dqz, A, G, 122; 1dqz, A, G, 127; 1dqz, A, G, 149; 1dqz, A, G, 170; 1dqz, A, G, 171; 1dqz, A, G, 179; 1dqz, A, G, 210; 1dqz, A, G, 247; 1dqz, A, G, 258; 1dqz,

A, H, 260; 1dqz, A, L, 36; 1dqz, A, L, 37; 1dqz, A, L, 99; 1dqz, A, L, 107; 1dqz, A, L, 133; 1dqz, A, L, 198; 1dqz, A, L, 269; 1dqz, A, M, 18; 1dqz, A, M, 103; 1dqz, A, M, 125; 1dqz, A, M, 177; 1dqz, A, P, 69; 1dqz, A, P, 104; 1dqz, A, P, 138; 1dqz, A, P, 191; 1dqz, A, S, 15; 1dqz, A, S, 75; 1dqz, A, S, 124; 1dqz, A, S, 148; 1dqz, A, T, 53; 1dqz, A, T, 97; 1dqz, A, T, 100; 1dqz, A, V, 13; 1dqz, A, V, 66; 1dqz, A, W, 49; 1dqz, A, W, 80; 1dqz, A, W, 95; 1dqz, A, W, 178; 1dqz, A, W, 186; 1dqz, A, W, 262; 1dqz, A, W, 265; 1dqz, A, Y, 35; 1dqz, A, Y, 77; 1dqz, A, Y, 93; 1dqz, A, Y, 264; 1dtj, A, A, 68; 1dtj, A, G, 22; 1dtj, A, G, 25; 1dtj, A, G, 60; 1dtj, A, I, 20; 1dtj, A, I, 39; 1dtj, A, I, 58; 1dtj, A, L, 21; 1dtj, A, L, 28; 1dtj, A, P, 13; 1dtj, A, R, 54; 1e29, A, F, 33; 1e29, A, G, 29; 1e29, A, G, 44; 1e29, A, G, 84; 1e29, A, G, 130; 1e29, A, G, 131; 1e29, A, I, 112; 1e29, A, I, 133; 1e29, A, L, 120; 1e29, A, P, 79; 1e29, A, T, 46; 1e29, A, T, 119; 1e2u, A, A, 160; 1e2u, A, A, 165; 1e2u, A, A, 272; 1e2u, A, A, 358; 1e2u, A, A, 385; 1e2u, A, A, 469; 1e2u, A, A, 497; 1e2u, A, C, 6; 1e2u, A, D, 32; 1e2u, A, D, 143; 1e2u, A, D, 214; 1e2u, A, D, 245; 1e2u, A, D, 407; 1e2u, A, D, 426; 1e2u, A, D, 455; 1e2u, A, D, 461; 1e2u, A, D, 545; 1e2u, A, F, 67; 1e2u, A, F, 299; 1e2u, A, F, 351; 1e2u, A, F, 376; 1e2u, A, F, 400; 1e2u, A, F, 521; 1e2u, A, G, 14; 1e2u, A, G, 22; 1e2u, A, G, 40; 1e2u, A, G, 154; 1e2u, A, G, 157; 1e2u, A, G, 203; 1e2u, A, G, 222; 1e2u, A, G, 243; 1e2u, A, G, 286; 1e2u, A, G, 289; 1e2u, A, G, 304; 1e2u, A, G, 375; 1e2u, A, G, 395; 1e2u, A, G, 405; 1e2u, A, G, 408; 1e2u, A, G, 457; 1e2u, A, G, 508; 1e2u, A, G, 515; 1e2u, A, H, 164; 1e2u, A, H, 266; 1e2u, A, H, 283; 1e2u, A, I, 239; 1e2u, A, I, 355; 1e2u, A, I, 397; 1e2u, A, I, 447; 1e2u, A, I, 512; 1e2u, A, K, 156; 1e2u, A, K, 436; 1e2u, A, L, 34; 1e2u, A, L, 147; 1e2u, A, L, 168; 1e2u, A, L, 213; 1e2u, A, L, 246; 1e2u, A, L, 253; 1e2u, A, L, 270; 1e2u, A, L, 430; 1e2u, A, L, 472; 1e2u, A, L, 484; 1e2u, A, L, 501; 1e2u, A, L, 502; 1e2u, A, L, 504; 1e2u, A, L, 505; 1e2u, A, L, 507; 1e2u, A, L, 530; 1e2u, A, M, 210; 1e2u, A, M, 269; 1e2u, A, N, 72; 1e2u, A, N, 460; 1e2u, A, N, 482; 1e2u, A, P, 224; 1e2u, A, P, 271; 1e2u, A, P, 276; 1e2u, A, P, 424; 1e2u, A, P, 451; 1e2u, A, P, 485; 1e2u, A, P, 516; 1e2u, A, P, 519; 1e2u, A, Q, 31; 1e2u, A, Q, 255; 1e2u, A, Q, 295; 1e2u, A, Q, 458; 1e2u, A, Q, 495; 1e2u, A, R, 325; 1e2u, A, R, 413; 1e2u, A, R, 452; 1e2u, A, S, 464; 1e2u, A, T, 69; 1e2u, A, T, 256; 1e2u, A, T, 265; 1e2u, A, T, 309; 1e2u, A, T, 328; 1e2u, A, T, 431; 1e2u, A, V, 229; 1e2u, A, V, 392; 1e2u, A, W, 75; 1e2u, A, W, 492; 1e2u, A, Y, 161; 1e2u, A, Y, 264; 1e2u, A, Y, 275; 1e2u, A, Y, 322; 1e2u, A, Y, 415; 1e2u, A, Y, 463; 1e7z, A, A, 171; 1e7z, A, A, 230; 1e7z, A, D, 180; 1e7z, A, E, 237; 1e7z, A, G, 160; 1e7z, A, G, 192; 1e7z, A, G, 248; 1e7z, A, H, 168; 1e7z, A, I, 159; 1e7z, A, L, 174; 1e7z, A, L, 193; 1e7z, A, L, 213; 1e7z, A, M, 226; 1e7z, A, M, 228; 1e7z, A, N, 212; 1e7z, A, P, 215; 1e7z, A, P, 241; 1e7z, A, P, 242; 1e7z, A, R, 158; 1e7z, A, R, 187; 1e7z, A, S, 191; 1e7z, A, S, 223; 1e7z, A, S, 231; 1e7z, A, V, 207; 1e7z, A, Y, 175; 1e87, A, A, 118; 1e87, A, C, 85; 1e87, A, C, 96; 1e87, A, C, 113; 1e87, A, C, 173; 1e87, A, C, 186; 1e87, A, C, 194; 1e87, A, D, 128; 1e87, A, F, 131; 1e87, A, G, 144; 1e87, A, I, 193; 1e87, A, K, 196; 1e87, A, L, 120; 1e87, A, L, 132; 1e87, A, L, 145; 1e87, A, S, 100; 1e87, A, W, 89; 1e87, A, W, 106; 1e87, A, W, 142; 1e87, A, W, 153; 1e87, A, W, 155; 1e87, A, W, 192; 1e87, A, Y, 97; 1ed1, A, A, 37; 1ed1, A, A, 100; 1ed1, A, C, 57; 1ed1, A, E, 17; 1ed1, A, E, 40; 1ed1, A, G, 25; 1ed1, A, G, 56; 1ed1, A, G, 71; 1ed1, A, H, 33; 1ed1, A, H, 89; 1ed1, A, L, 8; 1ed1, A, L, 21; 1ed1, A, L, 50; 1ed1, A, L, 51; 1ed1, A, L, 78; 1ed1, A, T, 97; 1ed1, A, W, 36; 1ed1, A, Y, 29; 1ee4, A, A, 151; 1ee4, A, A, 152; 1ee4, A, A, 174; 1ee4, A, A, 193; 1ee4, A, A, 196; 1ee4, A, A, 280; 1ee4, A, A, 370; 1ee4, A, A, 403; 1ee4, A, A, 406; 1ee4, A, C, 243; 1ee4, A, D, 209; 1ee4, A, D, 270; 1ee4, A, D, 286; 1ee4, A, D, 441; 1ee4, A, E, 150; 1ee4, A, E, 191; 1ee4, A, E, 360; 1ee4, A, G, 324; 1ee4, A, G, 371; 1ee4, A, G, 414; 1ee4, A, G, 482; 1ee4, A, I, 123; 1ee4, A, I, 127; 1ee4, A, I, 158; 1ee4, A, I, 292; 1ee4, A, I, 326; 1ee4, A, I, 368; 1ee4, A, K, 359; 1ee4, A, K, 401; 1ee4, A, L, 115; 1ee4, A, L, 155; 1ee4, A, L, 181; 1ee4, A, L, 182; 1ee4, A, L, 197; 1ee4, A, L, 262; 1ee4, A, L, 265; 1ee4, A, L, 304; 1ee4, A, L, 307; 1ee4, A, L, 308; 1ee4, A, L, 320; 1ee4, A, L, 343; 1ee4, A, L, 349; 1ee4, A, L, 350; 1ee4, A, L, 437; 1ee4, A, L, 449; 1ee4, A, M, 139; 1ee4, A, N, 325; 1ee4, A, N, 409; 1ee4, A, P, 121; 1ee4, A, P, 122; 1ee4, A, P, 176; 1ee4, A, P, 250;

1ee4, A, Q, 148; 1ee4, A, Q, 333; 1ee4, A, Q, 375; 1ee4, A, Q, 490; 1ee4, A, R, 112; 1ee4, A, R, 208; 1ee4, A, R, 233; 1ee4, A, R, 321; 1ee4, A, S, 282; 1ee4, A, T, 156; 1ee4, A, T, 184; 1ee4, A, V, 295; 1ee4, A, Y, 283; 1ej4, A, D, 90; 1ej4, A, D, 104; 1ej4, A, D, 127; 1ej4, A, E, 70; 1ej4, A, E, 140; 1ej4, A, F, 66; 1ej4, A, F, 72; 1ej4, A, F, 94; 1ej4, A, F, 215; 1ej4, A, G, 110; 1ej4, A, G, 111; 1ej4, A, G, 151; 1ej4, A, H, 200; 1ej4, A, I, 98; 1ej4, A, I, 179; 1ej4, A, K, 95; 1ej4, A, L, 39; 1ej4, A, L, 85; 1ej4, A, L, 187; 1ej4, A, N, 107; 1ej4, A, N, 213; 1ej4, A, P, 100; 1ej4, A, R, 214; 1ej4, A, V, 69; 1ej4, A, V, 153; 1ej4, A, V, 217; 1ej4, A, W, 43; 1ej4, A, W, 46; 1ej4, A, W, 73; 1ej4, A, W, 113; 1ej4, A, W, 130; 1ej4, A, W, 166; 1ekg, A, D, 167; 1ekg, A, E, 189; 1ekg, A, G, 130; 1ekg, A, G, 141; 1ekg, A, G, 162; 1ekg, A, I, 154; 1ekg, A, K, 147; 1ekg, A, L, 156; 1ekg, A, L, 182; 1ekg, A, L, 186; 1ekg, A, N, 146; 1ekg, A, N, 151; 1ekg, A, P, 159; 1ekg, A, Q, 148; 1ekg, A, Q, 153; 1ekg, A, S, 158; 1ekg, A, S, 161; 1ekg, A, S, 206; 1ekg, A, T, 142; 1ekg, A, V, 131; 1ekg, A, V, 144; 1ekg, A, W, 155; 1ekg, A, W, 173; 1ekg, A, Y, 143; 1eks, A, A, 28; 1eks, A, A, 30; 1eks, A, A, 58; 1eks, A, A, 61; 1eks, A, A, 115; 1eks, A, E, 112; 1eks, A, E, 114; 1eks, A, G, 110; 1eks, A, I, 73; 1eks, A, K, 147; 1eks, A, K, 156; 1eks, A, M, 15; 1eks, A, M, 113; 1eks, A, M, 129; 1eks, A, R, 26; 1eks, A, S, 152; 1eks, A, T, 69; 1eks, A, T, 125; 1eks, A, V, 111; 1eyh, A, A, 27; 1eyh, A, D, 118; 1eyh, A, D, 136; 1eyh, A, E, 143; 1eyh, A, F, 109; 1eyh, A, G, 88; 1eyh, A, G, 120; 1eyh, A, I, 43; 1eyh, A, K, 76; 1eyh, A, L, 84; 1eyh, A, L, 106; 1eyh, A, L, 133; 1eyh, A, M, 57; 1eyh, A, M, 78; 1eyh, A, R, 63; 1eyh, A, R, 72; 1eyh, A, R, 124; 1eyh, A, R, 144; 1eyh, A, T, 28; 1eyh, A, V, 24; 1eyh, A, W, 71; 1eyh, A, Y, 83; 1f0m, A, D, 48; 1f0m, A, F, 30; 1f0m, A, G, 53; 1f0m, A, H, 59; 1f0m, A, I, 21; 1f0m, A, I, 63; 1f0m, A, L, 18; 1f0m, A, M, 23; 1f0m, A, Q, 60; 1f0m, A, V, 14; 1f0m, A, W, 17; 1f0m, A, Y, 26; 1f2l, A, A, 38; 1f2l, A, A, 51; 1f2l, A, C, 8; 1f2l, A, C, 34; 1f2l, A, C, 50; 1f2l, A, D, 52; 1f2l, A, I, 19; 1f2l, A, L, 41; 1f2l, A, L, 65; 1f2l, A, P, 53; 1f2l, A, T, 43; 1f2l, A, V, 58; 1f2l, A, W, 57; 1f2l, A, Y, 27; 1f47, B, A, 62; 1f47, B, A, 105; 1f47, B, A, 109; 1f47, B, D, 118; 1f47, B, F, 59; 1f47, B, F, 70; 1f47, B, F, 85; 1f47, B, F, 98; 1f47, B, G, 25; 1f47, B, G, 36; 1f47, B, G, 40; 1f47, B, G, 68; 1f47, B, G, 81; 1f47, B, G, 114; 1f47, B, H, 46; 1f47, B, H, 48; 1f47, B, I, 44; 1f47, B, L, 28; 1f47, B, M, 101; 1f47, B, N, 63; 1f47, B, P, 89; 1f47, B, R, 47; 1f47, B, S, 60; 1f47, B, T, 79; 1f47, B, V, 15; 1f47, B, Y, 45; 1f56, A, C, 47; 1f56, A, D, 23; 1f56, A, F, 19; 1f56, A, F, 27; 1f56, A, G, 6; 1f56, A, G, 59; 1f56, A, G, 68; 1f56, A, G, 83; 1f56, A, K, 85; 1f56, A, L, 25; 1f56, A, L, 65; 1f56, A, V, 36; 1f56, A, V, 39; 1f56, A, Y, 29; 1f56, A, Y, 44; 1f56, A, Y, 71; 1f5m, A, A, 104; 1f5m, A, C, 91; 1f5m, A, C, 101; 1f5m, A, C, 125; 1f5m, A, C, 152; 1f5m, A, D, 115; 1f5m, A, D, 149; 1f5m, A, D, 151; 1f5m, A, D, 162; 1f5m, A, E, 132; 1f5m, A, F, 69; 1f5m, A, F, 158; 1f5m, A, G, 68; 1f5m, A, G, 83; 1f5m, A, G, 87; 1f5m, A, G, 97; 1f5m, A, G, 99; 1f5m, A, G, 102; 1f5m, A, H, 122; 1f5m, A, I, 94; 1f5m, A, I, 123; 1f5m, A, L, 35; 1f5m, A, L, 80; 1f5m, A, L, 166; 1f5m, A, N, 65; 1f5m, A, P, 84; 1f5m, A, P, 136; 1f5m, A, Q, 86; 1f5m, A, S, 131; 1f5m, A, V, 100; 1f5m, A, V, 113; 1f5m, A, V, 116; 1f5m, A, V, 134; 1f5m, A, V, 147; 1f5m, A, W, 66; 1f5m, A, Y, 70; 1f5m, A, Y, 119; 1fao, A, A, 241; 1fao, A, E, 246; 1fao, A, F, 186; 1fao, A, F, 228; 1fao, A, G, 169; 1fao, A, L, 171; 1fao, A, L, 188; 1fao, A, L, 193; 1fao, A, L, 208; 1fao, A, L, 210; 1fao, A, P, 204; 1fao, A, W, 181; 1fao, A, W, 185; 1fao, A, W, 250; 1fbq, A, F, 197; 1fbq, A, F, 231; 1fbq, A, F, 240; 1fbq, A, F, 248; 1fbq, A, F, 278; 1fbq, A, I, 223; 1fbq, A, K, 259; 1fbq, A, L, 236; 1fbq, A, L, 252; 1fbq, A, N, 253; 1fbq, A, Q, 251; 1fbq, A, W, 216; 1fbq, A, W, 257; 1fbq, A, Y, 255; 1fcy, A, A, 245; 1fcy, A, A, 333; 1fcy, A, A, 360; 1fcy, A, D, 258; 1fcy, A, D, 324; 1fcy, A, D, 325; 1fcy, A, E, 327; 1fcy, A, F, 244; 1fcy, A, F, 251; 1fcy, A, F, 312; 1fcy, A, G, 305; 1fcy, A, K, 264; 1fcy, A, K, 382; 1fcy, A, L, 254; 1fcy, A, L, 262; 1fcy, A, L, 273; 1fcy, A, L, 318; 1fcy, A, L, 330; 1fcy, A, L, 336; 1fcy, A, L, 380; 1fcy, A, L, 386; 1fcy, A, P, 249; 1fcy, A, P, 409; 1fcy, A, Q, 259; 1fcy, A, Q, 354; 1fcy, A, R, 274; 1fcy, A, R, 296; 1fcy, A, R, 341; 1fcy, A, R, 387; 1fcy, A, T, 235; 1fcy, A, T, 285; 1fcy, A, T, 287; 1fcy, A, V, 242; 1fcy, A, Y, 364; 1fh2, A, A, 25; 1fh2, A, C, 10; 1fh2, A, D, 18; 1fh2, A, F, 95; 1fh2, A, G, 53; 1fh2, A, G, 67; 1fh2, A, I, 73; 1fh2, A, L, 58; 1fh2, A, L, 110; 1fh2, A, L, 111; 1fh2, A, M, 119; 1fh2, A, P, 11; 1fh2, A, P,

24; 1fh2, A, P, 113; 1fh2, A, S, 112; 1fh2, A, T, 49; 1fh2, A, T, 118; 1fh2, A, V, 122; 1fh2, A, W, 41; 1fh2, A, Y, 69; 1fh2, A, Y, 78; 1fh2, A, Y, 105; 1fh2, A, Y, 116; 1fhg, A, A, 111; 1fhg, A, A, 117; 1fhg, A, C, 63; 1fhg, A, C, 115; 1fhg, A, D, 109; 1fhg, A, F, 44; 1fhg, A, G, 67; 1fhg, A, G, 122; 1fhg, A, I, 102; 1fhg, A, L, 100; 1fhg, A, L, 130; 1fhg, A, N, 119; 1fhg, A, P, 42; 1fhg, A, P, 69; 1fhg, A, P, 71; 1fhg, A, V, 132; 1fhg, A, W, 75; 1fhg, A, Y, 113; 1fr9, A, A, 13; 1fr9, A, A, 54; 1fr9, A, D, 24; 1fr9, A, D, 71; 1fr9, A, D, 101; 1fr9, A, F, 178; 1fr9, A, G, 9; 1fr9, A, G, 14; 1fr9, A, G, 15; 1fr9, A, G, 78; 1fr9, A, G, 82; 1fr9, A, I, 7; 1fr9, A, K, 25; 1fr9, A, L, 3; 1fr9, A, L, 12; 1fr9, A, L, 27; 1fr9, A, L, 80; 1fr9, A, L, 109; 1fr9, A, L, 113; 1fr9, A, L, 137; 1fr9, A, L, 146; 1fr9, A, L, 150; 1fr9, A, N, 2; 1fr9, A, N, 55; 1fr9, A, N, 180; 1fr9, A, P, 79; 1fr9, A, P, 103; 1fr9, A, P, 106; 1fr9, A, P, 133; 1fr9, A, Q, 46; 1fr9, A, T, 8; 1fr9, A, V, 10; 1fr9, A, V, 11; 1fr9, A, V, 68; 1fso, A, A, 165; 1fso, A, D, 183; 1fso, A, E, 106; 1fso, A, F, 116; 1fso, A, F, 181; 1fso, A, G, 125; 1fso, A, G, 147; 1fso, A, G, 168; 1fso, A, G, 173; 1fso, A, I, 198; 1fso, A, K, 105; 1fso, A, L, 190; 1fso, A, M, 126; 1fso, A, P, 151; 1fso, A, P, 166; 1fso, A, R, 172; 1fso, A, S, 179; 1fso, A, V, 70; 1fso, A, V, 118; 1fso, A, W, 194; 1fso, A, W, 202; 1fso, A, Y, 128; 1fso, A, Y, 149; 1fso, A, Y, 175; 1fxd, A, A, 54; 1fxd, A, C, 18; 1fxd, A, C, 42; 1fxd, A, G, 28; 1g33, A, E, 81; 1g33, A, F, 47; 1g33, A, F, 102; 1g33, A, G, 56; 1g33, A, I, 97; 1g33, A, L, 63; 1g33, A, L, 67; 1g33, A, L, 77; 1g4r, A, A, 288; 1g4r, A, A, 301; 1g4r, A, A, 344; 1g4r, A, D, 26; 1g4r, A, D, 29; 1g4r, A, D, 38; 1g4r, A, D, 44; 1g4r, A, D, 135; 1g4r, A, D, 297; 1g4r, A, D, 342; 1g4r, A, E, 346; 1g4r, A, F, 75; 1g4r, A, F, 115; 1g4r, A, F, 190; 1g4r, A, F, 349; 1g4r, A, F, 388; 1g4r, A, F, 391; 1g4r, A, G, 39; 1g4r, A, G, 64; 1g4r, A, G, 72; 1g4r, A, G, 109; 1g4r, A, G, 137; 1g4r, A, G, 291; 1g4r, A, G, 316; 1g4r, A, H, 210; 1g4r, A, H, 353; 1g4r, A, I, 168; 1g4r, A, I, 386; 1g4r, A, K, 10; 1g4r, A, K, 11; 1g4r, A, K, 170; 1g4r, A, K, 226; 1g4r, A, L, 104; 1g4r, A, L, 129; 1g4r, A, L, 166; 1g4r, A, L, 289; 1g4r, A, L, 300; 1g4r, A, L, 327; 1g4r, A, L, 338; 1g4r, A, L, 347; 1g4r, A, L, 351; 1g4r, A, M, 352; 1g4r, A, N, 222; 1g4r, A, P, 114; 1g4r, A, P, 124; 1g4r, A, P, 182; 1g4r, A, P, 276; 1g4r, A, P, 356; 1g4r, A, Q, 85; 1g4r, A, Q, 130; 1g4r, A, Q, 237; 1g4r, A, R, 25; 1g4r, A, R, 62; 1g4r, A, R, 393; 1g4r, A, S, 126; 1g4r, A, S, 302; 1g4r, A, S, 341; 1g4r, A, T, 350; 1g4r, A, V, 127; 1g4r, A, V, 142; 1g4r, A, V, 343; 1g4r, A, V, 345; 1g4r, A, Y, 21; 1g4r, A, Y, 63; 1g4r, A, Y, 321; 1g62, A, A, 103; 1g62, A, A, 115; 1g62, A, D, 112; 1g62, A, E, 80; 1g62, A, E, 214; 1g62, A, G, 14; 1g62, A, G, 60; 1g62, A, G, 65; 1g62, A, G, 69; 1g62, A, G, 105; 1g62, A, G, 149; 1g62, A, G, 158; 1g62, A, G, 184; 1g62, A, G, 196; 1g62, A, G, 207; 1g62, A, H, 162; 1g62, A, L, 42; 1g62, A, L, 88; 1g62, A, L, 133; 1g62, A, L, 173; 1g62, A, N, 66; 1g62, A, N, 106; 1g62, A, N, 111; 1g62, A, N, 156; 1g62, A, N, 187; 1g62, A, N, 200; 1g62, A, P, 163; 1g62, A, T, 185; 1g62, A, T, 210; 1g62, A, T, 211; 1g62, A, V, 15; 1g62, A, V, 135; 1g62, A, V, 137; 1g62, A, V, 148; 1g62, A, V, 161; 1g6h, A, A, 22; 1g6h, A, A, 151; 1g6h, A, A, 167; 1g6h, A, D, 178; 1g6h, A, E, 163; 1g6h, A, E, 210; 1g6h, A, F, 88; 1g6h, A, G, 33; 1g6h, A, G, 40; 1g6h, A, G, 55; 1g6h, A, G, 62; 1g6h, A, G, 83; 1g6h, A, G, 157; 1g6h, A, G, 229; 1g6h, A, G, 235; 1g6h, A, H, 211; 1g6h, A, I, 39; 1g6h, A, I, 253; 1g6h, A, L, 8; 1g6h, A, L, 94; 1g6h, A, L, 154; 1g6h, A, L, 168; 1g6h, A, M, 177; 1g6h, A, N, 102; 1g6h, A, P, 172; 1g6h, A, P, 180; 1g6h, A, R, 86; 1g6h, A, R, 166; 1g6h, A, V, 99; 1g6h, A, V, 162; 1g6h, A, V, 176; 1g6h, A, V, 225; 1g6h, A, V, 250; 1g6h, A, Y, 254; 1g6n, A, E, 58; 1g6n, A, E, 171; 1g6n, A, F, 76; 1g6n, A, G, 33; 1g6n, A, G, 45; 1g6n, A, G, 67; 1g6n, A, G, 141; 1g6n, A, L, 39; 1g6n, A, L, 75; 1g6n, A, L, 134; 1g6n, A, L, 147; 1g6n, A, L, 150; 1g6n, A, L, 187; 1g6n, A, L, 190; 1g6n, A, R, 123; 1g6n, A, R, 142; 1g6n, A, T, 168; 1g6n, A, V, 47; 1g6n, A, V, 139; 1g6n, A, V, 176; 1g6n, A, V, 183; 1g7c, B, D, 1176; 1g7c, B, G, 1150; 1g7c, B, G, 1154; 1g7c, B, K, 1120; 1g7c, B, K, 1128; 1g7c, B, K, 1167; 1g7c, B, L, 1151; 1g7c, B, L, 1168; 1g7c, B, P, 1129; 1g7c, B, Q, 1196; 1g7c, B, S, 1121; 1g7c, B, S, 1197; 1g7c, B, W, 1153; 1g9o, A, A, 48; 1g9o, A, A, 51; 1g9o, A, D, 57; 1g9o, A, F, 26; 1g9o, A, G, 23; 1g9o, A, G, 25; 1g9o, A, G, 36; 1g9o, A, G, 52; 1g9o, A, G, 56; 1g9o, A, I, 79; 1g9o, A, L, 53; 1g9o, A, N, 63; 1g9o, A, R, 13; 1g9o, A, V, 42; 1g9o, A, V, 62; 1g9o, A, V, 67; 1g9o, A, V, 75; 1g9o, A, V, 76; 1h4y, A, G, 19; 1h4y, A, G, 59; 1h4y,

A, G, 61; 1h4y, A, G, 65; 1h4y, A, G, 75; 1h4y, A, L, 21; 1h4y, A, L, 62; 1h4y, A, M, 55; 1h4y, A, M, 67; 1h4y, A, R, 66; 1h4y, A, S, 93; 1h4y, A, V, 26; 1hjp, A, A, 121; 1hjp, A, A, 159; 1hjp, A, A, 163; 1hjp, A, A, 166; 1hjp, A, A, 177; 1hjp, A, A, 185; 1hjp, A, A, 189; 1hjp, A, A, 198; 1hjp, A, A, 201; 1hjp, A, A, 202; 1hjp, A, E, 160; 1hjp, A, E, 176; 1hjp, A, E, 197; 1hjp, A, F, 63; 1hjp, A, F, 72; 1hjp, A, G, 7; 1hjp, A, G, 62; 1hjp, A, G, 82; 1hjp, A, G, 117; 1hjp, A, I, 184; 1hjp, A, I, 195; 1hjp, A, L, 60; 1hjp, A, L, 75; 1hjp, A, L, 167; 1hjp, A, L, 194; 1hjp, A, L, 199; 1hjp, A, M, 180; 1hjp, A, Q, 175; 1hjp, A, S, 191; 1hjp, A, T, 193; 1hjp, A, V, 164; 1hjp, A, V, 168; 1hjp, A, V, 181; 1hjp, A, Y, 172; 1hus, A, A, 38; 1hus, A, A, 45; 1hus, A, A, 64; 1hus, A, A, 126; 1hus, A, A, 144; 1hus, A, D, 14; 1hus, A, E, 73; 1hus, A, E, 122; 1hus, A, G, 131; 1hus, A, H, 141; 1hus, A, I, 41; 1hus, A, K, 136; 1hus, A, L, 98; 1hus, A, L, 119; 1hus, A, N, 147; 1hus, A, P, 70; 1hus, A, P, 87; 1hus, A, R, 94; 1hus, A, R, 101; 1hus, A, R, 110; 1hus, A, V, 74; 1hus, A, V, 86; 1hus, A, W, 102; 1hus, A, Y, 43; 1i07, A, A, 17; 1i07, A, E, 22; 1i07, A, G, 48; 1i07, A, G, 51; 1i07, A, P, 54; 1i07, A, V, 53; 1i07, A, W, 40; 1i07, A, W, 41; 1i6a, A, A, 147; 1i6a, A, A, 244; 1i6a, A, C, 199; 1i6a, A, C, 208; 1i6a, A, D, 142; 1i6a, A, E, 126; 1i6a, A, G, 91; 1i6a, A, G, 96; 1i6a, A, G, 197; 1i6a, A, G, 234; 1i6a, A, G, 236; 1i6a, A, H, 198; 1i6a, A, I, 98; 1i6a, A, L, 105; 1i6a, A, L, 106; 1i6a, A, L, 132; 1i6a, A, L, 136; 1i6a, A, L, 160; 1i6a, A, L, 186; 1i6a, A, L, 192; 1i6a, A, L, 194; 1i6a, A, L, 224; 1i6a, A, L, 270; 1i6a, A, M, 193; 1i6a, A, M, 230; 1i6a, A, P, 99; 1i6a, A, P, 103; 1i6a, A, P, 241; 1i6a, A, P, 264; 1i6a, A, Q, 203; 1i6a, A, R, 201; 1i6a, A, R, 266; 1i6a, A, R, 273; 1i6a, A, T, 100; 1i6a, A, T, 129; 1i6a, A, T, 226; 1i6a, A, T, 238; 1i6a, A, V, 149; 1i6a, A, V, 231; 1i6a, A, Y, 104; 1i81, A, D, 44; 1i81, A, E, 56; 1i81, A, G, 38; 1i81, A, G, 67; 1i81, A, G, 73; 1i81, A, I, 28; 1i81, A, I, 76; 1i81, A, L, 18; 1i81, A, L, 30; 1i81, A, L, 40; 1i81, A, L, 51; 1i81, A, N, 48; 1i81, A, V, 26; 1inn, A, A, 19; 1inn, A, A, 64; 1inn, A, A, 136; 1inn, A, A, 140; 1inn, A, D, 13; 1inn, A, D, 40; 1inn, A, D, 76; 1inn, A, E, 60; 1inn, A, G, 85; 1inn, A, H, 14; 1inn, A, L, 62; 1inn, A, L, 133; 1inn, A, P, 20; 1inn, A, P, 46; 1inn, A, P, 79; 1inn, A, R, 23; 1inn, A, R, 42; 1inn, A, R, 68; 1inn, A, T, 84; 1inn, A, V, 22; 1io3, A, A, 50; 1io3, A, F, 10; 1io3, A, G, 6; 1io3, A, G, 33; 1io3, A, G, 83; 1io3, A, I, 97; 1io3, A, L, 31; 1io3, A, P, 70; 1io3, A, Y, 96; 1ird, B, A, 227; 1ird, B, F, 303; 1ird, B, F, 322; 1ird, B, G, 224; 1ird, B, G, 264; 1ird, B, K, 332; 1ird, B, L, 228; 1ird, B, P, 300; 1ird, B, R, 230; 1ird, B, S, 289; 1ird, B, T, 323; 1ird, B, V, 337; 1ird, B, Y, 345; 1irn, A, F, 49; 1irn, A, G, 18; 1irn, A, K, 46; 1irn, A, M, 1; 1irn, A, P, 40; 1irn, A, T, 28; 1irn, A, W, 37; 1irn, A, Y, 13; 1ixg, A, A, 227; 1ixg, A, D, 51; 1ixg, A, D, 113; 1ixg, A, D, 137; 1ixg, A, E, 195; 1ixg, A, F, 52; 1ixg, A, F, 145; 1ixg, A, G, 6; 1ixg, A, G, 8; 1ixg, A, G, 41; 1ixg, A, G, 77; 1ixg, A, G, 106; 1ixg, A, G, 161; 1ixg, A, G, 163; 1ixg, A, G, 171; 1ixg, A, G, 173; 1ixg, A, G, 176; 1ixg, A, G, 179; 1ixg, A, G, 192; 1ixg, A, G, 284; 1ixg, A, G, 317; 1ixg, A, I, 103; 1ixg, A, I, 108; 1ixg, A, I, 116; 1ixg, A, I, 130; 1ixg, A, I, 180; 1ixg, A, I, 191; 1ixg, A, I, 252; 1ixg, A, L, 93; 1ixg, A, L, 95; 1ixg, A, L, 125; 1ixg, A, L, 149; 1ixg, A, L, 296; 1ixg, A, N, 84; 1ixg, A, N, 120; 1ixg, A, N, 177; 1ixg, A, P, 73; 1ixg, A, P, 126; 1ixg, A, P, 169; 1ixg, A, P, 219; 1ixg, A, P, 251; 1ixg, A, S, 39; 1ixg, A, S, 55; 1ixg, A, S, 142; 1ixg, A, S, 211; 1ixg, A, T, 146; 1ixg, A, T, 256; 1ixg, A, V, 50; 1ixg, A, V, 133; 1ixg, A, V, 194; 1ixg, A, W, 19; 1ixg, A, W, 111; 1ixg, A, W, 156; 1ixg, A, W, 168; 1ixg, A, Y, 33; 1ixg, A, Y, 193; 1ixg, A, Y, 198; 1ixg, A, Y, 293; 1j7a, A, A, 30; 1j7a, A, E, 94; 1j7a, A, G, 56; 1j7a, A, G, 74; 1j7a, A, L, 27; 1j7a, A, L, 37; 1j7a, A, L, 66; 1j7a, A, P, 38; 1j7a, A, P, 83; 1j8q, A, A, 2; 1j8q, A, A, 19; 1j8q, A, A, 89; 1j8q, A, A, 104; 1j8q, A, D, 70; 1j8q, A, E, 16; 1j8q, A, F, 50; 1j8q, A, F, 71; 1j8q, A, F, 91; 1j8q, A, G, 9; 1j8q, A, G, 56; 1j8q, A, G, 92; 1j8q, A, G, 116; 1j8q, A, I, 22; 1j8q, A, Q, 68; 1j8q, A, R, 86; 1j8q, A, V, 88; 1jaf, A, A, 111; 1jaf, A, F, 2; 1jaf, A, G, 100; 1jaf, A, G, 103; 1jaf, A, K, 120; 1jaf, A, L, 96; 1jaf, A, M, 29; 1jaf, A, W, 76; 1jd1, A, A, 13; 1jd1, A, A, 125; 1jd1, A, C, 10; 1jd1, A, D, 83; 1jd1, A, E, 120; 1jd1, A, F, 87; 1jd1, A, F, 98; 1jd1, A, G, 34; 1jd1, A, H, 22; 1jd1, A, K, 76; 1jd1, A, L, 113; 1jd1, A, N, 59; 1jd1, A, N, 91; 1jd1, A, P, 14; 1jd1, A, P, 103; 1jd1, A, Q, 35; 1jd1, A, R, 105; 1jd1, A, S, 33; 1jd1, A, V, 110; 1jd1, A, Y, 20; 1jd1, A, Y, 94; 1jet, A, A, 1; 1jet, A, A, 61; 1jet, A, A, 80; 1jet, A, A, 90; 1jet, A, A, 138;

1jet, A, A, 292; 1jet, A, A, 314; 1jet, A, A, 344; 1jet, A, A, 351; 1jet, A, A, 375; 1jet, A, A, 469; 1jet, A, D, 27; 1jet, A, D, 92; 1jet, A, D, 242; 1jet, A, D, 298; 1jet, A, D, 447; 1jet, A, E, 22; 1jet, A, E, 45; 1jet, A, E, 350; 1jet, A, E, 470; 1jet, A, F, 74; 1jet, A, F, 93; 1jet, A, F, 144; 1jet, A, F, 155; 1jet, A, F, 426; 1jet, A, G, 46; 1jet, A, G, 59; 1jet, A, G, 85; 1jet, A, G, 135; 1jet, A, G, 176; 1jet, A, G, 189; 1jet, A, G, 387; 1jet, A, G, 497; 1jet, A, L, 26; 1jet, A, L, 47; 1jet, A, L, 76; 1jet, A, L, 134; 1jet, A, L, 148; 1jet, A, L, 193; 1jet, A, L, 347; 1jet, A, L, 348; 1jet, A, L, 386; 1jet, A, L, 392; 1jet, A, L, 427; 1jet, A, L, 449; 1jet, A, L, 490; 1jet, A, N, 120; 1jet, A, N, 188; 1jet, A, N, 207; 1jet, A, N, 278; 1jet, A, N, 366; 1jet, A, N, 437; 1jet, A, P, 28; 1jet, A, P, 103; 1jet, A, P, 153; 1jet, A, P, 166; 1jet, A, P, 249; 1jet, A, P, 313; 1jet, A, P, 319; 1jet, A, P, 481; 1jet, A, R, 77; 1jet, A, R, 99; 1jet, A, R, 290; 1jet, A, R, 299; 1jet, A, R, 462; 1jet, A, S, 83; 1jet, A, T, 73; 1jet, A, T, 89; 1jet, A, T, 105; 1jet, A, T, 143; 1jet, A, V, 88; 1jet, A, V, 136; 1jet, A, V, 186; 1jet, A, V, 289; 1jet, A, W, 64; 1jet, A, W, 82; 1jet, A, W, 211; 1jet, A, W, 382; 1jet, A, Y, 154; 1jet, A, Y, 210; 1jet, A, Y, 365; 1jet, A, Y, 420; 1jet, A, Y, 513; 1ji6, A, A, 130; 1ji6, A, A, 201; 1ji6, A, A, 202; 1ji6, A, A, 573; 1ji6, A, C, 244; 1ji6, A, D, 211; 1ji6, A, D, 279; 1ji6, A, D, 288; 1ji6, A, D, 303; 1ji6, A, D, 643; 1ji6, A, E, 115; 1ji6, A, E, 326; 1ji6, A, E, 646; 1ji6, A, F, 110; 1ji6, A, F, 185; 1ji6, A, F, 194; 1ji6, A, F, 266; 1ji6, A, F, 269; 1ji6, A, F, 284; 1ji6, A, F, 305; 1ji6, A, F, 426; 1ji6, A, F, 535; 1ji6, A, F, 609; 1ji6, A, F, 647; 1ji6, A, G, 136; 1ji6, A, G, 216; 1ji6, A, G, 220; 1ji6, A, G, 251; 1ji6, A, G, 362; 1ji6, A, G, 420; 1ji6, A, G, 532; 1ji6, A, G, 534; 1ji6, A, G, 537; 1ji6, A, G, 538; 1ji6, A, H, 205; 1ji6, A, H, 333; 1ji6, A, H, 474; 1ji6, A, H, 498; 1ji6, A, I, 118; 1ji6, A, I, 122; 1ji6, A, I, 507; 1ji6, A, I, 512; 1ji6, A, I, 529; 1ji6, A, I, 645; 1ji6, A, K, 120; 1ji6, A, K, 127; 1ji6, A, K, 210; 1ji6, A, K, 519; 1ji6, A, L, 77; 1ji6, A, L, 117; 1ji6, A, L, 134; 1ji6, A, L, 137; 1ji6, A, L, 195; 1ji6, A, L, 206; 1ji6, A, L, 209; 1ji6, A, L, 252; 1ji6, A, L, 278; 1ji6, A, L, 300; 1ji6, A, L, 334; 1ji6, A, L, 476; 1ji6, A, L, 515; 1ji6, A, L, 604; 1ji6, A, N, 203; 1ji6, A, N, 267; 1ji6, A, N, 505; 1ji6, A, N, 539; 1ji6, A, P, 101; 1ji6, A, P, 285; 1ji6, A, P, 308; 1ji6, A, P, 332; 1ji6, A, P, 460; 1ji6, A, P, 492; 1ji6, A, P, 516; 1ji6, A, P, 533; 1ji6, A, P, 649; 1ji6, A, Q, 164; 1ji6, A, Q, 200; 1ji6, A, Q, 514; 1ji6, A, Q, 564; 1ji6, A, R, 270; 1ji6, A, R, 271; 1ji6, A, R, 302; 1ji6, A, R, 330; 1ji6, A, R, 567; 1ji6, A, R, 569; 1ji6, A, R, 571; 1ji6, A, S, 293; 1ji6, A, S, 455; 1ji6, A, S, 473; 1ji6, A, S, 500; 1ji6, A, T, 197; 1ji6, A, T, 204; 1ji6, A, T, 274; 1ji6, A, T, 301; 1ji6, A, T, 306; 1ji6, A, T, 497; 1ji6, A, T, 513; 1ji6, A, T, 536; 1ji6, A, T, 597; 1ji6, A, V, 245; 1ji6, A, V, 277; 1ji6, A, V, 282; 1ji6, A, V, 518; 1ji6, A, W, 107; 1ji6, A, W, 151; 1ji6, A, W, 219; 1ji6, A, W, 263; 1ji6, A, W, 360; 1ji6, A, W, 496; 1ji6, A, Y, 144; 1ji6, A, Y, 198; 1ji6, A, Y, 230; 1ji6, A, Y, 240; 1ji6, A, Y, 248; 1ji6, A, Y, 287; 1ji6, A, Y, 292; 1ji6, A, Y, 566; 1ji6, A, Y, 572; 1jot, A, D, 6; 1jot, A, E, 63; 1jot, A, F, 86; 1jot, A, F, 104; 1jot, A, F, 116; 1jot, A, G, 70; 1jot, A, G, 94; 1jot, A, G, 97; 1jot, A, G, 115; 1jot, A, G, 118; 1jot, A, L, 84; 1jot, A, N, 89; 1jot, A, R, 13; 1jot, A, S, 83; 1jot, A, S, 119; 1jot, A, T, 88; 1jot, A, V, 114; 1jot, A, Y, 32; 1jqf, A, A, 57; 1jqf, A, A, 70; 1jqf, A, A, 97; 1jqf, A, A, 99; 1jqf, A, A, 149; 1jqf, A, A, 191; 1jqf, A, A, 203; 1jqf, A, C, 9; 1jqf, A, C, 19; 1jqf, A, C, 39; 1jqf, A, C, 48; 1jqf, A, C, 118; 1jqf, A, C, 158; 1jqf, A, C, 171; 1jqf, A, C, 174; 1jqf, A, C, 194; 1jqf, A, C, 227; 1jqf, A, C, 241; 1jqf, A, C, 331; 1jqf, A, D, 58; 1jqf, A, D, 297; 1jqf, A, E, 15; 1jqf, A, E, 83; 1jqf, A, F, 204; 1jqf, A, F, 285; 1jqf, A, F, 295; 1jqf, A, G, 114; 1jqf, A, G, 121; 1jqf, A, G, 190; 1jqf, A, G, 200; 1jqf, A, H, 119; 1jqf, A, I, 52; 1jqf, A, K, 18; 1jqf, A, K, 102; 1jqf, A, L, 77; 1jqf, A, L, 112; 1jqf, A, L, 135; 1jqf, A, L, 170; 1jqf, A, L, 195; 1jqf, A, L, 225; 1jqf, A, L, 226; 1jqf, A, L, 243; 1jqf, A, L, 267; 1jqf, A, L, 293; 1jqf, A, L, 315; 1jqf, A, P, 79; 1jqf, A, P, 131; 1jqf, A, P, 160; 1jqf, A, P, 247; 1jqf, A, R, 254; 1jqf, A, S, 12; 1jqf, A, S, 117; 1jqf, A, S, 157; 1jqf, A, V, 98; 1jqf, A, V, 100; 1jqf, A, V, 202; 1jqf, A, V, 251; 1jqf, A, W, 8; 1jqf, A, W, 128; 1jqf, A, Y, 85; 1jqf, A, Y, 96; 1jqf, A, Y, 185; 1jqf, A, Y, 319; 1kiv, A, C, 1; 1kiv, A, C, 22; 1kiv, A, C, 51; 1kiv, A, C, 63; 1kiv, A, C, 75; 1kiv, A, C, 80; 1kiv, A, G, 6; 1kiv, A, G, 11; 1kiv, A, N, 49; 1kiv, A, N, 53; 1kiv, A, P, 54; 1kiv, A, P, 61; 1kiv, A, R, 10; 1kiv, A, R, 52; 1kiv, A, T, 16; 1kiv, A, W, 25; 1kiv, A, W, 62; 1kiv, A, Y, 9; 1klo, A, C, 11; 1klo, A, C, 13; 1klo, A, C, 33; 1klo, A, C, 42; 1klo, A, C,

45; 1klo, A, C, 63; 1klo, A, C, 66; 1klo, A, C, 68; 1klo, A, C, 80; 1klo, A, C, 87; 1klo, A, C, 90; 1klo, A, C, 99; 1klo, A, C, 102; 1klo, A, C, 119; 1klo, A, C, 122; 1klo, A, C, 124; 1klo, A, C, 136; 1klo, A, C, 143; 1klo, A, C, 145; 1klo, A, C, 154; 1klo, A, C, 157; 1klo, A, C, 170; 1klo, A, G, 39; 1klo, A, G, 85; 1klo, A, G, 96; 1klo, A, G, 105; 1klo, A, G, 128; 1klo, A, G, 141; 1klo, A, G, 151; 1klo, A, G, 169; 1klo, A, P, 53; 1klo, A, T, 94; 1klo, A, V, 149; 1lit, A, A, 38; 1lit, A, C, 14; 1lit, A, C, 25; 1lit, A, C, 42; 1lit, A, C, 115; 1lit, A, C, 132; 1lit, A, C, 140; 1lit, A, D, 39; 1lit, A, E, 58; 1lit, A, F, 61; 1lit, A, G, 48; 1lit, A, G, 79; 1lit, A, I, 78; 1lit, A, L, 50; 1lit, A, L, 80; 1lit, A, N, 49; 1lit, A, S, 52; 1lit, A, S, 93; 1lit, A, T, 18; 1lit, A, W, 35; 1lit, A, W, 77; 1lit, A, W, 91; 1lit, A, W, 102; 1lit, A, W, 127; 1lit, A, Y, 26; 1lit, A, Y, 99; 1lou, A, E, 5; 1lou, A, F, 60; 1lou, A, G, 34; 1lou, A, G, 44; 1lou, A, G, 58; 1lou, A, I, 52; 1lou, A, L, 43; 1lou, A, L, 48; 1lou, A, L, 75; 1lou, A, P, 12; 1lou, A, R, 2; 1lou, A, R, 87; 1lou, A, V, 9; 1lou, A, V, 85; 1lou, A, Y, 4; 1mff, A, A, 27; 1mff, A, F, 49; 1mff, A, F, 113; 1mff, A, G, 65; 1mff, A, I, 64; 1mff, A, I, 96; 1mff, A, K, 32; 1mff, A, L, 87; 1mff, A, N, 8; 1mff, A, P, 1; 1mff, A, R, 93; 1mff, A, S, 63; 1mff, A, S, 76; 1mff, A, T, 7; 1mff, A, T, 112; 1mff, A, Y, 36; 1mff, A, Y, 98; 1mho, A, E, 39; 1mho, A, F, 14; 1mho, A, F, 70; 1mho, A, K, 29; 1mho, A, K, 33; 1mho, A, L, 27; 1mho, A, L, 32; 1mho, A, L, 35; 1mho, A, L, 60; 1mho, A, Y, 17; 1mpc, A, A, 51; 1mpc, A, A, 96; 1mpc, A, A, 105; 1mpc, A, A, 223; 1mpc, A, A, 276; 1mpc, A, A, 324; 1mpc, A, A, 346; 1mpc, A, A, 360; 1mpc, A, D, 58; 1mpc, A, D, 65; 1mpc, A, F, 27; 1mpc, A, F, 156; 1mpc, A, F, 169; 1mpc, A, F, 217; 1mpc, A, F, 258; 1mpc, A, F, 279; 1mpc, A, G, 54; 1mpc, A, G, 56; 1mpc, A, G, 68; 1mpc, A, G, 101; 1mpc, A, G, 165; 1mpc, A, G, 166; 1mpc, A, G, 182; 1mpc, A, G, 187; 1mpc, A, G, 220; 1mpc, A, G, 228; 1mpc, A, G, 243; 1mpc, A, G, 260; 1mpc, A, G, 327; 1mpc, A, H, 64; 1mpc, A, I, 226; 1mpc, A, I, 368; 1mpc, A, K, 119; 1mpc, A, L, 20; 1mpc, A, L, 75; 1mpc, A, L, 115; 1mpc, A, L, 361; 1mpc, A, M, 330; 1mpc, A, M, 336; 1mpc, A, N, 118; 1mpc, A, N, 272; 1mpc, A, N, 332; 1mpc, A, P, 57; 1mpc, A, P, 107; 1mpc, A, P, 126; 1mpc, A, P, 229; 1mpc, A, P, 248; 1mpc, A, P, 257; 1mpc, A, P, 331; 1mpc, A, P, 334; 1mpc, A, R, 230; 1mpc, A, S, 270; 1mpc, A, T, 31; 1mpc, A, V, 35; 1mpc, A, V, 37; 1mpc, A, V, 244; 1mpc, A, W, 10; 1mpc, A, W, 340; 1mpc, A, Y, 117; 1mpc, A, Y, 155; 1mpc, A, Y, 167; 1msa, A, C, 52; 1msa, A, G, 60; 1msa, A, L, 4; 1msa, A, L, 10; 1msa, A, L, 31; 1msa, A, S, 75; 1msa, A, T, 45; 1msa, A, V, 32; 1msa, A, V, 63; 1msa, A, W, 41; 1msa, A, W, 73; 1mzm, A, A, 40; 1mzm, A, C, 14; 1mzm, A, C, 29; 1mzm, A, C, 30; 1mzm, A, C, 50; 1mzm, A, C, 52; 1mzm, A, C, 75; 1mzm, A, D, 45; 1mzm, A, G, 32; 1mzm, A, I, 83; 1mzm, A, L, 36; 1mzm, A, P, 25; 1mzm, A, P, 72; 1mzm, A, V, 7; 1mzm, A, Y, 17; 1nat, A, A, 33; 1nat, A, A, 39; 1nat, A, A, 98; 1nat, A, D, 10; 1nat, A, D, 11; 1nat, A, D, 49; 1nat, A, D, 54; 1nat, A, F, 23; 1nat, A, G, 27; 1nat, A, G, 36; 1nat, A, G, 62; 1nat, A, G, 97; 1nat, A, I, 57; 1nat, A, K, 104; 1nat, A, L, 7; 1nat, A, P, 58; 1nat, A, T, 82; 1nat, A, T, 100; 1nat, A, V, 9; 1ndd, A, D, 52; 1ndd, A, G, 10; 1ndd, A, I, 44; 1ndd, A, K, 27; 1ndd, A, L, 43; 1ndd, A, Q, 40; 1ndd, A, Q, 41; 1ndd, A, R, 42; 1ndd, A, Y, 59; 1nt3, A, A, 88; 1nt3, A, C, 14; 1nt3, A, C, 57; 1nt3, A, C, 67; 1nt3, A, C, 79; 1nt3, A, C, 108; 1nt3, A, C, 110; 1nt3, A, D, 29; 1nt3, A, D, 71; 1nt3, A, E, 54; 1nt3, A, F, 52; 1nt3, A, I, 102; 1nt3, A, I, 104; 1nt3, A, Q, 50; 1nt3, A, R, 103; 1nt3, A, S, 16; 1nt3, A, S, 77; 1nt3, A, T, 55; 1nt3, A, V, 35; 1nt3, A, V, 86; 1nt3, A, W, 75; 1nt3, A, W, 99; 1opc, A, D, 202; 1opc, A, E, 165; 1opc, A, G, 229; 1opc, A, I, 222; 1opc, A, L, 161; 1opc, A, L, 169; 1opc, A, L, 185; 1opc, A, L, 208; 1opc, A, M, 211; 1opc, A, P, 219; 1opc, A, R, 199; 1opc, A, R, 207; 1opc, A, R, 209; 1opc, A, T, 162; 1opc, A, T, 224; 1opc, A, V, 168; 1opc, A, Y, 230; 1pcz, A, F, 43; 1pcz, A, F, 60; 1pcz, A, F, 134; 1pcz, A, F, 151; 1pcz, A, G, 63; 1pcz, A, G, 69; 1pcz, A, G, 136; 1pcz, A, G, 154; 1pcz, A, G, 160; 1pcz, A, L, 26; 1pcz, A, L, 58; 1pcz, A, L, 117; 1pcz, A, L, 149; 1pcz, A, L, 178; 1pcz, A, N, 13; 1pcz, A, N, 104; 1pcz, A, P, 53; 1pcz, A, P, 131; 1pcz, A, P, 144; 1pcz, A, Q, 103; 1pcz, A, S, 153; 1pcz, A, S, 159; 1pcz, A, T, 68; 1pcz, A, V, 15; 1pcz, A, V, 66; 1pcz, A, V, 106; 1pcz, A, V, 137; 1pcz, A, V, 147; 1pcz, A, V, 157; 1pcz, A, Y, 38; 1pcz, A, Y, 129; 1pcz, A, Y, 139; 1phn, B, A, 12; 1phn, B, A, 55; 1phn, B, A, 101; 1phn, B, C, 155; 1phn, B, D, 103; 1phn, B, E, 161; 1phn, B, F, 166; 1phn, B, G, 71;

1phn, B, G, 102; 1phn, B, G, 114; 1phn, B, I, 51; 1phn, B, K, 137; 1phn, B, L, 85; 1phn, B, L, 92; 1phn, B, N, 47; 1phn, B, R, 37; 1phn, B, R, 93; 1phn, B, Y, 76; 1phn, B, Y, 97; 1phn, B, Y, 165; 1plf, A, C, 25; 1plf, A, C, 27; 1plf, A, C, 51; 1plf, A, C, 67; 1plf, A, I, 39; 1plf, A, I, 57; 1plf, A, L, 60; 1plf, A, L, 68; 1plf, A, Q, 55; 1plf, A, Q, 70; 1psz, A, A, 82; 1psz, A, A, 140; 1psz, A, A, 207; 1psz, A, A, 275; 1psz, A, D, 44; 1psz, A, D, 75; 1psz, A, D, 162; 1psz, A, E, 93; 1psz, A, E, 254; 1psz, A, F, 150; 1psz, A, F, 208; 1psz, A, F, 252; 1psz, A, F, 278; 1psz, A, G, 51; 1psz, A, G, 63; 1psz, A, G, 89; 1psz, A, I, 154; 1psz, A, I, 277; 1psz, A, I, 282; 1psz, A, I, 303; 1psz, A, K, 199; 1psz, A, L, 56; 1psz, A, L, 92; 1psz, A, L, 158; 1psz, A, L, 307; 1psz, A, N, 88; 1psz, A, N, 146; 1psz, A, N, 172; 1psz, A, P, 71; 1psz, A, P, 138; 1psz, A, S, 291; 1psz, A, T, 38; 1psz, A, T, 203; 1psz, A, V, 35; 1psz, A, V, 202; 1psz, A, W, 141; 1psz, A, Y, 69; 1psz, A, Y, 169; 1psz, A, Y, 176; 1psz, A, Y, 210; 1psz, A, Y, 215; 1psz, A, Y, 292; 1ptx, A, C, 12; 1ptx, A, C, 16; 1ptx, A, C, 22; 1ptx, A, C, 26; 1ptx, A, C, 36; 1ptx, A, C, 46; 1ptx, A, C, 48; 1ptx, A, C, 63; 1ptx, A, G, 34; 1ptx, A, L, 51; 1ptx, A, P, 52; 1ptx, A, Y, 5; 1ptx, A, Y, 35; 1qdv, A, E, 75; 1qdv, A, E, 112; 1qdv, A, F, 77; 1qdv, A, F, 78; 1qdv, A, F, 85; 1qdv, A, F, 109; 1qdv, A, F, 115; 1qdv, A, G, 96; 1qdv, A, I, 88; 1qdv, A, L, 51; 1qdv, A, L, 59; 1qdv, A, L, 89; 1qdv, A, L, 98; 1qdv, A, N, 38; 1qdv, A, P, 101; 1qdv, A, R, 80; 1qdv, A, T, 50; 1qdv, A, T, 57; 1qdv, A, Y, 92; 1qfg, A, A, 30; 1qfg, A, A, 69; 1qfg, A, A, 216; 1qfg, A, C, 703; 1qfg, A, C, 709; 1qfg, A, D, 88; 1qfg, A, D, 179; 1qfg, A, D, 182; 1qfg, A, D, 358; 1qfg, A, D, 379; 1qfg, A, D, 449; 1qfg, A, D, 465; 1qfg, A, D, 603; 1qfg, A, D, 670; 1qfg, A, D, 696; 1qfg, A, E, 127; 1qfg, A, E, 533; 1qfg, A, E, 572; 1qfg, A, E, 575; 1qfg, A, E, 582; 1qfg, A, E, 584; 1qfg, A, F, 95; 1qfg, A, F, 511; 1qfg, A, F, 723; 1qfg, A, G, 75; 1qfg, A, G, 82; 1qfg, A, G, 94; 1qfg, A, G, 108; 1qfg, A, G, 134; 1qfg, A, G, 141; 1qfg, A, G, 146; 1qfg, A, G, 147; 1qfg, A, G, 169; 1qfg, A, G, 251; 1qfg, A, G, 283; 1qfg, A, G, 377; 1qfg, A, G, 444; 1qfg, A, G, 461; 1qfg, A, G, 492; 1qfg, A, G, 500; 1qfg, A, G, 529; 1qfg, A, G, 535; 1qfg, A, G, 574; 1qfg, A, G, 580; 1qfg, A, G, 640; 1qfg, A, G, 644; 1qfg, A, G, 646; 1qfg, A, G, 651; 1qfg, A, G, 712; 1qfg, A, H, 288; 1qfg, A, H, 372; 1qfg, A, H, 690; 1qfg, A, K, 38; 1qfg, A, K, 154; 1qfg, A, K, 537; 1qfg, A, L, 109; 1qfg, A, L, 139; 1qfg, A, L, 375; 1qfg, A, L, 694; 1qfg, A, N, 107; 1qfg, A, N, 299; 1qfg, A, N, 558; 1qfg, A, N, 659; 1qfg, A, N, 693; 1qfg, A, P, 47; 1qfg, A, P, 123; 1qfg, A, P, 135; 1qfg, A, P, 145; 1qfg, A, P, 156; 1qfg, A, P, 217; 1qfg, A, P, 248; 1qfg, A, P, 405; 1qfg, A, P, 503; 1qfg, A, P, 513; 1qfg, A, P, 526; 1qfg, A, P, 540; 1qfg, A, P, 621; 1qfg, A, Q, 48; 1qfg, A, Q, 442; 1qfg, A, Q, 448; 1qfg, A, Q, 450; 1qfg, A, Q, 531; 1qfg, A, R, 93; 1qfg, A, R, 193; 1qfg, A, R, 199; 1qfg, A, R, 212; 1qfg, A, R, 301; 1qfg, A, R, 343; 1qfg, A, R, 463; 1qfg, A, R, 490; 1qfg, A, R, 648; 1qfg, A, R, 715; 1qfg, A, S, 137; 1qfg, A, S, 506; 1qfg, A, S, 510; 1qfg, A, S, 626; 1qfg, A, T, 37; 1qfg, A, T, 39; 1qfg, A, T, 227; 1qfg, A, T, 230; 1qfg, A, T, 667; 1qfg, A, V, 52; 1qfg, A, V, 76; 1qfg, A, W, 221; 1qfg, A, W, 628; 1qfg, A, Y, 72; 1qfg, A, Y, 140; 1qfg, A, Y, 192; 1qfg, A, Y, 446; 1qfg, A, Y, 495; 1qfg, A, Y, 504; 1qfg, A, Y, 507; 1qfg, A, Y, 538; 1qfg, A, Y, 631; 1qfg, A, Y, 675; 1qfg, A, Y, 699; 1qgh, A, A, 18; 1qgh, A, A, 61; 1qgh, A, D, 125; 1qgh, A, E, 77; 1qgh, A, E, 86; 1qgh, A, F, 39; 1qgh, A, F, 146; 1qgh, A, G, 36; 1qgh, A, G, 69; 1qgh, A, H, 28; 1qgh, A, H, 43; 1qgh, A, K, 45; 1qgh, A, L, 13; 1qgh, A, L, 42; 1qgh, A, L, 150; 1qgh, A, M, 145; 1qgh, A, N, 14; 1qgh, A, P, 71; 1qgh, A, R, 63; 1qgh, A, T, 128; 1qgh, A, Y, 50; 1qov, M, F, 208; 1qov, M, G, 40; 1qov, M, G, 48; 1qov, M, G, 53; 1qov, M, G, 56; 1qov, M, G, 102; 1qov, M, G, 112; 1qov, M, G, 113; 1qov, M, G, 169; 1qov, M, G, 194; 1qov, M, G, 220; 1qov, M, G, 242; 1qov, M, G, 288; 1qov, M, N, 195; 1qov, M, P, 49; 1qov, M, P, 96; 1qov, M, P, 97; 1qov, M, P, 165; 1qov, M, P, 176; 1qov, M, P, 200; 1qov, M, Q, 46; 1qov, M, R, 132; 1qov, M, R, 253; 1qov, M, W, 185; 1qov, M, W, 297; 1qov, M, Y, 198; 1qvc, A, A, 35; 1qvc, A, E, 80; 1qvc, A, E, 100; 1qvc, A, G, 12; 1qvc, A, G, 15; 1qvc, A, G, 26; 1qvc, A, G, 74; 1qvc, A, G, 81; 1qvc, A, H, 55; 1qvc, A, L, 14; 1qvc, A, L, 71; 1qvc, A, N, 6; 1qvc, A, P, 18; 1qvc, A, R, 86; 1qvc, A, T, 36; 1qvc, A, T, 52; 1qvc, A, T, 85; 1qvc, A, T, 99; 1qvc, A, V, 8; 1qvc, A, W, 88; 1qvc, A, Y, 78; 1qvc, A, Y, 97; 1rpj, A, A, 27; 1rpj, A, A, 80; 1rpj, A, A, 155; 1rpj, A, A, 173; 1rpj, A, A, 205; 1rpj, A, A, 211; 1rpj, A, A,

243; 1rpj, A, C, 199; 1rpj, A, D, 44; 1rpj, A, D, 113; 1rpj, A, D, 202; 1rpj, A, E, 53; 1rpj, A, G, 22; 1rpj, A, G, 118; 1rpj, A, G, 141; 1rpj, A, G, 154; 1rpj, A, G, 207; 1rpj, A, G, 225; 1rpj, A, G, 239; 1rpj, A, G, 253; 1rpj, A, I, 85; 1rpj, A, L, 11; 1rpj, A, L, 87; 1rpj, A, L, 188; 1rpj, A, M, 204; 1rpj, A, M, 206; 1rpj, A, M, 260; 1rpj, A, N, 72; 1rpj, A, N, 114; 1rpj, A, P, 67; 1rpj, A, P, 249; 1rpj, A, R, 177; 1rpj, A, S, 69; 1rpj, A, S, 70; 1rpj, A, T, 244; 1rpj, A, V, 136; 1rpj, A, V, 208; 1rpj, A, V, 224; 1rpj, A, Y, 198; 1vfy, A, F, 183; 1vfy, A, G, 196; 1vfy, A, H, 190; 1vfy, A, H, 191; 1vfy, A, R, 193; 1vfy, A, R, 220; 1vin, A, A, 264; 1vin, A, A, 356; 1vin, A, A, 357; 1vin, A, D, 216; 1vin, A, D, 240; 1vin, A, E, 190; 1vin, A, E, 230; 1vin, A, E, 268; 1vin, A, E, 269; 1vin, A, E, 295; 1vin, A, E, 338; 1vin, A, F, 267; 1vin, A, F, 314; 1vin, A, G, 257; 1vin, A, K, 266; 1vin, A, K, 412; 1vin, A, L, 214; 1vin, A, L, 218; 1vin, A, L, 227; 1vin, A, L, 232; 1vin, A, L, 234; 1vin, A, L, 243; 1vin, A, L, 253; 1vin, A, L, 255; 1vin, A, L, 299; 1vin, A, L, 302; 1vin, A, L, 315; 1vin, A, M, 210; 1vin, A, M, 294; 1vin, A, P, 273; 1vin, A, P, 309; 1vin, A, P, 352; 1vin, A, Q, 203; 1vin, A, Q, 254; 1vin, A, Q, 317; 1vin, A, R, 211; 1vin, A, R, 241; 1vin, A, S, 353; 1vin, A, T, 231; 1vin, A, V, 221; 1vin, A, W, 217; 1vin, A, W, 372; 1vin, A, Y, 199; 1vin, A, Y, 286; 1vin, A, Y, 382; 1vin, A, Y, 413; 1who, A, F, 6; 1who, A, F, 64; 1who, A, G, 11; 1who, A, G, 47; 1who, A, L, 60; 1who, A, P, 63; 1who, A, P, 83; 1who, A, R, 67; 1who, A, S, 12; 1who, A, V, 8; 1who, A, V, 31; 1who, A, V, 81; 1who, A, V, 82; 1who, A, W, 41; 1who, A, W, 52; 1who, A, Y, 92; 1xca, A, D, 77; 1xca, A, E, 73; 1xca, A, F, 15; 1xca, A, F, 65; 1xca, A, F, 71; 1xca, A, G, 5; 1xca, A, G, 68; 1xca, A, N, 14; 1xca, A, P, 39; 1xca, A, Q, 97; 1xca, A, R, 29; 1xca, A, R, 79; 1xca, A, R, 132; 1xca, A, S, 12; 1xca, A, T, 56; 1xca, A, T, 75; 1xca, A, V, 24; 1xca, A, W, 7; 1xca, A, Y, 134; 1ytt, A, A, 146; 1ytt, A, C, 128; 1ytt, A, C, 195; 1ytt, A, C, 209; 1ytt, A, C, 217; 1ytt, A, E, 143; 1ytt, A, E, 218; 1ytt, A, F, 168; 1ytt, A, G, 158; 1ytt, A, G, 202; 1ytt, A, K, 110; 1ytt, A, N, 144; 1ytt, A, N, 180; 1ytt, A, P, 138; 1ytt, A, P, 186; 1ytt, A, W, 181; 1ytt, A, W, 204; 1ytt, A, Y, 178; 1zei, A, C, 7; 1zei, A, C, 19; 1zei, A, C, 39; 1zei, A, C, 52; 1zei, A, F, 24; 1zei, A, G, 8; 1zei, A, G, 23; 1zei, A, G, 33; 1zei, A, I, 34; 1zei, A, L, 15; 1zei, A, V, 12; 1zei, A, V, 35; 1zei, A, Y, 51; 2era, A, C, 3; 2era, A, C, 17; 2era, A, C, 24; 2era, A, C, 41; 2era, A, C, 43; 2era, A, C, 54; 2era, A, C, 55; 2era, A, C, 60; 2era, A, G, 40; 2era, A, N, 61; 2era, A, P, 44; 2era, A, R, 1; 2fgf, A, A, 75; 2fgf, A, A, 144; 2fgf, A, C, 92; 2fgf, A, E, 96; 2fgf, A, F, 94; 2fgf, A, F, 139; 2fgf, A, G, 38; 2fgf, A, G, 42; 2fgf, A, G, 61; 2fgf, A, G, 67; 2fgf, A, G, 80; 2fgf, A, G, 122; 2fgf, A, G, 127; 2fgf, A, I, 65; 2fgf, A, L, 23; 2fgf, A, L, 32; 2fgf, A, L, 82; 2fgf, A, L, 118; 2fgf, A, L, 140; 2fgf, A, M, 76; 2fgf, A, N, 104; 2fgf, A, P, 20; 2fgf, A, P, 141; 2fgf, A, S, 108; 2fgf, A, V, 63; 2fgf, A, Y, 103; 2fgf, A, Y, 106; 2fha, A, A, 66; 2fha, A, A, 99; 2fha, A, A, 103; 2fha, A, D, 126; 2fha, A, D, 171; 2fha, A, E, 17; 2fha, A, E, 27; 2fha, A, E, 61; 2fha, A, E, 62; 2fha, A, E, 107; 2fha, A, F, 41; 2fha, A, F, 55; 2fha, A, G, 77; 2fha, A, G, 78; 2fha, A, H, 65; 2fha, A, H, 118; 2fha, A, L, 48; 2fha, A, L, 104; 2fha, A, L, 114; 2fha, A, L, 138; 2fha, A, L, 155; 2fha, A, L, 175; 2fha, A, N, 21; 2fha, A, N, 25; 2fha, A, N, 111; 2fha, A, P, 88; 2fha, A, Q, 141; 2fha, A, R, 9; 2fha, A, R, 43; 2fha, A, R, 76; 2fha, A, S, 6; 2fha, A, Y, 32; 2fha, A, Y, 34; 2fib, A, A, 260; 2fib, A, A, 286; 2fib, A, A, 327; 2fib, A, C, 153; 2fib, A, C, 182; 2fib, A, C, 326; 2fib, A, D, 288; 2fib, A, D, 316; 2fib, A, E, 225; 2fib, A, E, 270; 2fib, A, F, 204; 2fib, A, F, 215; 2fib, A, F, 265; 2fib, A, F, 312; 2fib, A, G, 165; 2fib, A, G, 190; 2fib, A, G, 200; 2fib, A, G, 214; 2fib, A, G, 216; 2fib, A, G, 229; 2fib, A, G, 284; 2fib, A, G, 287; 2fib, A, G, 346; 2fib, A, G, 366; 2fib, A, H, 307; 2fib, A, H, 343; 2fib, A, I, 387; 2fib, A, K, 206; 2fib, A, L, 228; 2fib, A, L, 246; 2fib, A, L, 276; 2fib, A, L, 344; 2fib, A, M, 384; 2fib, A, N, 189; 2fib, A, N, 345; 2fib, A, P, 388; 2fib, A, Q, 195; 2fib, A, R, 197; 2fib, A, S, 237; 2fib, A, S, 332; 2fib, A, T, 314; 2fib, A, V, 180; 2fib, A, V, 193; 2fib, A, W, 191; 2fib, A, W, 208; 2fib, A, W, 227; 2fib, A, W, 334; 2fib, A, W, 335; 2fib, A, W, 369; 2fib, A, Y, 211; 2fib, A, Y, 262; 2fib, A, Y, 274; 2fib, A, Y, 348; 2gdm, A, A, 8; 2gdm, A, A, 37; 2gdm, A, A, 40; 2gdm, A, A, 64; 2gdm, A, A, 75; 2gdm, A, A, 113; 2gdm, A, A, 133; 2gdm, A, A, 137; 2gdm, A, E, 35; 2gdm, A, F, 46; 2gdm, A, F, 68; 2gdm, A, G, 94; 2gdm, A, K, 146; 2gdm, A, L, 3; 2gdm, A, L, 10; 2gdm, A, L, 47; 2gdm, A, L, 60; 2gdm, A, L, 78;

2gdm, A, M, 149; 2gdm, A, N, 19; 2gdm, A, Q, 7; 2gdm, A, Q, 77; 2gdm, A, S, 14; 2gdm, A, T, 117; 2gdm, A, V, 11; 2gdm, A, V, 109; 2gdm, A, W, 126; 2gdm, A, W, 134; 2liv, A, A, 10; 2liv, A, A, 16; 2liv, A, A, 29; 2liv, A, A, 62; 2liv, A, A, 85; 2liv, A, A, 142; 2liv, A, A, 155; 2liv, A, A, 237; 2liv, A, A, 283; 2liv, A, D, 50; 2liv, A, D, 51; 2liv, A, D, 121; 2liv, A, D, 146; 2liv, A, D, 195; 2liv, A, D, 319; 2liv, A, D, 323; 2liv, A, F, 332; 2liv, A, G, 13; 2liv, A, G, 19; 2liv, A, G, 25; 2liv, A, G, 37; 2liv, A, G, 75; 2liv, A, G, 93; 2liv, A, G, 125; 2liv, A, G, 151; 2liv, A, G, 153; 2liv, A, G, 200; 2liv, A, G, 201; 2liv, A, G, 207; 2liv, A, G, 238; 2liv, A, G, 243; 2liv, A, G, 314; 2liv, A, G, 322; 2liv, A, H, 76; 2liv, A, L, 115; 2liv, A, N, 34; 2liv, A, P, 84; 2liv, A, P, 99; 2liv, A, Q, 124; 2liv, A, Q, 212; 2liv, A, R, 116; 2liv, A, S, 79; 2liv, A, S, 86; 2liv, A, S, 294; 2liv, A, T, 102; 2liv, A, T, 107; 2liv, A, V, 73; 2liv, A, Y, 89; 2liv, A, Y, 150; 2liv, A, Y, 281; 2omf, A, A, 211; 2omf, A, A, 228; 2omf, A, D, 12; 2omf, A, D, 97; 2omf, A, D, 107; 2omf, A, D, 113; 2omf, A, D, 126; 2omf, A, D, 221; 2omf, A, D, 329; 2omf, A, E, 2; 2omf, A, E, 62; 2omf, A, F, 145; 2omf, A, F, 265; 2omf, A, F, 340; 2omf, A, G, 15; 2omf, A, G, 44; 2omf, A, G, 47; 2omf, A, G, 57; 2omf, A, G, 59; 2omf, A, G, 87; 2omf, A, G, 94; 2omf, A, G, 99; 2omf, A, G, 103; 2omf, A, G, 146; 2omf, A, G, 150; 2omf, A, G, 159; 2omf, A, G, 171; 2omf, A, G, 173; 2omf, A, G, 184; 2omf, A, G, 268; 2omf, A, K, 10; 2omf, A, K, 219; 2omf, A, K, 253; 2omf, A, L, 258; 2omf, A, N, 9; 2omf, A, N, 101; 2omf, A, N, 141; 2omf, A, N, 161; 2omf, A, N, 170; 2omf, A, N, 306; 2omf, A, N, 316; 2omf, A, P, 271; 2omf, A, Q, 156; 2omf, A, Q, 200; 2omf, A, R, 82; 2omf, A, R, 100; 2omf, A, R, 132; 2omf, A, R, 140; 2omf, A, R, 196; 2omf, A, S, 95; 2omf, A, T, 49; 2omf, A, T, 112; 2omf, A, T, 138; 2omf, A, Y, 106; 2omf, A, Y, 139; 2omf, A, Y, 180; 2omf, A, Y, 220; 2omf, A, Y, 226; 2omf, A, Y, 231; 2omf, A, Y, 263; 2omf, A, Y, 301; 2tct, A, A, 13; 2tct, A, A, 203; 2tct, A, D, 180; 2tct, A, E, 183; 2tct, A, E, 198; 2tct, A, F, 186; 2tct, A, F, 197; 2tct, A, G, 21; 2tct, A, G, 143; 2tct, A, G, 182; 2tct, A, G, 189; 2tct, A, G, 196; 2tct, A, H, 44; 2tct, A, I, 194; 2tct, A, K, 48; 2tct, A, L, 34; 2tct, A, L, 41; 2tct, A, L, 51; 2tct, A, L, 166; 2tct, A, L, 174; 2tct, A, L, 187; 2tct, A, L, 190; 2tct, A, L, 193; 2tct, A, L, 201; 2tct, A, R, 87; 2tct, A, W, 75; 2tdx, A, A, 31; 2tdx, A, A, 72; 2tdx, A, A, 101; 2tdx, A, E, 100; 2tdx, A, G, 52; 2tdx, A, G, 68; 2tdx, A, G, 129; 2tdx, A, I, 15; 2tdx, A, I, 132; 2tdx, A, L, 12; 2tdx, A, L, 53; 2tdx, A, L, 81; 2tdx, A, L, 86; 2tdx, A, L, 120; 2tdx, A, M, 64; 2tdx, A, P, 127; 2tdx, A, P, 133; 2tdx, A, R, 77; 2tdx, A, R, 80; 2tdx, A, T, 65; 2tdx, A, V, 123; 2tdx, A, Y, 11; 2tgi, A, A, 41; 2tgi, A, C, 15; 2tgi, A, C, 44; 2tgi, A, C, 48; 2tgi, A, C, 78; 2tgi, A, C, 109; 2tgi, A, C, 111; 2tgi, A, F, 24; 2tgi, A, G, 38; 2tgi, A, G, 46; 2tgi, A, I, 33; 2tgi, A, L, 83; 2tgi, A, L, 89; 2tgi, A, M, 104; 2tgi, A, P, 36; 2tgi, A, S, 80; 2tgi, A, V, 106; 2tgi, A, W, 32; 2tgi, A, Y, 39; 2tgi, A, Y, 90; 2tnf, A, A, 156; 2tnf, A, C, 101; 2tnf, A, D, 130; 2tnf, A, F, 64; 2tnf, A, F, 124; 2tnf, A, F, 152; 2tnf, A, G, 54; 2tnf, A, G, 121; 2tnf, A, G, 129; 2tnf, A, G, 153; 2tnf, A, H, 15; 2tnf, A, H, 78; 2tnf, A, L, 26; 2tnf, A, L, 43; 2tnf, A, L, 76; 2tnf, A, L, 93; 2tnf, A, L, 126; 2tnf, A, L, 132; 2tnf, A, L, 157; 2tnf, A, N, 46; 2tnf, A, P, 51; 2tnf, A, Q, 61; 2tnf, A, S, 60; 2tnf, A, S, 86; 2tnf, A, W, 28; 2tnf, A, W, 114; 2tnf, A, Y, 56; 2tnf, A, Y, 59; 2vpf, A, C, 26; 2vpf, A, C, 51; 2vpf, A, C, 57; 2vpf, A, C, 61; 2vpf, A, C, 68; 2vpf, A, C, 102; 2vpf, A, C, 104; 2vpf, A, E, 38; 2vpf, A, F, 96; 2vpf, A, H, 99; 2vpf, A, R, 56; 2vpf, A, S, 24; 2vpf, A, V, 33; 2vpf, A, V, 52; 3fis, A, A, 77; 3fis, A, A, 78; 3fis, A, E, 59; 3fis, A, K, 91; 3fis, A, L, 88; 3fis, A, M, 81; 3fis, A, Q, 74; 3fis, A, T, 87; 3gbp, A, A, 188; 3gbp, A, A, 215; 3gbp, A, A, 218; 3gbp, A, A, 221; 3gbp, A, D, 40; 3gbp, A, D, 212; 3gbp, A, D, 257; 3gbp, A, F, 89; 3gbp, A, G, 6; 3gbp, A, G, 109; 3gbp, A, G, 116; 3gbp, A, G, 120; 3gbp, A, G, 148; 3gbp, A, G, 217; 3gbp, A, G, 234; 3gbp, A, G, 248; 3gbp, A, G, 252; 3gbp, A, I, 204; 3gbp, A, L, 146; 3gbp, A, L, 176; 3gbp, A, M, 214; 3gbp, A, N, 66; 3gbp, A, N, 210; 3gbp, A, N, 302; 3gbp, A, P, 86; 3gbp, A, P, 94; 3gbp, A, Q, 45; 3gbp, A, Q, 48; 3gbp, A, Q, 51; 3gbp, A, Q, 119; 3gbp, A, Q, 261; 3gbp, A, T, 3; 3gbp, A, T, 159; 3gbp, A, T, 253; 3gbp, A, V, 7; 3gbp, A, V, 68; 3gbp, A, V, 108; 3gbp, A, V, 254; 3gbp, A, Y, 10; 3gbp, A, Y, 107; 3gbp, A, Y, 143; 3gbp, A, Y, 295; 451c, A, A, 31; 451c, A, A, 40; 451c, A, G, 51; 451c, A, L, 6; 451c, A, L, 79; 451c, A, W, 77
